# Synthesis and Biological Screening of New Lawson Derivatives as Selective Substrate‐Based Inhibitors of Cytochrome *bo_3_* Ubiquinol Oxidase from *Escherichia coli*

**DOI:** 10.1002/cmdc.201900707

**Published:** 2020-04-14

**Authors:** Isam Elamri, Melanie Radloff, Katharina F. Hohmann, Vijaykumar D. Nimbarte, Hamid R. Nasiri, Michael Bolte, Schara Safarian, Hartmut Michel, Harald Schwalbe

**Affiliations:** ^1^ Center for Biomolecular Magnetic Resonance Institute of Organic Chemistry and Chemical Biology Goethe-Universität Frankfurt am Main Max-von Laue-Straße 7 60438 Frankfurt am Main Germany; ^2^ Department of Molecular Membrane Biology Max Planck Institute of Biophysics Max-von-Laue-Straße 3 60438 Frankfurt am Main Germany; ^3^ Institute for Inorganic Chemistry Goethe-Universität Frankfurt am Main Germany

**Keywords:** alkylation, cytochromes, hydroxynaphthoquinone, inhibitor design, oxidases, reductases

## Abstract

The respiratory chain of *Escherichia coli* contains two different types of terminal oxidase that are differentially regulated as a response to changing environmental conditions. These oxidoreductases catalyze the reduction of molecular oxygen to water and contribute to the proton motive force. The cytochrome *bo*_3_ oxidase (cyt *bo*_3_) acts as the primary terminal oxidase under atmospheric oxygen levels, whereas the *bd*‐type oxidase is most abundant under microaerobic conditions. In *E. coli*, both types of respiratory terminal oxidase (HCO and *bd*‐type) use ubiquinol‐8 as electron donor. Here, we assess the inhibitory potential of newly designed and synthesized 3‐alkylated Lawson derivatives through L‐proline‐catalyzed three‐component reductive alkylation (TCRA). The inhibitory effects of these Lawson derivatives on the terminal oxidases of *E. coli* (cyt *bo*_3_ and cyt *bd*‐I) were tested potentiometrically. Four compounds were able to reduce the oxidoreductase activity of cyt *bo*_3_ by more than 50 % without affecting the cyt *bd‐*I activity. Moreover, two inhibitors for both cyt *bo*_3_ and cyt *bd*‐I oxidase could be identified. Based on molecular‐docking simulations, we propose binding modes of the new Lawson inhibitors. The molecular fragment benzyl enhances the inhibitory potential and selectivity for cyt *bo*_3_, whereas heterocycles reduce this effect. This work extends the library of 3‐alkylated Lawson derivatives as selective inhibitors for respiratory oxidases and provides molecular probes for detailed investigations of the mechanisms of respiratory‐chain enzymes of *E. coli*.

## Graphical abstract

**To** ***b*** **or not to** ***b***: Alkylated hydroxynaphthoquinone derivatives, synthesized by reductive alkylation and tested for their inhibitory effect on bacterial respiratory oxidases, showed selectivity for one particular cytochrome *b* oxidase. Enzyme activity assays and docking analysis provided insights into their binding site. Alkylation with cyclic residues like benzyl groups only increased the inhibitory effect of one oxidase.

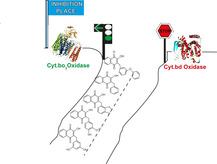

## Introduction

Naphthoquinones are among the most abundant compounds in nature.^1^ The growing interest to develop novel quinone derivatives for targeting diverse biomolecular processes originates from their role as essential metabolites in living organisms, for example, phylloquinone (vitamin K_1_) in green plants or ubiquinols and menaquinols in bacteria.^2^ The naphthalene‐based 1,4‐diketone compounds are interacting with a broad spectrum of biological targets through two different mechanisms, by either forming covalent bonds due to their electrophilic properties or reducing oxygen.^3^, ^4^ Derivatization of the 1,4‐naphthoquinones scaffold into novel inhibitors opens potential avenues to increase the possibility of interaction with biological molecules, in particular also to gain target specificity^5^, ^6^, ^7^ Consequently, the inhibitory properties of 1,4‐naphthoquinones and their derivatives have been elucidated in multiple studies presenting, for example, antimalarial,^8^, ^9^, ^10^ anticancer,^11^, ^12^, ^13^ antibacterial,^14^, ^15^ antiseptic or cytotoxic^16^, ^17^ activities. The respiratory chain of E. coli performs redox chemistry by exclusively using quinols as electron carriers. Key enzymes are the quinol oxidizing cytochrome *bd‐I*, *bd‐II* and *bo*_3_ oxidases. At atmospheric oxygen conditions (250 μM O_2_), the cyt *bo*_3_ oxidase is the primary terminal oxidase (*K*_m_=2.4 μM). Under limited oxygen concentrations cytochrome *bd*‐type oxidases are essential for maintaining aerobic respiration due to their higher affinity for oxygen (*K*_m_=3–500 nM).^18^ The stoichiometry of the four electron reduction of O_2_ through oxidation of membrane bound ubiquinol‐8 (UQ8H_2_) to ubiquinone‐8 (UQ8) by cyt *bo*_3_ is shown in the following equation:

2UQ8H2+8Hcyto++O2←→2UQ8+8Hperi++2H2O

The free energy of this redox reaction is utilized to pump four protons from the cytoplasm to the periplasm while four substrate protons from the cytoplasm are used for the reduction of dioxygen to water.^18^, ^19^ Based on their electron donors, members of the heme‐copper oxidases superfamily (HCO) are divided into two classes. Quinol‐type oxidases (QOX) use membrane‐soluble quinol molecules as two‐electron donors while cytochrome *c*‐type oxidases (COX) receive their electrons from membrane associated cytochrome *c* proteins.^20^, ^21^, ^22^, ^23^ The *E. coli* cyt *bo*_3_ oxidase is the best studied member of the QOX subfamily and is characterized by four‐subunits. Subunits I, II and III are homologues to the core subunits of the mitochondrial A‐type cytochrome *c* oxidase.^22^, ^24^ Cytochrome *bd*‐type oxidases show no sequence homology to members of the heme‐copper superfamily.^2^, ^25^, ^26^ Recently, several studies reported the ability of cyt *bo*_3_ to use low potential quinones, menaquinones (MKs) and demethylmenaquinones (DMKs) in their reduced form as electron donors under anaerobic conditions.^27^, ^28^, ^29^, ^30^, ^31^, ^32^ Despite the high structural similarity to these quinone substrates, the inhibitory effects of naphthoquinone derivatives on the cytochrome *bo3* (cyt *bo*_3_) oxidase have not been performed in the past. Only non‐selective Aurachin and hydroxyquinoline N‐oxide derivatives (e. g., 2‐heptyl‐4‐quinolinol 1‐oxide, HQNO) have been used to investigate the functional and structural properties of quinol binding and oxidation.

Here, we describe synthesis and in vitro analysis of new 3‐alkylated hydroxynaphthoquinones using l‐proline‐catalyzed three‐component reductive alkylation. Within this set of compounds, we have identified new inhibitors with target selectivity for cyt *bo*_3_ of *E. coli*.

## Synthesis

**Scheme 1:**
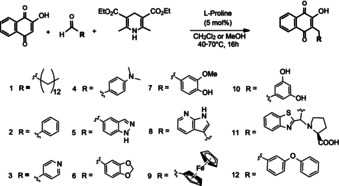
Synthesis of 3‐alkylated hydroxynaphthoquinone derivatives (HNQ‐1‐12) by TCRA. Hydroxynaphthoquinones (1 equiv), Hantzsch ester (1 equiv), l‐proline (0.5 equiv). Solvent: CH_2_Cl_2_ or MeOH for poorly soluble aldehyde derivatives. In some cases, the crude product was stirred for 4 h with LiOH (4 equiv) in H_2_O/MeOH to remove the excess oxidized Hantzsch ester (saponification). The general TCRA procedure is described in the Experimental Section. Characterization of data is shown in Figures S2–S36 in the Supporting Information.

We synthesized a new set of alkylated hydroxynaphthoquinones and probed their activity and selectivity against cyt *bo*_3_ and *bd‐I‐type* oxidases from *E. coli*. We aimed to synthesize a systematic set of new 3‐alkylated hydroxynaphthoquinones (HNQ) using l‐proline‐catalyzed three‐component reductive alkylation (TCRA; Scheme 1).

TCRA was described previously by Ramachary^23^, ^33^, ^34^ as an efficient procedure to substitute cyclic β‐keto‐lactones with different aldehyde derivatives. Gribble et al.^35^ have shown that the reaction yield can be significantly improved upon addition of two equivalents of corresponding aldehyde. Alkylation of HNQ scaffold using the TCRA method showed high alkylation rates, despite the expected mesomerization in presence of the aromatic benzene ring and the 2‐hydroxy group, yielding up to 80 % of desired 3‐alkylated Lawson‐derivatives. However, in case of benzothiazol‐2‐carbaldehyde derivative (Scheme 1, HNQ‐11), the reaction yield was considerably lower. Analysis of the reaction by TLS‐MS spectrometry showed that proline‐intermediate [**II**] was the dominating part in the reaction mixture. Attempts to force the equilibrium to the olefin‐intermediate [**III**], either by increasing the quantity of the reduction agent (Hantzsch ester) or of the catalyst (l‐proline), did not prove to be a successful approach. However, we were able to isolate the proline‐intermediate [**II**] from this reaction using column chromatography as HNQ‐11 (Scheme 1).

**Scheme 2:**
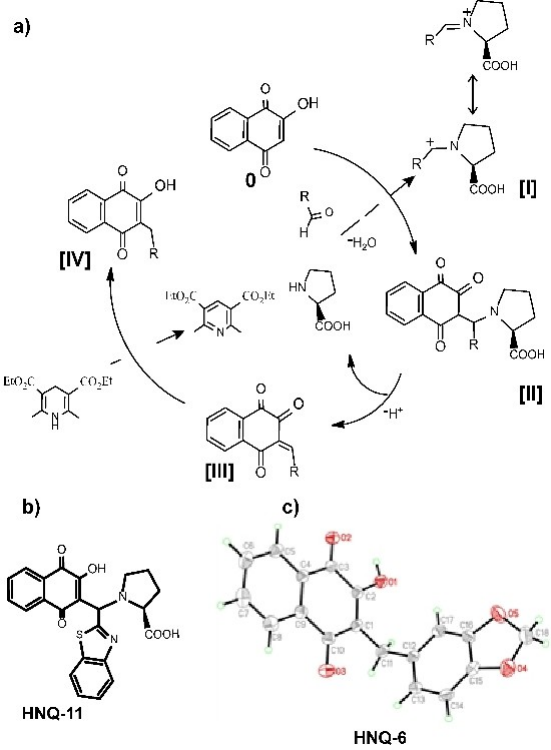
a) Hypothetical mechanism for the catalytic cycle of TCRA to alkylated Lawson. b) Isolated HNQ‐11 confirming intermediate [II]. c) Atomic model of the X‐ray structure of HNQ‐6 confirming the alkylated Lawson. Crystallographic data of HNQ‐6 are shown in Figure S1.

This confirmed the hypothesized feasibility of the TCRA alkylation mechanism for reductive alkylation of 2‐hydroxynaphthoquinone, as shown in Scheme 2. The multiple‐component cascade reaction starts with activation of the aldehyde to the corresponding iminium intermediate [**I**], which can undergo a nucleophilic attack from the double bond of the vinyl alcohol of Lawson **0** under very mild conditions, generating the substituted naphthalenetrione‐intermediate *in situ* [**II**]. Due to high acidity of [**II**] a reductive elimination reaction occurs, giving a 3‐alkylated olefin [**III**], which undergoes the redox reaction with Hantzsch ester, forming the corresponding hydrogenated product [**IV**] (Scheme 2a). The hypothesized mechanism of this reaction was supported by the inadvertently isolated intermediate HNQ‐11 and the crystal structure of HNQ‐6 (Scheme 2b and c).

## Solubility assay

**Scheme 3:**
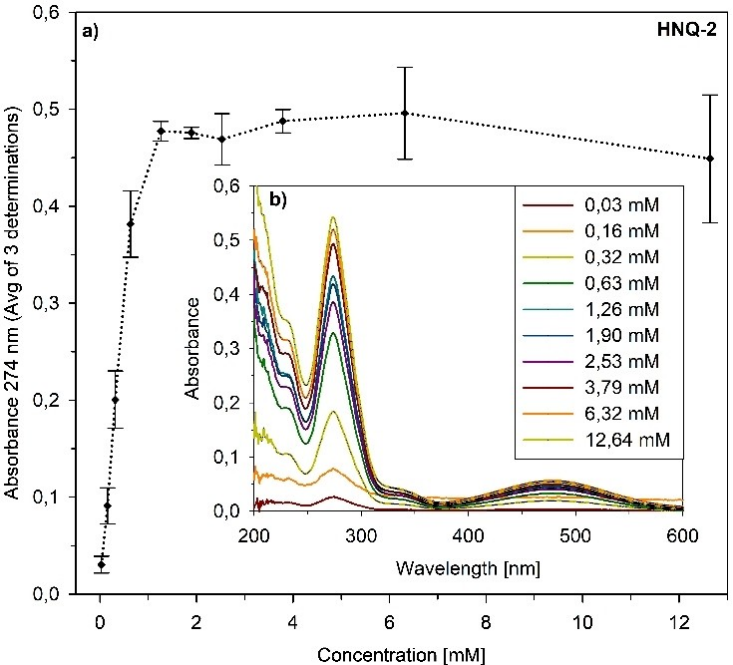
a) Solubility profile of HNQ‐2 in buffer (20 mM NaPi, 50 mM NaCl, 0.02 % DDM, pH 8.0) at room temperature and detection wavelength λ=274 nm. b) Absorbance spectra of HNQ‐2 for all concentrations used in the assay.

To meet the requirements for further functional investigations, the solubilities of the HNQ compounds were determined by an absorbance‐based thermodynamic equilibrium solubility assay.^36^ As shown in Scheme 3a, plotting absorbance over concentration at the wavelength of the maximum peak of the absorbance spectra, (HNQ‐2 at λ=274 nm), provides a saturation point at 1.26±0.03 mM (solubility graphs for all HNQ compounds are shown in Figure S37). Table 1 indicates that solubility of most evaluated compounds ranges between 0.87 mM and 5.48 mM, whereas three of them show solubility higher than 8.3 mM or even 10.3 mM in buffer. Therefore, all twelve HNQ compounds are feasible for further functional studies.

**Table 1:** Experimental solubility data of HNQ‐1 – HNQ‐12 in buffer (20 mM NaPi, 50 mM NaCl, 0.02 % DDM, pH 8.0) at room temperature. Stock concentrations and concentrations for particular measurements are shown in Table S1.

Compound	Detection Wavelength [nm]	Solubility [μg/mL]	Solubility [mM]
HNQ‐1	273	335±24	1.02±0.08
HNQ‐2	274	334±7	1.26±0.03
HNQ‐3	470	627±21	2.36±0.08
HNQ‐4	275	267±19	0.87±0.06
HNQ‐5	273	1669±69	5.5±0.3
HNQ‐6	274	400±44	0.78±0.09
HNQ‐7	479	>2677	>8.3
HNQ‐8	275	>3135	>10.3
HNQ‐9	273	>2677	>8.3
HNQ‐10	490	840±151	2.1±0.4
HNQ‐11	275	800±85	1.84±0.19
HNQ‐12	273	1640±94	4.6±0.3

## Enzyme activity assay

**Figure 1:**
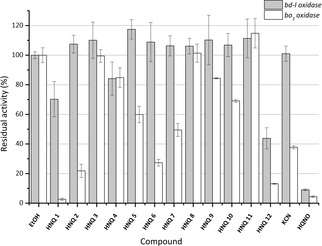
Screening of the inhibition potentials of tested hydroxynaphthoquinones (HNQ) on the oxidase reduction activity of purified *E. coli cytbo_3_* (white) and bd‐I (light gray) oxidase. Inhibition assay using HNQs at 250 μM in the presence of 200 μM ubiquinone‐1 and 5 mM dithiothreitol. Oxygen reduction activity was calculated from oxygen consumption rates at 30 nM enzyme concentration at RT for each experiment. 100 % activity=adding corresponding volume of EtOH. KCN (20 μM) and HQNO (250 μM) represent positive inhibition controls for cyt bo_3_. The data are mean values of three independent experiments ± standard deviation.

The inhibitory effect of a set of HNQs was screened in micromolar range to identify selective cyt *bo_3_* and cyt *bd‐I* inhibitors. Oxygen reduction was measured via a Clark‐type electrode. Turnover rates of cyt *bo_3_* and *bd*‐I experiments are presented as relative activities (Figure 1). For experimental overview and further information see Supporting Information (Figures S38–S40), Tables S2–3). Activity of cyt *bo*_3_ was strongly (>50 %) reduced by HNQ‐1, ‐2, ‐6, ‐7 and ‐12. HNQ‐1 and HNQ‐12 are the most effective cyt *bo*_3_ inhibitors, yet lack selectivity as they also inhibit cyt *bd‐I*, but in a lower extend compared to HQNO. HQNO is described as a noncompetitive^37^ or uncompetitive^38^, ^39^ inhibitor. Overall, the *E. coli bd‐I‐type* oxidase was less sensitive to the majority of examined compounds. Merely the two most hydrophobic substances showed an inhibitory effect on the cyt *bd‐I*‐type oxidase (HNQ‐1: 30 %; HNQ‐12: 56 %).

No or a low inhibitory effect (<30 %) was observed for HNQ‐3, ‐4, ‐8, ‐9 and ‐11 with both oxidases. With respect to cyt *bo*_3_ selectivity HNQ‐2, ‐6 and ‐7 are the most promising candidates for further rounds of chemical optimization.

**Figure 2:**
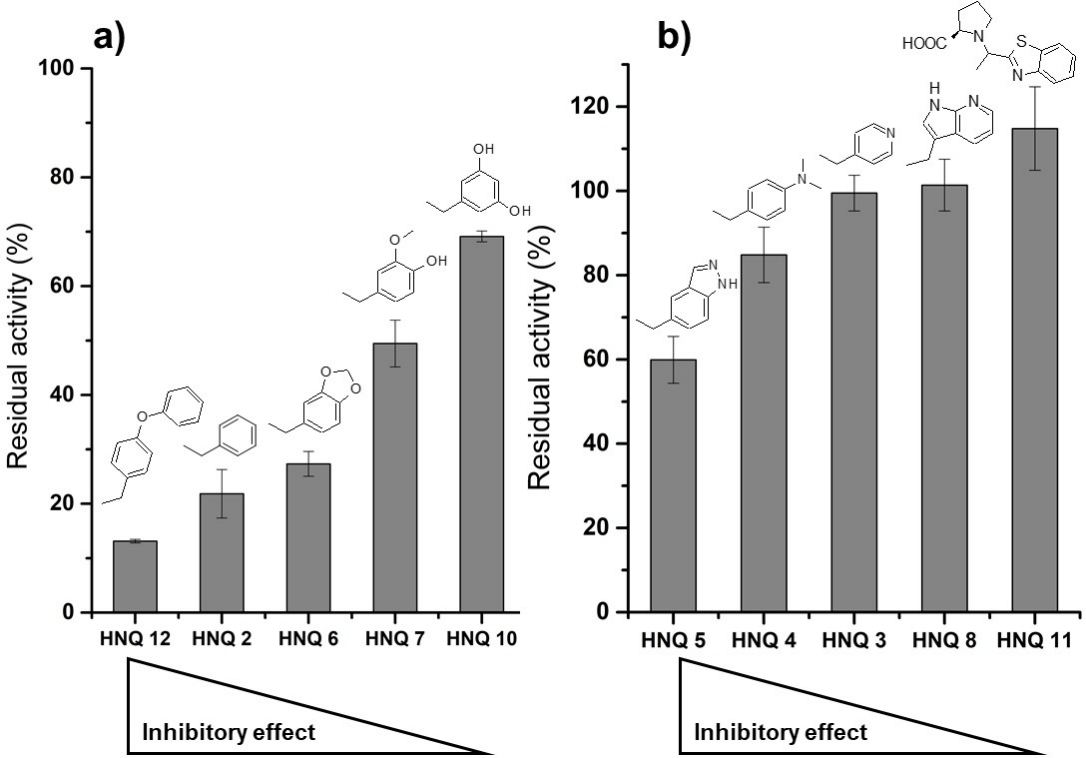
Overview of relative inhibition of benzylic and a) alkoxy‐ or hydroxy‐containing residues and b) nitrogen containing residues on the oxidase reduction activity of *E. coli cyt bo_3_* with relevant residues in position 3.

In order to understand the correlation between inhibition and the chemical nature of the alkylated residue at position 3 of the HNQ backbone, we systematically grouped our compounds based on the substituents and comparatively evaluated their effects (Figure 2). An overview of the effect of structural modifications on the inhibition potential of oxygen reduction activity is given in Table 2. For residues composed of a benzylic ring in combination with an alkoxy or hydroxy group a decrease in inhibition is noticeable depending on position and arrangement of the heteroatoms (from HNQ‐12 over ‐2, ‐6, ‐7 to ‐10) (Figure 2 A). Benzylated HNQ‐2 showed a strong inhibitory effect on cyt *bo*_3_, while not influencing the cyt *bd‐I* activity at all. Compounds HNQ‐6 and ‐7 share a positive mesomeric effect (+M) due to the aromatic character of their residues. The benzodioxole of HNQ‐6, enhanced the inhibition of cyt *bo*_3_ activity (<30 % rest activity for cyt *bo_3_*) while also showing an increased selectivity for the HCO‐type oxidase. Changing the dioxolan ring to a hydroxy and a methoxy group in HNQ‐7 slightly reduces the inhibitory effect on cyt *bo*_3_ (<50 % residual activity for cyt *bo_3_*) and also negatively affects the selectivity compared to HNQ‐6. HNQ‐10 contains a resorcin residue and shows a low inhibition of 31 %. This suggests that addition of two hydroxy groups lowers the inhibitory effect on cyt *bo*_3_ compared to the simply benzylated HNQ‐2. It is conceivable that the negative inductive effect (−I) of the OH‐groups lowers the aromatic π‐π system in HNQ‐10 as this group possibly interacts with the amino acids of the cyt *bo*_3_ quinol binding site.

**Table 2:** Effect of structural hydroxynaphthoquinone modification on the inhibition potential of purified *E. coli* cyt *bo_3_* and *bd‐I* oxidase.

Structural inhibitor modification	HNQ	Effect on inhibition	
		*bo_3_*	*bd*
2‐OH group instead of 2‐Me group of quinone substrates	1–12	+++	–
alkylation with long chain or bulky residues	1, 12	+++	++
simple benzylation^[a]^	2	+++	–
benzylation with aromatic substituents that exhibit a positive mesomeric effect	6, 7	++	–
Benzylation with aromatic substituents that exhibit a negative inductive effect	10	–	–
Alkylation with nitrogen heterocycles	3–5, 8, 11	–	–

[a] The attempt to increase the inhibition effect on cyt *bo_3_* through introducing a phenoxy group (HNQ‐12) containing two phenyl groups was successful but at the expense of selectivity since inhibition of *bd*‐oxidase was also noticeably increased.

A similar effect was observed for the nitrogen containing moieties in HNQ‐3, ‐4, ‐5, ‐8 and ‐11. HNQ‐11 is characterized by a bulky residue composed of a benzothiazole and proline that lacks the methylene spacer to the HNQ backbone. HNQ‐8 contains a 7‐azaindol group. We could not show an influence on either cyt *bo*_3_ or *bd‐I‐type* oxidase activity with these compounds. In HNQ‐3 a pyridine group was added that seems to impede an interaction with both enzymes because the activity remains unaffected. Changing this group to *N*,*N*‐dimethylaniline, as in HNQ‐4, improved the inhibitory effect on both enzymes (15 % rest activity for cyt *bo_3_* and cyt *bd‐I*) but did not improve selectivity for cyt *bo_3_*. For HNQ‐5 a modification of the *N*,*N*‐dimethylaniline to a 1‐H‐indazole residue results in a higher selectivity for cyt *bo*_3._ HNQ‐5 shares a bulky residue similar to HNQ‐8 composed of two rings including two nitrogens as heteroatoms. However, in HNQ‐5 the benzylic‐part of the 1‐H‐indazole group is linked to the hydroxynaphthoquinone backbone instead of the C5 heterocycle of HNQ‐8.

Interestingly, whereas HNQ‐5 shows a selective inhibitory effect on cyt *bo*_3_, HNQ‐8 does not affect oxygen reductase activities at all (40 % inhibition HNQ‐5; 0 % inhibition HNQ‐8). Concerning the poor inhibitory effect of the heteroaromatic compound HNQ‐3 it is reasonable that the heteroaromatic character of the pyridine residue hinders interaction with cyt *bo*_3_.

Our experiments revealed that alkylation of hydroxynaphthoquinones with nitrogenous heterocycles, for example; benzamine (HNQ‐4), indazole (HNQ‐5), azaindole (HNQ‐8) and benzothiazole (HNQ‐11) is not expedient to inhibit neither cyt *bo*_3,_ nor cyt *bd‐I* activity.

Substituted benzenes groups showed an improved inhibitory effect since this seems to show a certain level of tolerance towards electron‐donating groups, for example, alkoxy in HNQ‐6 or HNQ‐12. This effect diminishes by introducing a hydroxy group (HNQ‐7) or regarding HNQ‐10 a double hydroxylation. HNQ‐9 represents the only tested metallocene. This feature did not improve the inhibitory effect nor selectivity compared to metal‐free HNQs.

To gain further insights into inhibition efficiencies, the apparent ki (*k*_i_^app^) values for HQNO, HNQ‐12 and HNQ‐6 were determined.^37^, ^40^ The assay was performed without preincubation of enzyme and inhibitor and with a fixed ubiquinone‐1 concentration of 200 μM. For HQNO *k*_i_^app^ was found to be in a micromolar range at 3.2±0.7 μM. In case of HNQ‐12 *k*_i_^app^ was determined on a similar scale at 5.7±0.6 μM. The *k*_i_^app^ for the third tested compound HNQ‐6 was found to be two orders of magnitude higher at 225±9 μM (Figures S41–S43). Previous work determined a *k*_i_^app^ for HQNO at 0.7 μM through steady state kinetic experiments.^40^

## Molecular docking studies

To explore molecular binding interactions with cyt *bo*_3_ we subsequently performed in‐depth docking analyses for HNQ‐2, HNQ‐6 and HNQ‐7, respectively.

Ubiquinol cyt *bo*_3_ oxidase crystal structure is composed of tetramer subunits chain‐A, chain‐B, chain‐C, chain‐D, chain‐E and chain‐F, respectively with a sequence length of 663 amino acids. Most frequently used inhibitors for cyt *bo*_3_ are the non‐competitive HQNO (2‐*n*‐heptyl‐4‐hydroxyquinoline *N*‐oxide) and different Aurachin C derivatives.^37^, ^40^, ^41^, ^42^, ^43^ Previous work by Choi et al.^40^ explored *E. coli* cyt *bo*_3_ with the natural substrate ubiquinol‐8 and known inhibitors Aurachin C derivatives as well as HQNO (2‐*n*‐heptyl‐4‐hydroxyquinoline *N*‐oxide) suggested that there is only one binding site for ubiquinol, a high‐affinity single‐site (Q_H_), which disprove the current consensus model of two binding sites (Q_H_, Q_L_). Furthermore, it was confirmed that inhibitors such as HQNO bind also on the Q_H_ site displacing only the quinone part of the endogenous substrate due the high affinity of the isoprene chain to the protein.^40^

**Figure 3:**
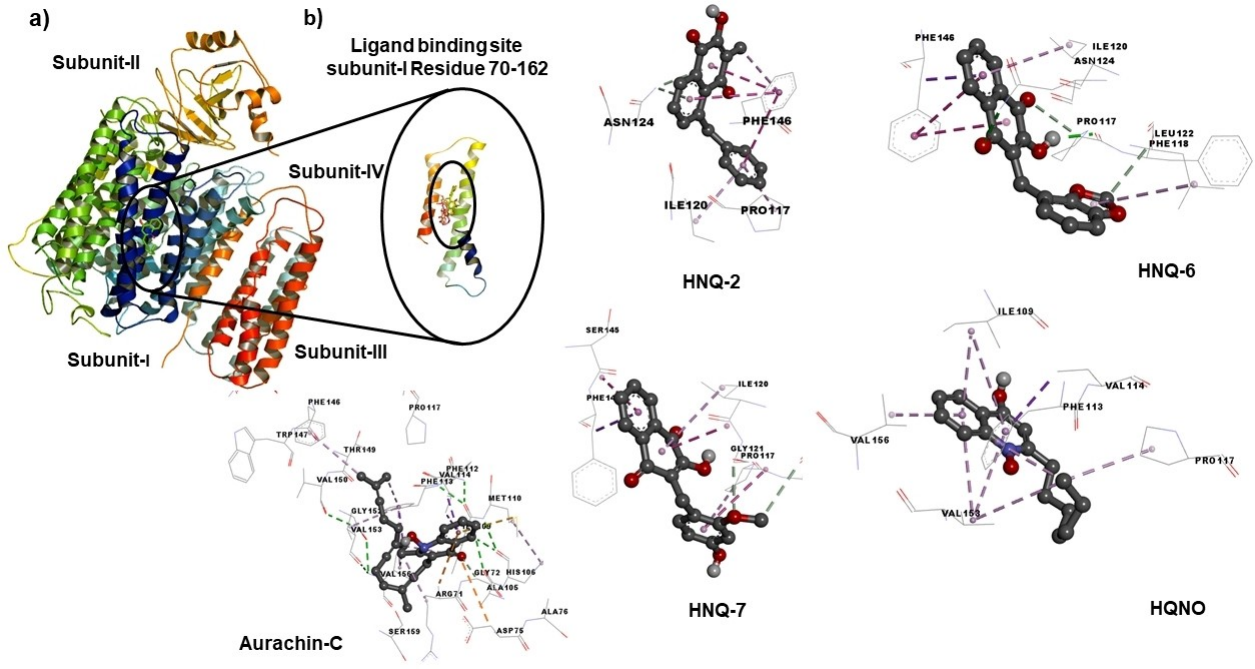
a) Proposed binding‐site of conjugate HNQ‐2, HNQ‐6 and HNQ‐7 within the subunit‐1 of cyt *bo_3_* ubiquinol oxidase (PDB ID: 1FFT); b) 2D‐interactions of HNQ‐2 with ASN‐A‐124 (π donor in cyan), Phe‐A‐146 (π‐π‐stacking interactions in pink), Ile‐A‐120 and Pro‐A‐117 (π‐alkyl interactions in light pink); 2D interaction of HNQ‐7 with Phe‐A‐146 (π‐σ interactions in violet), Ser‐A‐145, Ile‐A‐120 (amide‐π stacked in violet), Gly‐A‐121, Phe‐A‐118 (C−H donor in cyan) and with Pro‐A‐117 (π‐alkyl interactions in pink); 2D interactions of HQNO with Val‐A‐114 (π‐σ interactions in violet) and with Ile‐A‐109, Phe‐A‐113, Pro‐A‐117, Val‐A‐153, Val‐A‐156 (π‐alkyl interactions in light pink); 2D interactions of Aurachin C with Arg‐A‐71 (π‐cation interactions in yellow), Val‐A‐114 (π‐σ interactions in violet), Met‐A‐110 (π‐sulfur interactions in yellow), and Ile‐A‐109, Phe‐A‐113, Pro‐A‐117, Phe‐A‐146, Val‐A‐153, Val‐A‐156, Ile‐A‐109 (π‐alkyl interactions in light pink), and with Gly‐A‐72 (C−H bond interactions in light green).

**Figure 4:**
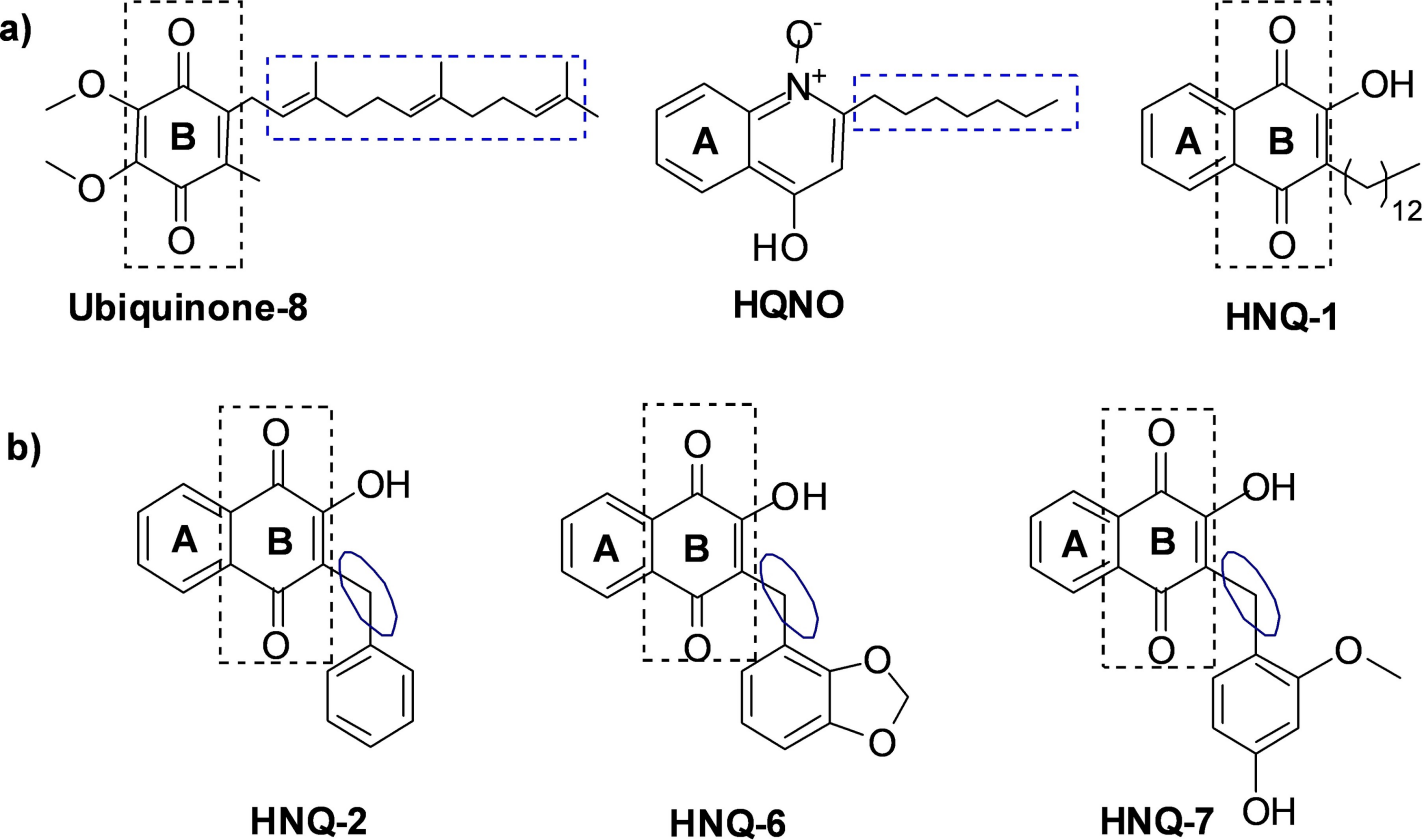
a) Primary design of HNQ‐1 inspired from the combination of substrate ubiquinol‐8 and inhibitor HQNO. The boxes suggest that hydroxynaphthoquinones are a combination of ubiquinol‐8 and HQNO . b) Modified hydroxynaphthoquinones.

In‐depth docking analysis has been performed to elucidate the interactions of the most active agents HNQ‐2, HNQ‐6 and HNQ‐7 and HQNO, Aurachin C1–10 as reference inhibitors respectively at the Q_H_ site of cyt *bo*_3_ ubiquinol oxidase (PDB ID: 1FFT) by using Autodock 4.2.^21^ Interestingly, we observed that despite structural differences, the quinolinone ring of Aurachin C1–10 interacts with the same two residues like the hydroquinone ring of ubiquinol‐2, namely Arg71 and Asp75. It is likely that the binding sites for Aurachin C and ubiquinol‐2 are partially overlapping. Further, by comparing docking of all three inhibitor classes it could be found that the binding mode of HNQ‐derivatives is more similar to that of HQNO than of Aurachin C. Aurachin C interacts with residues in the substrate binding loop (Figures 3 and S47). Our designed set of hydroxynaphthoquinones inhibitors were inspired from the native substrate ubiquinol‐8 (ring **B** in Figure 4) and HQNO (ring **A** in Figure 4). During the development of diverse set of ligands several modifications were introduced into the basic scaffold.

The first derivative HNQ‐1 exhibits a long aliphatic side chain, which resembles to the side chain of HQNO and substrate ubiquinol‐8. The results of enzyme assays suggest that the scaffolds with relatively long aliphatic side chain are nonselective in nature. To investigate it further we performed intensive molecular docking studies and found that the aliphatic side chains are involved in steric hindrance and some unconventional intermolecular bumps were observed in HNQ‐1 and HQNO. This might be the reason for weak binding of HNQ‐1 and HQNO into the hydrophobic pocket of the cyt *bo*_3_ ubiquinol oxidase (PDB ID: 1FFT). Further docking analysis were performed on the most promising derivatives HNQ‐2, HNQ‐6 and HNQ‐7, which showed selective inhibition of cyt *bo_3_*. The binding energies presented in Table 3 support results of enzyme activity. HNQ‐2, ‐6, ‐7 showed a lower binding energy in comparison with HQNO and Aurachin C1–10.

**Table 3:** Binding energies for best fives conformations of HNQ‐derivatives and HQNO and Aurachin C1–10 for cyt *bo_3_*. For remaining HNQ‐derivatives, see Supporting Information Table S4.

Binding energies kcal/mol					
HQNO	−5.72	−5.41	−5.32	−5.24	−5.19
Aurachin C1–10	−4.7	−4.65	−4.01	−3.96	−3.65
HNQ‐2	−6.69	−6.68	−6.58	−6.63	−6.18
HNQ‐6	−6.95	−6.81	−6.65	−6.49	−6.22
HNQ‐7	−6.44	−6.22	−6.35	−6.28	−6.05

*Interactions of HNQ‐2*: We found that for HNQ‐2, the 3‐hydroxynaphthalene‐1,4‐dione central scaffold is involved in the π‐π stacking (pink) interaction with Phe‐A‐146. In addition, unconventional π‐donor hydrogen binding interactions (cyan) were observed with ASN‐A‐124. The phenyl ring with methylene spacer attached to central ring gives an opportunity to rotate freely and found to be involved in the π‐π stacking interactions (pink) with the Phe‐A‐146 and some π‐alkyl interactions (light pink) with Ile‐A‐120 and Pro‐A‐117 respectively (Figures 3 and S44).

*Interactions of HNQ‐6*: Molecular docking insight of HNQ‐6 suggests that the central naphthalene ring is involved in π‐σ interactions (violet), π‐π stacking interactions (pink) and π‐alkyl interactions (light pink) with Phe‐A‐146 and Ile‐A‐120 respectively. In addition to this conventional hydrogen interactions (green) and unconventional π‐donor hydrogen binding interactions (cyan) were observed in 1,4‐dione carbonyl and hydroxy moiety of HNQ‐6 with ASN‐A‐124 and Pro‐A‐117 respectively. The 1,3‐benzodioxole residues attached to the HNQ‐ring gives an opportunity to rotate freely and found to be involved in the π‐alkyl interactions (light pink) with the LEU‐A‐122 and unconventional π‐donor hydrogen binding interactions (cyan) with Phe‐A‐118 (Figures 3 and S44).

*Interactions of HNQ‐7*: Hydroxynaphthoquinone HNQ‐7 is involved in the π‐π stacking (pink) and π‐alkyl (light pink) with Ile‐A‐128, Phe‐A‐146, SER‐A‐145. The methoxy hydroxy ring is involved in the unconventional hydrogen binding interactions (light green) in between methoxy group and Gly‐A‐121 and Phe‐A‐118. In addition to this pi electron cloud of substituted phenyl ring is involved in π‐π stacking interaction with Pro‐A‐117 and Gly‐A‐121 respectively (Figures 3 and S44).

As outlined in Figure 3, the comparison of the binding pockets of HNQ‐2, HNQ‐6 and HNQ‐7 to the reference inhibitor HQNO revealed that the designed set of ligands are binding into the similar pocket but with slight difference mode of interactions pattern with regard to the involved amino acids. Furthermore, the methylene‐bridge of all alkylated HNQ‐derivatives showed no interactions, which facilitate presumably to the flexibility of the compounds needed to enter the binding pocket. Indeed, HNQ‐11 lacks this methylene spacer and shows relatively lowest inhibitory potential of all tested HNQs. In addition, the key finding here suggesting that in all scaffolds Phe‐A‐146 is the major interaction partner with the hydroxynaphthoquinone ring. In addition, for the most selective cyt *bo*_3_ inhibitor HNQ‐2, an interesting set of π‐π interactions in between Phe‐A‐146 and the benzyl‐ring was observed. Moreover, this benzylic π‐electron cloud is also playing crucial role in interactions with Ile‐A‐120 and Pro‐A‐117, which creates space to accommodate the HNQ‐2 within the binding pocket of ubiquinol oxidase cyt *bo*_3_. These key findings from the molecular modelling studies are in good agreement with the enzyme inhibition assays.

Conceptually, we consider our results as an extension of the conclusion that has been shown in the study of Mogi et al., by which the ubiquinol oxidation sites in cytochromes cyt *bo_3_* and cyt *bd* were examined with modified Aurachin C analogues.^41^ It was demonstrated that an increase in the chain length of the alkyl chain decreases the inhibitory effect towards cyt *bo_3_* in contrast to cyt *bd,* suggesting a smaller binding site of cyt *bo_3_* compared to cyt *bd*. In the current study, we observed that alkylations of hydroxynaphthoquinone scaffold with alkyl chains or bulky substituents are positively influencing the inhibition of both, cyt *bd* and cyt *bo_3_ (e. g.,* HNQ‐1 and HNQ‐12). We further found that alkylation with cyclic residues like benzyl groups (HNQ‐2, HNQ‐6, HNQ‐7) increases the inhibitory potency only for cyt *bo_3_*.

To confirm the selectivity of designed alkylated HNQ derivatives for cyt *bo*_3_ oxidase we performed docking analyses with cyt *bd* oxidase. We used the atomic structure of the *bd‐*oxidase (PDB ID: 5DOQ) from *Geobacillus thermodenitrificans* 44 (determined by X‐ray diffraction at 3.05 Å). Based on this crystal‐structure (5DOQ) of the menaquinol oxidase we performed docking in the quinol binding loop.^45^ We observed that HQNO and Aurachin C show a higher affinity for cyt *bd* oxidase based on the binding energies compared to cyt *bo_3_* (see Supporting Information Table S5). In contrary, our set of tested HNQ‐derivatives is more selective for cyt *bo_3_* oxidase. Our *in silico* experiments are hence complementary to the biochemical data presented in this work. Detailed interactions of HQNO, Aurachin C, and HNQ‐derivatives are discussed in Supporting Information (Figures S48–S52). The long side chain seems to be the reason for the higher affinity of HQNO and Aurachin C1–10 towards the menaquinol binding domain of the cytochrome *bd* oxidase. By computational docking, it becomes obvious that the side chain is involved in alkyl and π‐alkyl‐type of interactions with Ile‐A‐146, Ile‐A‐22, Trp‐A‐374, Ile‐A‐370 and Val‐A‐187. The central scaffold of Aurachin C1‐10 is involved in a π‐σ‐type interaction with Ala‐A‐377 and a π‐π T‐shaped interaction with His‐A‐186.

In conclusion, this work extended the library of cyt *bo_3_* oxidase inhibitors with the beneficial naphthoquinone class. Twelve new 3‐alkylated hydroxynaphthoquinones derivatives were synthesized using L‐proline‐catalyzed three‐component reductive alkylation (TCRA). The successfully tested one‐step reaction was suitable to achieve a high alkylation efficiency in most cases. Furthermore, due to isolation of one intermediate HNQ‐11 the hypothesized alkylation mechanism was confirmed. The solubility of all synthesized derivatives was determined spectrophotometrically by a thermodynamic equilibrium assay in 96‐well plates to meet the requirements for functional enzyme tests. Hence in these inhibition experiments, this class shows a higher selectivity for *E. coli* cyt *bo*_3_ compared to previously studied inhibitors Aurachin C or HQNO. A purposed interaction mode with cyt *bo*_3_ was demonstrated. Simple benzylated HNQ‐2 is the most favorable cyt *bo*_3_ inhibitor regarding selectivity and efficacy within our library. Furthermore, combination of enzyme activity assay and In‐depth docking analysis provided detailed insights into the binding site of new alkylated HNQ derivatives, the positive role of the methylene bridge as well as characteristic of substituents to improve interactions with the amino acids within the binding pocket of targeted enzyme.

## Experimental Section

**General Procedure: Synthesis of 3‐alkylated hydroxynaphthoquinone derivatives (HNQ1‐12) by TCRA**: A mixture of 2‐hydroxy‐1, 4‐naphthoquinone (1 equiv), Hantzsch ester (1 equiv), l‐proline (0.5 equiv) and corresponding aldehyde (2 equiv) dissolved in dried CH_2_Cl_2_ or MeOH was heated at reflux under N_2_ for 18 h. After cooling to room temperature, the obtained solution was concentrated in vacuum and the alkylated product was purified by silica‐gel column chromatography. In some cases, the purification was impracticable in the presence of oxidized Hantzsch ester exhibiting identical *R*_f_ value to those of desired product. Therefore, a mixture of methanol/water (3 : 1) was added to the mixed fraction (after HPLC purification). The precipitated solid was filtrated out and LiOH (3–5 equiv) was added (reaction was controlled by TLC). The solution was than stirred for 2 h and Methanol was removed. The aqueous layer was covered with CH_2_Cl_2_ and acidified with a saturated NaHCO_3_ solution. The two layers were separated and the aqueous layer was further extracted with CH_2_Cl_2_. The organic layers were combined and the solvent was evaporated under reduced pressure to yield finally after the saponification the pure product.

**2‐Hydroxy‐3‐tridecyl‐1,4‐naphthoquinone (HNQ‐1)**: Aldehyde: tridecanal; reaction solvent: CH_2_Cl_2_; column chromatography with *c*‐hexane/EtOAc (4 : 1); yellow solid (65 %); *R*_f_=0.70 (*c*‐hexane/EtOAc 1 : 1); ^1^H NMR (600 MHz, CDCl_3_): *δ* 8.12(d, ^*3*^ *J*=7.9 Hz, 1.0 Hz, 1H), 8.07 (dd, ^3^ *J*=7.6 Hz, ^4^ *J*=1.1 Hz, 1H), 7.75 (td, ^3^ *J*=7.7 Hz, ^4^ *J*=1.5 Hz, 1H), 7.67 (td, ^3^ *J*=7.7 Hz, ^4^ *J*=1.1, 1H), 7.30 (br s, 1H, OH), 2.59 (t, ^3^ *J*=7.7 Hz, 2H), 1.53–1.25 (m, 22H), 0.87 (t, ^3^ *J*=6.9 Hz, 3H) ppm; ^13^C NMR (150 MHz, CDCl_3_): *δ*=184.8, 181.6, 153.2, 135.0, 133.1, 132.9, 129.6, 127.0, 126.2, 125.0, 32.0, 29.9, 29.8, 29.7, 29.6, 29.5, 28.4, 23.6, 22.8, 14.3 ppm; ESI‐MS (*m/z*): calcd for C_23_H_32_O_3_: 355.24 [M−H]^−^; found 355.27.

**2‐Hydroxy‐3‐methylene‐(benzyl)‐1,4‐naphthoquinone (HNQ‐2)**: Aldehyde: benzaldehyde; reaction solvent: CH_2_Cl_2_; column chromatography with *c*‐hexane/EtOAc (5 : 1); yellow solid (55 %); *R*_f_=0.45 (*c*‐hexane/EtOAc, 5 : 1); ^1^H NMR (600 MHz, CDCl_3_): *δ* 8.04 (dd, ^3^ *J*=7.6 Hz, ^4^ *J*=0.7 Hz, 1H), 7.98 (dd, ^3^ *J*=7.5 Hz, ^4^ *J*=0.9 Hz,1H), 7.66 (td, ^3^ *J*=7.5 Hz, ^4^ *J*=1.2 Hz, 1H), 7.59 (td, ^3^ *J*=7.6 Hz, ^4^ *J*=1.2 Hz, 1H), 7.37 (br s, 1H, OH), 7.32 (d, ^3^ *J*=7.4 Hz, 2H), 7.18 (t, ^3^ *J*=7.4 Hz, 2H), 7.10 (t, ^3^ *J*=7.2 Hz, 1H), 3.87 (s, 2H) ppm; ^13^C NMR (150 MHz, CDCl_3_): *δ*=183.2, 180.7, 152.1, 137.9, 134.0, 132.0, 131.8, 128.4, 128.2, 127.4, 125.9, 125.3, 125.1, 122.0, 28.2 ppm; ESI‐MS (*m/z*): calcd for C_17_H_12_O_3_: 263.08 [M−H]^−^; found 263.09.

**2‐Hydroxy‐3‐methylen‐(4‐*N*‐pyridyl)‐1,4‐naphthoquinone (HNQ‐3)**: Aldehyde: 4‐pyridincarboxyaldehyde; reaction solvent: CH_2_Cl_2_; column chromatography with *c*‐hexane/EtOAc (1 : 1); orange solid (55 %); *R*_f_=0.3 (*c*‐hexane/EtOAc, 4 : 1). ^1^H NMR (600 MHz, CDCl_3_): *δ* 8.49 (d, ^3^ *J*=5.9 Hz, 2H), 8.1 (dd, ^3^ *J*=7.6 Hz, ^4^ *J*=0.7 Hz, 1H), 8.0 (dd, ^3^ *J*=7.5 Hz, ^4^ *J*=0.9 Hz, 1H) 7.90 (td, ^*3*^ *J*=7.7 Hz, ^*4*^ *J*=1.2 Hz, 2H) 7.85 (td, ^*3*^ *J*=7.7 Hz, ^*4*^ *J*=1.2 Hz, 2H), 7.34 (d, ^3^ *J*=5.9, 2H), 3.90 (s, 2H) ppm; ^13^C NMR (150 MHz, [D_6_]DMSO): *δ*=183.9, 181.0, 156.9, 149.0, 134.5, 133.2, 131.9, 130.2, 125.7, 124.0, 120.3, 28.1 ppm; ESI‐MS (*m/z*): calcd for C_16_H_11_NO_3_: 264.07 [M−H]^−^; found 264.09.

**2‐Hydroxy‐3‐methylen‐(4‐*N***,***N*** **‐dimethylanilid)‐1,4 naphthoquinone (HNQ‐4)**: Aldehyde: 4‐*N*,*N*‐dimethylaminobenzaldehyde; reaction solvent: CH_2_Cl_2_; column chromatography with *c*‐hexane/EtOAc (2 : 1); orange solid (28.4 %); *R*_f_=0.4 (*c*‐hexane/EtOAc 4 : 1). ^1^H NMR (600 MHz, CDCl_3_): *δ* 8.01 (d, ^3^ *J*=7.2 Hz, 1H), 7.95 (d, ^3^ *J*=7.4 Hz, 1H), 7.63 (m, 1H), 7.56 (m, 1H), 7.2 (d, ^3^ *J*=8.6 Hz, 2H), 6.58 (d, ^3^ *J*=8.2 Hz, 2H), 3.77 (s, 2H), 2.79 (s, 6H) ppm; ^13^C NMR (150 MHz, CDCl_3_): *δ*=183.5, 180.8, 151.7, 148.2, 133.8, 131.8, 128.8, 128.4, 125.8, 125.0, 122.7, 111.9, 39.8, 27.1 ppm; ESI‐MS (*m/z*) calcd for C_19_H_17_NO_3_: 306.12 [M−H]^−^; found 306.13.

**2‐Hydroxy‐3‐[(indazol‐5‐yl)methyl]‐1,4‐naphthoquinone (HNQ‐5)**: Aldehyde: indazol‐5‐carboxyaldehyde; reaction solvent: CH_2_Cl_2_; column chromatography with *c*‐hexane/EtOAc (2 : 1); orange solid (66 %); *R*_f_=0.16 (*c*‐hexane/EtOAc 2 : 1). ^1^H NMR (600 MHz, [D_6_]DMSO): *δ* 12.9 (br,1H), 8.0–7.9 (m, 3H), 7.8 (td, ^*3*^ *J*=7.9 Hz, ^*4*^ *J*=1.1 Hz, 1H), 7.8 (td, ^*3*^ *J*=7.7 Hz, ^*4*^ *J*=1.0 Hz, 1H), 7.6 (s, 1H), 7.42 (d, *J=8.9 Hz,1H), 7*.3 (dd, ^*3*^ *J*=8.5 Hz, ^*4*^ *J*=1.2 Hz, 1H), 3.9 (s, 2H) ppm; ^13^C NMR (150 MHz, CDCl_3_): *δ*=184.3, 181.2, 155.7, 134.5, 133.2, 131.9, 131.2, 130.1, 127.4, 125.8, 125.7, 123.0, 122.9, 119.3, 109.8, 31.1, 29.9, 28.4 ppm; ESI‐MS (*m/z*): calcd for C_18_H_12_N_2_O_3_: 303.08 [M−H]^−^; found 303.11.

**2‐Hydroxy‐3‐[(1,2‐methylenedioxybenz‐5 yl)methyl]‐1,4‐naphthoquinone (HNQ‐6)**: Aldehyde: piperonal; reaction solvent: CH_2_Cl_2_; column chromatography with *c*‐hexane/EtOAc (2 : 1); orange solid (80 %); *R*_f_=0.37 (*c*‐hexane/EtOAc 2 : 1); ^1^H NMR (600 MHz, CDCl_3_): *δ* 8.04 (d, ^3^ *J*=7.6 Hz, 1H), 7.98 (d, ^3^ *J*=7.4 Hz, 1H), 7.67 td, ^3^ *J*=7.5 Hz, 1H), 7.60 (td, ^3^ *J*=7.5 Hz, 1H), 6.82 (br, 1H), 6.78 (d, ^3^ *J*=8.2 Hz, 1H), 6.62 (d, ^3^ *J*=8.0 Hz, 1H), 5.81 (s, 2H), 3.78 (s, 2H) ppm; ^13^C NMR (150 MHz, CDCl_3_): *δ*=184.4, 181.7, 152.9, 147.5, 146.0, 135.1, 133.0, 132.8, 132.6, 129.4, 126.9, 126.1, 123.1, 122.1, 109.8, 108.2, 100.8, 28.9 ppm; ESI‐MS (*m/z*) calcd for C_18_H_12_O_5_, 307.07 [M−H]^−^, found 307.08.

**2‐Hydroxy‐3‐[(4‐hydroxy‐3‐methoxyphenyl)methyl]‐1,4‐naphthoquinone (HNQ‐7)**: Aldehyde: vanillin; reaction solvent: CH_2_Cl_2_; column chromatography with *c*‐hexane/EtOAc (2 : 1); yellow solid (37 %); *R*_f_=0.16 (*c*‐hexane/EtOAc 2 : 1); ^1^H NMR (600 MHz, [D_6_]DMSO): *δ* 8.7 (br. s, 1H), 8.0 (dd, ^*3*^ *J*=7.7 Hz, ^*4*^ *J*=0.9 Hz, 1H); 7.97(dd, ^*3*^ *J*=7.7 Hz, ^*4*^ *J*=1.4 Hz, 1H), 7.82 (td, ^*3*^ *J*=7.6 Hz, ^*4*^ *J*=1.5 Hz, 1H); 7.77 (td, ^*3*^ *J*=7.5 Hz, ^*4*^ *J*=1.5 Hz, 1H); 6.85 (s, 1H); 6.64 (m, 2H); 3.73 (s, 3H) ppm; ^13^C NMR (150 MHz, [D_6_]DMSO): *δ*=181.8, 178.7, 153.0, 144.8, 142.2, 132.0, 130.7, 129.5, 127.7, 127.5, 123.3, 123.2, 120.5, 118.3, 112.8, 110.6, 53.2, 25.5 ppm; ESI‐MS (*m/z*) calcd for C_18_H_14_O_5_: 309.08 [M−H]^−^; found 309.12.

**2‐Hydroxy‐3‐[(1H‐pyrrolo(2,3‐b)pyridine‐3‐yl)methyl]‐1,4‐naphthoquinone (HNQ‐8)**: Aldehyde: 7‐azaindol‐3‐carboxy aldehyde; reaction solvent: CH_2_Cl_2_; column chromatography with *c*‐hexane/EtOAc (2 : 1); orange solid (51 %). R_*f*_=0.23 (*c*‐hexane/EtOAc 1 : 7); ^1^H NMR (600 MHz, [D_6_]DMSO): *δ* 11.34 (s, 1H), 8.16 (dd, ^*3*^ *J*=4.7 Hz, ^*4*^ *J*=1.4 Hz, 1H), 8.04 (dd, ^*3*^ *J*=7.8 Hz, ^*4*^ *J*=1.2 Hz, 1H), 7.97 (dd, ^*3*^ *J*=7.5 Hz, ^*4*^ *J*=1.1 Hz, 2H); 7.80 (td, ^3^ *J*=7.4 Hz, ^4^ *J*=1.4 Hz, 1H), 7.74 (td, ^3^ *J*=7.5 Hz, ^4^J=1.3 Hz, 1H), 7.23 (d, ^3^ *J*=1.8 Hz, 1H), 7.03 (dd, ^3^ *J*=7.9 Hz, 4.7 Hz, 1H), 3.9 (s, 2H) ppm; ^13^C NMR (150 MHz, [D_6_]DMSO): *δ*=184.2, 181.2, 155.5, 148.3, 142.2, 134.4, 133.0, 131.9, 130.1, 126.7, 125.7, 125.6, 123.8, 122.5, 119.2, 114.8, 110.6, 18.7 ppm; ESI‐MS (*m/z*): calcd for C_18_H_12_N_2_O_3_: 305.08 [M−H]^+^; found 305.16.

**2‐Hydroxy‐3‐[(ferreocenyl)methyl]‐1,4‐naphthoquinone (HNQ‐9)**: Aldehyde: ferrocenecarboxaldehyde; reaction solvent: CH_2_Cl_2_; column chromatography with CH_2_Cl_2_/MeOH (49 : 1); green solid (22 %); *R*_f_=0.25 (CH_2_Cl_2_/MeOH 49 : 1); ^1^H NMR (600 MHz, CDCl_3_): *δ* 8.02 (d, ^*3*^ *J*=7.5 Hz, 1H), 7.95 (d, ^*3*^ *J*=7.1 Hz, 1H) 7.64–7.56 (m, 2H), 7.32 (br. s, 7.32), 4.08 (m, 9H), 3.97 (s, 1H), 3.57 (s, 2H) ppm; ^13^C NMR (150 MHz, CDCl_3_): *δ*=184.5, 181.9, 152.7, 135.0, 133.0, 129.5, 127.0, 126.1, 123.5, 85.8, 69.1, 68.8, 67.4, 23.2 ppm; ESI‐MS (*m/z*) calcd for C_23_H_22_FeO_3_: 372.03 [M−H]^−^; found 371.05.

**2‐Hydroxy‐3‐[(3,5‐dihydroxybenzyl)methyl]‐1,4‐naphthoquinone (HNQ‐10)**: Aldehyde: 3,5‐dihydroxybenzaldehyde; reaction solvent: MeOH; column chromatography with CH_2_Cl_2_/acetone (49 : 1); red solid (66 %); *R*_f_=0.56 (CH_2_Cl_2_/MeOH 3 : 1); ^1^H NMR (600 MHz, [D_6_]DMSO): *δ* 8.90 (br. s, 2H), 7.91–7.70 (m, 2H), 7.76 (m, 1H), 7.70 (m, 1H), 6.16 (s, 2H), 5.96 (s, 1H), 3.18 (s, 2H) ppm; ^13^C NMR (150 MHz, MeOD): *δ*=185.3; 159.0; 145.4; 136.07; 132.7; 127.0; 122.0; 115.6; 108.4; 100.9; 29.8 ppm; ESI‐MS (*m/z*) calcd for C_17_H_12_O_5_ 295.07 [M−H]^−^; found 294.94.

**2‐Hydroxy‐3‐[(2‐thiazol(2‐3)benzyl)methyl)pyrrolidine‐2‐carboxylic acid‐1,4‐naphthoquinone (HNQ‐11)**: Aldehyde: benzothiazole‐2‐carboxaldehyde; reaction solvent: MeOH; column chromatography with CH_2_Cl_2_/MeOH (14 : 1); red solid (60 %); *R*_f_=0.55 (CH_2_Cl_2_/MeOH 14 : 1); ^1^H NMR (600 MHz, CDCl_3_): *δ* 7.99 (d, ^3^ *J*=7.9 Hz, 2H), 7.96 (d, ^3^ *J*=7.9 Hz, 1H), 7.80 (d, ^3^ *J*=8.1 Hz, 1H), 7.61 (td, ^3^ *J*=7.4 Hz, ^4^ *J*=1.3 Hz, 1H), 7.54 (td, ^3^ *J*=7.7 Hz, ^4^ *J*=1.3 Hz, 1H), 7.43 (t, ^3^ *J*=7.6 Hz, 1H), 7.34 (t, ^3^ *J*=7.6 Hz, 1H), 4.55 (t, ^3^ *J*=8.4 Hz, 1H), 4.47 (d, ^3^ *J*=14.7 Hz, 1H), 4.14 (d, ^3^ *J*=14.6 Hz, 1H) 3.55 (m, 1H), 2.94 (q, ^3^ *J*=9.7 Hz), 2.43 (m, 1H), 2.0–1.93 (m, 3H), ppm; ^13^C NMR (150 MHz, CDCl_3_): *δ*=183.1, 181.8, 163.1, 152.8, 135.7, 133.9, 132.8, 132.4, 131.5, 126.6, 126.4, 125.9, 125.8, 123.6, 121.8, 64.7, 54.9, 52.9, 31.6, 23.4 ppm; MALDI: calcd for C_23_H_18_N_2_O_5_S: 390.1 [M−CO_2_]^−^; found 390.7.

**2‐Hydroxy‐3‐[(4‐phenoxybenzyl)methyl]‐1,4‐naphthoquinone (HNQ‐12)**: Aldehyde: 3‐phenoxy‐benzaldhyde; reaction solvent: CH_2_Cl_2_; column chromatography with *c*‐hexane/EtOAc (3 : 1); orange solid (45 %); *R*_f_=0.57 (*c*‐hexane/EtOAc 3 : 1); ^1^H NMR (600 MHz, [D_6_]DMSO): *δ* 8.05 (dd, ^3^ *J*=7.7 Hz, ^4^ *J*=1.1 Hz, 1H), 8.0 (dd, ^3^ *J*=7.5 Hz, ^4^ *J*=1.1 Hz, 1H), 7.67 (td, ^3^ *J*=7.7 Hz, ^4^ *J*=1.4, 1H), 7.60 (td, ^3^ *J*=7.7 Hz, ^4^ *J*=1.1 Hz, 1H), 7.35 (s, 1H), 7.27 (d, ^3^ *J*=8.7 Hz, 2H), 7.21 (m, 2H), 6.98 (t, ^3^ *J*=7.3 Hz, 1H), 6.88 (dd, ^3^ *J*=8.7 Hz, ^4^ *J*=0.9 Hz, 2H), 6.82 (d, ^3^ *J*=8.5 Hz, 2H), 3.83 (s, 2H) ppm; ^13^C NMR (150 MHz, [D_6_]DMSO): *δ*=179.2, 176.5, 152.2, 150.4, 147.7, 129.8, 128.5, 127.8, 127.6, 125.3, 124.4, 124.2, 121.7, 121.0, 117.8, 113.7, 113.5, 23.1 ppm; ESI‐MS (*m/z*) calcd for C_23_H_16_O_4_: 355.1 [M−H]^−^; found 355.05.

Characterization data for all synthesized compounds can be found in the Supporting Information

**Solubility assay**: Thermodynamic equilibrium solubility of HNQ compounds was determined in sodium phosphate buffer containing 20 mM NaPi, 50 mM NaCl, 0.02 % DDM at pH 8.0. All steps were performed at room temperature following the method by Bharate and Vishwakarma.^36^ Measurements were done in triplicates. HNQ compounds were dissolved in a particular amount of methanol or CH_2_Cl_2_ (HNQ‐9) needed for complete dissolving. Required volumes of these methanol stock solutions were transferred in 1.5 mL reaction tubes (0.1–600 μL dependent on compound and stock concentration) to get a concentration row. After 24–48 h complete evaporation of methanol or CH_2_Cl_2_ was accomplished. Remaining solid compounds were dissolved by adding 100/200 μL of sodium phosphate buffer to the reaction tubes, which were shaken at 200 rpm for one day for equilibration. Next day, centrifugation of the reaction tubes for 30 min at 13 000 rpm at 11000 g. 50/100 μL of supernatant were pipetted into a 96‐well plate (UV‐Star, Greiner), and absorbance spectra were recorded for each well with a TECAN Spark plate reader (Tecan Trading AG, Switzerland). By plotting absorbance at *λ*_max_ against concentration [mM] of the compound, saturation point of graph indicates the thermodynamic equilibrium solubility. Data analysis and visualization with SigmaPlot Pro 12.5 (Systat Software GmbH, Germany).

**Oxygen reductase activity measurements**: Oxygen reductase activity was measured as oxygen consumption rate of purified protein by an OX‐MR Clark‐type oxygen electrode linked to a PA 2000 picoammeter and to an ADC‐216 for analog to digital conversion. Recording of data with SensorTrace Basic 2.1 software (all Unisense, Denmark).

Measurements were performed at room temperature in stirred 2 mL – glass vials with a total reaction volume of 600 μL. Oxygen consumption was initiated by adding 30 nM of the respective enzyme to the equilibrated mixture containing 20 mM NaPi (pH 8.0), 50 mM NaCl, 0.02 % DDM, 5 mM dithiothreitol (DTT) and 200 μM Ubiquinone‐1 (2,3‐dimethoxy‐5‐methyl‐6‐(3‐methylbut‐2‐en‐1‐yl)‐1,4‐benzoquinone). Equilibration was done for 10 min prior to enzyme addition. Inhibition experiments with 250 μM of respective HNQ from 20 mM stock solution in EtOH before equilibration. Data analysis and visualization with Origin Lab Pro 9.5 (Additive GmbH, Germany). HQNO (2‐heptyl‐4‐quinolinol 1‐oxide) was purchased from biomol GmbH, Hamburg. Determination of apparent *k*_i_ (*k*_i_^app^) was performed under the same conditions but with a range of inhibitor concentrations from 1000 μM to 0.2 μM. The *k*_i_^app^ value was adopted as the EC50 from the sigmoidal DoseResp Fit. Further information can be found in Figures S40–S43.

### Production of cytochrome *bd‐I* and *bo_3_* from *E. coli*

*Production of cytochrome* bd‐I *from* E. coli: Production of cytochrome *bd‐I* oxidase from *E. coli* in *E. coli* C43(DE3) Δ*bo*_3_ cells transformed with the pet17b‐*cydABX‐*StrepII plasmid.^46^ 1 mL of 50 % Glycerol stock was added to 50 mL LB‐Carb‐Kan (50 μg/mL carbenicillin; 50 mg/mL kanamycin) and incubated at 175 rpm at 37 °C for 8 h. Preculture was transferred to 1 L LB‐Carb‐Kan to grow over night. Production culture of 2.5 L LB‐Kan was inoculated with 70 mL of overnight culture supplemented with 0.025 mM IPTG to start basal production from the beginning. After reaching OD_600_ 0.7 heterologous *bd‐I* oxidase production was started by adding IPTG to a final concentration of 0.25 mM. Incubation at 37 °C for 4 h and lower temperature to 30 °C for another 16 h. Cell harvesting was carried out by centrifugation with Avanti J‐26 XP at 4 °C at 8000 *g*. Cell disruption via microfluidizer for six cycles at 80 psi in 50 mM NaPi (pH 8.0) and 100 mM NaCl supplemented with 1 mM MgCl, recombinant DNase I (Sigma) and protease inhibitor Aminoethyl‐benzene‐sulfonyl fluoride (Pefabloc, Roche). Low‐velocity centrifugation at 5000 *g* at 4 °C for 30 min before high‐velocity centrifugation of the supernatant at 220 000 *g* at 4 °C for 90 min. Membrane pellets were resuspended in 50 mM NaPi (pH 8.0), 100 mM NaCl containing buffer and stored at −80 °C.

*Streptactin purification of* E. coli bd‐I *oxidase*: Solubilization of isolated membranes in 50 mM NaPi (pH 8.0), 100 mM NaCl with 1 % *n*‐dodecyl β‐d‐maltoside (β‐DDM) to the mass ratio of 1 : 5 detergent/membrane protein at 4 °C for 120 min on orbital shaker. Removal of unsolubilized material by 70 000 *g* for 30 min. The supernatant was filtrated through a 0.2 μm syringe filter and Avidin was added to a final concentration of 2 mg/mL. Affinity chromatography was done via peristaltic pump with prepacked 5 mL StrepTrap HP column (GE Healthcare) equilibrated with 20 mM NaPi (pH 8.0), 100 mM NaCl, 0.02 % DDM at a flow rate of 3 mL/min. Washing step was carried out with the same buffer for 8 CV. Elution with the same buffer containing 10 mM desthiobiotin (IBA Lifesciences). Sample polishing by dialysis with Slide‐A‐Lyzer (CutOff 10 K) dialysis cassettes (Thermo Fisher Scientific) in 4 L 50 mM NaPi (pH 8.0), 100 mM NaCl with 1 % β‐DDM overnight. Verification of *E. coli bd‐I‐type* oxidase and purity of the product was realized by SDS‐page and native page gel electrophoresis, see the Supporting information for results.

*Production of cytochrome* bo_3_ *from* E. coli: The protocol for the *bo_3_* – oxidase production from ref. [39] was used. In brief strain GO195 transformed with pIRHisA plasmid was a kind gift from Bob Gennis Lab. The purified membranes were a kind gift from Hao Xie.

From an overnight pre‐culture in 50 mL LB‐Amp‐Kan (100 μg/mL ampicillin; 50 μg/mL kanamycin) 10 mL was transferred into 2.5 L LB medium supplemented with 3 % Lactic acid and 500 μM CuSO_4_. Harvest in mid‐logarithmic phase. Membrane preparation via two French Press cycles at 20 000 psi in 50 mM KPi (pH 8.3), 5 mM MgSO_4_ with 4 mg/mL DNAse and Pefabloc. Followed by 15 min centrifugation at 17 000 *g* and high‐spin centrifugation of the supernatant at 180 000 *g* for 3 h. Membranes were stored at ‐80 °C prior to Ni‐NTA affinity purification.

*Ni‐NTA purification of* E. coli bo_3_ *oxidase*: A protocol slightly modified from ref. [39] as used for cytochrome *bo_3_*‐oxidase. Solubilization of isolated membranes in 50 mM NaPi with 1 % Triton X‐100 and 1.25 % octylglucoside at 4 °C for 1 h on orbital shaker. Removal of unsolubilized material by 70 000 *g* for 30 min. 4 mL Ni‐NTA bed were equilibrated with 50 mM NaPi (pH 8.3), 0.1 % Triton X‐100 and 25 mM Imidazole before solubilized protein was added. Washing step with 4 CV of the equilibration buffer, followed by high salt washing step with 250 mM NaPi (pH 8.3), 0.1 % Triton X‐100 and 25 mM Imidazole for 4 CV. Third washing with 50 mM NaPi (pH 8.3), 0.1 % DDM and 25 mM Imidazole for 4 CV to change the detergent from Triton X‐100 to DDM and lower the salt concentration. For elution a linear gradient of imidazole up to 300 mM was used. His‐tagged *bo_3_* oxidase eluted between 100 mM and 200 mM. The pooled fractions were concentrated using a Amicon ultra concentrator with 50 kDa cutoff (Millipore) and dialyzed overnight with Slide‐A‐Lyzer (CutOff 10 K) dialysis cassettes (Thermo Fisher Scientific) in 4 L 50 mM NaPi (pH 8.0), 100 mM NaCl with 1 % β‐DDM. Verification of *E. coli bo_3_* oxidase and purity of the product was realized by SDS‐page and native page gel electrophoresis, see the Supporting information for results.

*Molecular docking procedure*: The geometries of all the scaffolds were optimized by using the Gaussian 09 semi‐empirical PM3 force‐field method (Figure S44).^47^ Crystal structures (PDB IDs: 1FFT and 5DOQ) were obtained from the RSCB Protein Data Bank.^21^ Docking studies were performed by using Autodock 4.2 software.^48^ The visualization and analysis of interactions were performed by using PyMOL, version 0.99.^49^

## Conflict of interest

The authors declare no conflict of interest.

## Supporting information

As a service to our authors and readers, this journal provides supporting information supplied by the authors. Such materials are peer reviewed and may be re‐organized for online delivery, but are not copy‐edited or typeset. Technical support issues arising from supporting information (other than missing files) should be addressed to the authors.

Supplementary
