# Supplementary material for: Synthesis and Biological Screening of New Lawson Derivatives as Selective Substrate‐Based Inhibitors of Cytochrome bo3 Ubiquinol Oxidase from Escherichia coli
Source: ChemMedChem. 2020 Apr 14;15(14):1262–71. doi: 10.1002/cmdc.201900707 (PMC7497249; doi:10.1002/cmdc.201900707)
Supplement: Supplementary file 1 — Supplementary [file CMDC-15-1262-s001.pdf]

ChemMedChem

Supporting Information

Synthesis and Biological Screening of New Lawson Derivatives as Selective Substrate-Based Inhibitors of Cytochrome *bo*$_3$ Ubiquinol Oxidase from *Escherichia coli*

Isam Elamri[+], Melanie Radloff[+], Katharina F. Hohmann, Vijaykumar D. Nimbarte, Hamid R. Nasiri, Michael Bolte, Schara Safarian, Hartmut Michel, and Harald Schwalbe*© 2020 The Authors. Published by Wiley-VCH Verlag GmbH & Co. KGaA.
This is an open access article under the terms of the Creative Commons Attribution License, which permits use, distribution and reproduction in any medium, provided the original work is properly cited.

Supporting Information

Contents

1. X-ray structure of 2-Hydroxy-3-[(1,2-methylenedioxybenz-5yl)methyl]-1,4-naphtoquinone (HNQ-6).. 2
2. Characterization data of 3- alkylated hydroxynaphthoquinone derivatives 3
3. Solubility profiles of HNQ-s 21
4. Stock and assayed concentrations of HNQs 22
5. Simulated physicochemical properties of tested compounds 22
6. Purification of *E. coli* respiratory cytochrome bo_3 and *bd-I* oxidases 23
7. Inhibitory assay profiles of HNQ-2 and HNQ-12..24
8. Determination of apparent Ki of HNQ-2, HNQ-12 and HQNO……………………..25
9. 2D-interactions of HNQ-2,-6,-7 and HNQN in binding site of cyt bo_3 27
10. 2-Dimensional structures simulation by Gaussian semi-empirical PM3...................28
11. Binding energies of tested HNQ-compounds on cyt bo_3...29
12. Substrate binding site for ubiquinol-8 in cyt bo_3...29
13. Binding energies of tested HNQ-compounds on *cyt bd*………………………..……*30*
14. Interactions of HQNO and HNQ-derivatives with *cyt bd* oxidase (5DOQ)…………..32

1. X-ray structure of 2-Hydroxy-3-[(1,2-methylenedioxybenz-5yl)methyl]-1,4-naphtoquinone (HNQ-6)

a)

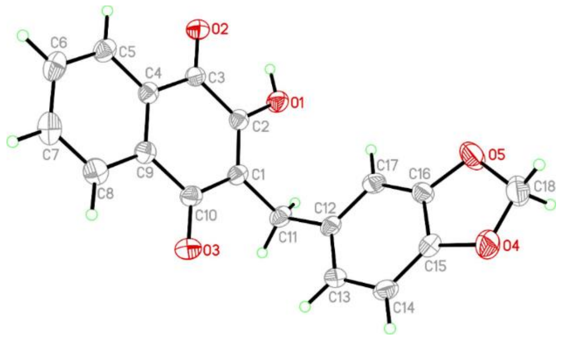

b)

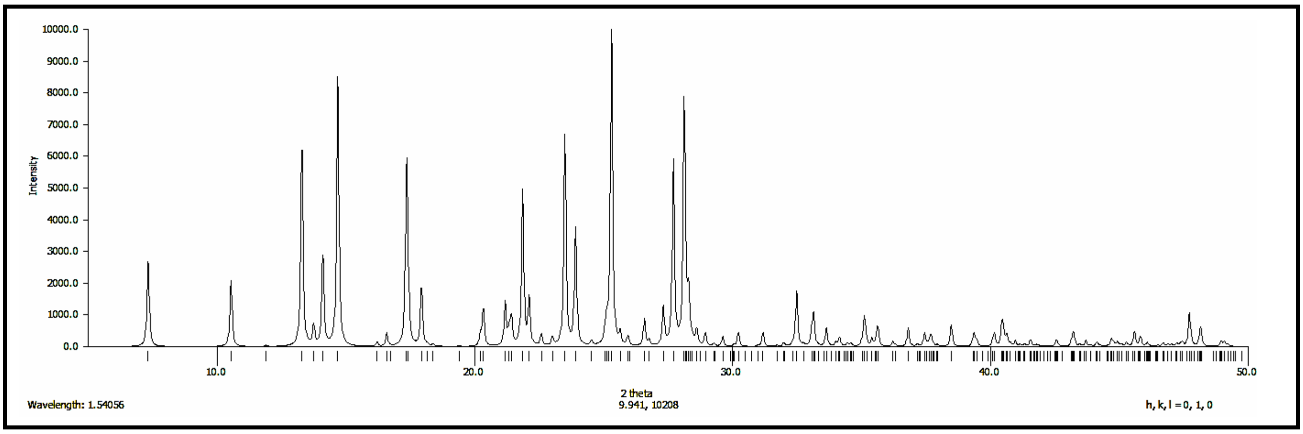

Figure S1. a) Perspective view of 2-Hydroxy-3-[(1,2-methylenedioxybenz-5yl)methyl]-1,4-naphtoquinone HNQ-6 determined by single crystal structure determination. The displacement ellipsoids at 50% probability. b) Powder diffraction diagram of HNQ-6.

Single crystal structure determination. Data were collected on a STOE IPDS II two-circle diffractometer with a Genix Microfocus tube with mirror optics using MoK_{α} radiation (λ = 0.71073 Å). The data were scaled using the frame scaling procedure in the *X-AREA* program system (Stoe & Cie, 2002). The structure was solved by direct methods using the program *SHELXS* (Sheldrick, 2008) and refined against F^2 with full-matrix least-squares techniques using the program *SHELXL* (Sheldrick, 2008). The H atom bonded to O was freely refined. In the crystal, the molecules are connected to centrosymmetric dimers via O-H···O hydrogen bonds.

Stoe & Cie, *X-AREA*. Diffractometer control program system. Stoe & Cie, Darmstadt, Germany, 2002.
G. M. Sheldrick, *Acta Crystallogr. Sect. A*, 2008, **64**, 112−122.

CCDC reference number: 1966615

Crystal data and structure refinement for HNQ-6.

Empirical formula	$C_{18}H_{12}O_5$	
Formula weight	308.28 g/mol	
Temperature	173(2) K	
Crystal system	Triclinic	
Space group	*P* -1	
Unit cell dimensions	a = 6.7480(8) Å	α= 98.489(9)°.
	b = 8.4863(9) Å	β= 99.027(9)°.
	c = 12.3520(14) Å	γ = 91.393(9)°.
Volume	690.11(14) $Å^3$	
Z	2	
Density (calculated)	1.484 Mg/m^3	
Absorption coefficient	0.109 mm^{-1}	
F(000)	320	
Crystal size	0.27 x 0.11 x 0.09 mm^3	
Theta range for data collection	3.729 to 27.517°.	
Index ranges	-8<=h<=8, -10<=k<=11, -15<=l<=15	
Reflections collected	7584	
Independent reflections	3119 [*R*(int) = 0.0203]	
Completeness to theta = 25.000°	98.5 %	
Absorption correction	Semi-empirical from equivalents	
Max. and min. transmission	1.000 and 0.681	
Refinement method	Full-matrix least-squares on F^2	
Data / restraints / parameters	3119 / 0 / 212	
Goodness-of-fit on F^2	1.073	
Final *R* indices [*I*>2sigma(*I*)]	*R*1 = 0.0416, *wR*2 = 0.1106	
R indices (all data)	*R*1 = 0.0475, *wR*2 = 0.1141	
Largest diff. peak and hole	0.280 and -0.168 e.$Å^{-3}$	

2. Characterization data of 3- alkylated hydroxynaphthoquinone derivatives

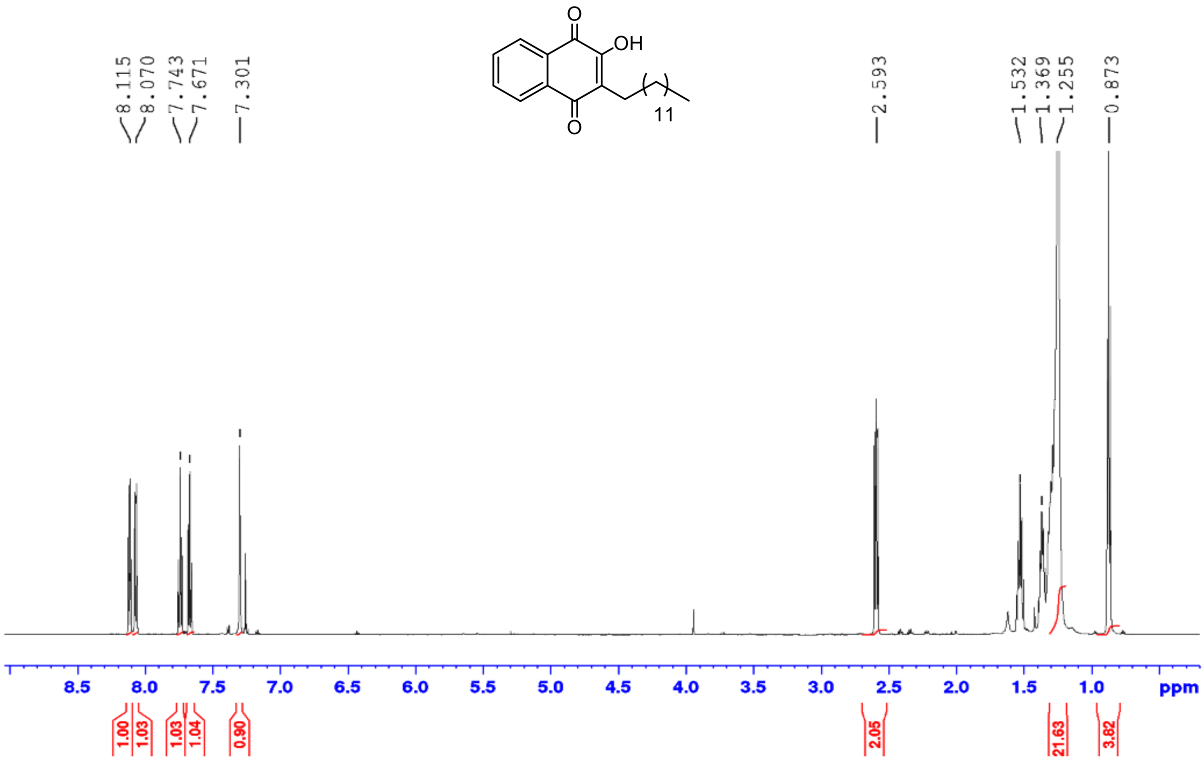

Figure S2. ^{1}H-NMR (600 MHz, $CDCl_3$) of 2-Hydroxy-3-tridecyl-1,4-naphtoquinone (HNQ-1).

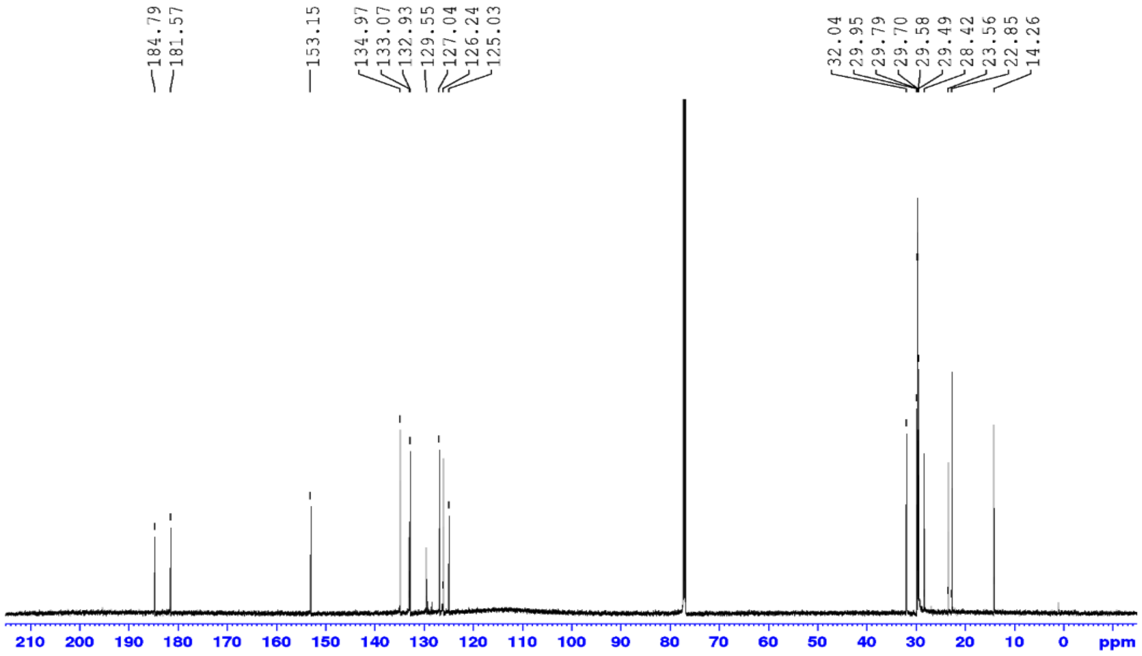

Figure S3. ^{13}C-NMR (150 MHz, $CDCl_3$) of 2-Hydroxy-3-tridecyl-1,4-naphtoquinone (HNQ-1).

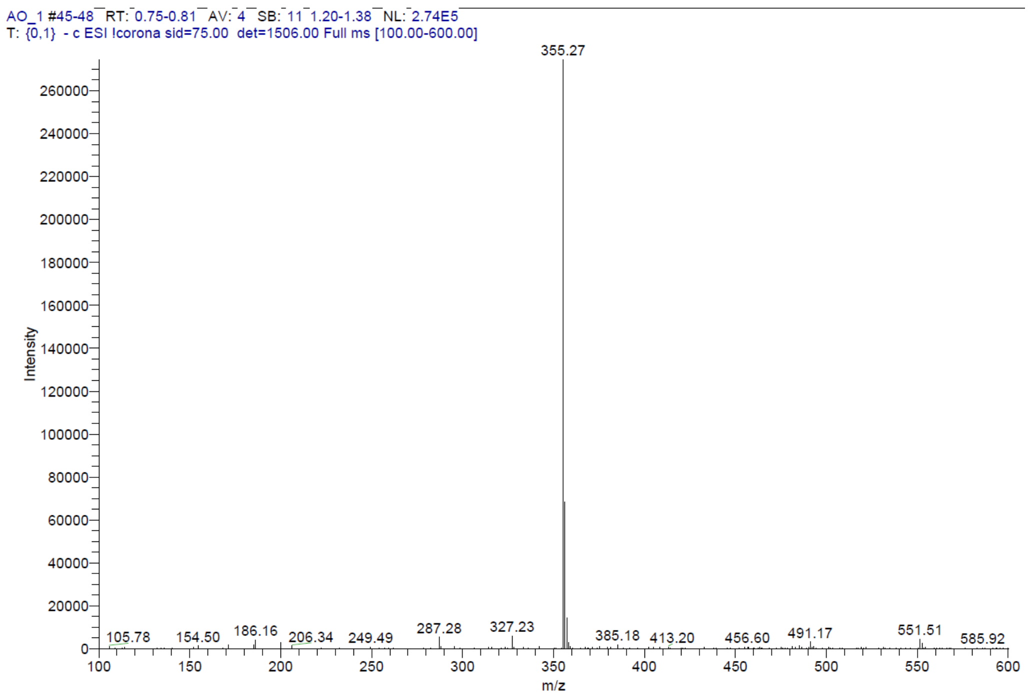

Figure S4. Mass (ESI^+) of 2-Hydroxy-3-tridecyl-1,4-naphtoquinone (HNQ-1).

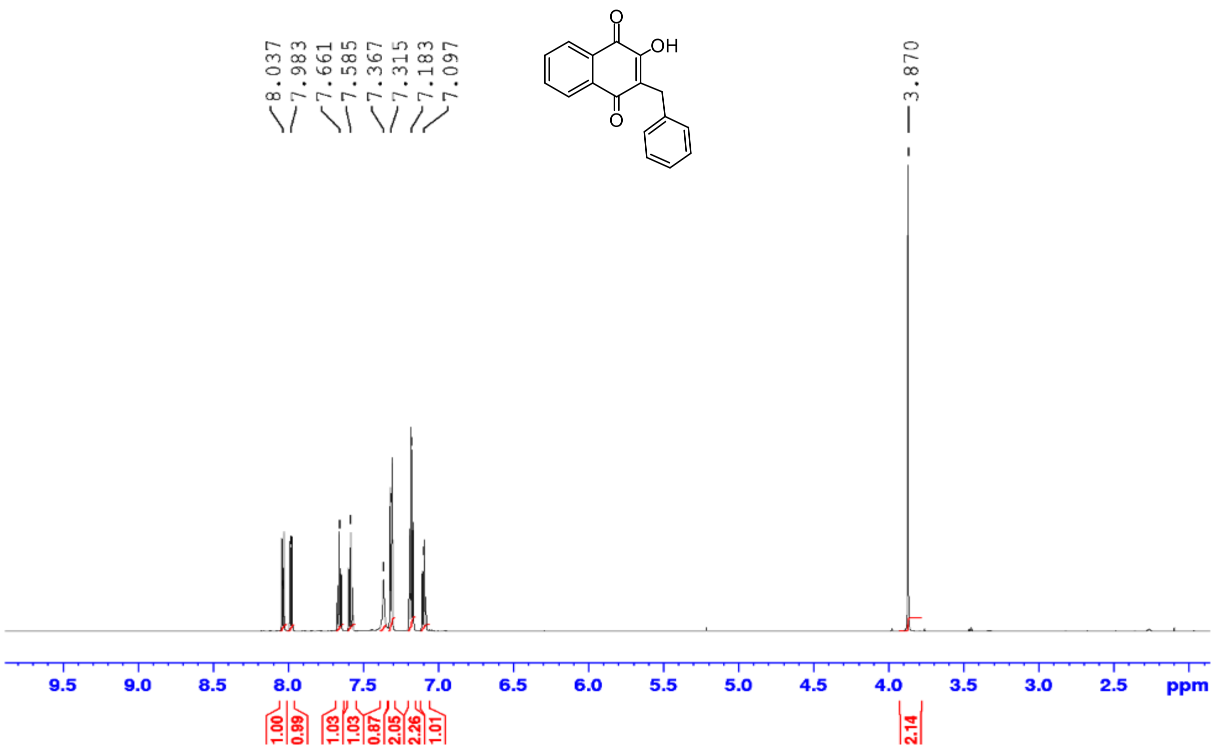

Figure S5. ^{1}H-NMR (600 MHz, $CDCl_3$) of 2-Hydroxy-3 -methylen-(benzyl)-1,4-naphtoquinone (HNQ-2).

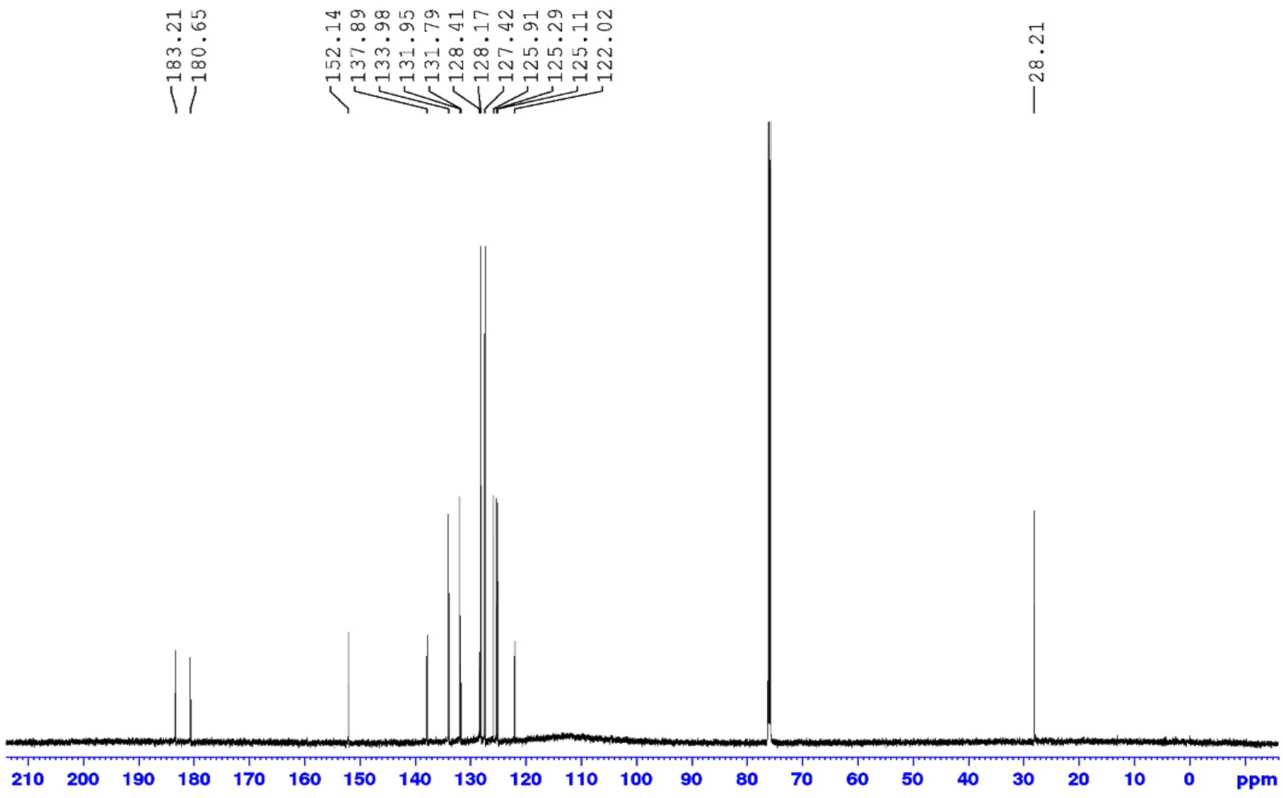

Figure S6. ^{13}C-NMR (150 MHz, $CDCl_3$) of 2-Hydroxy-3 -methylen-(benzyl)-1,4-naphtoquinone (HNQ-2).

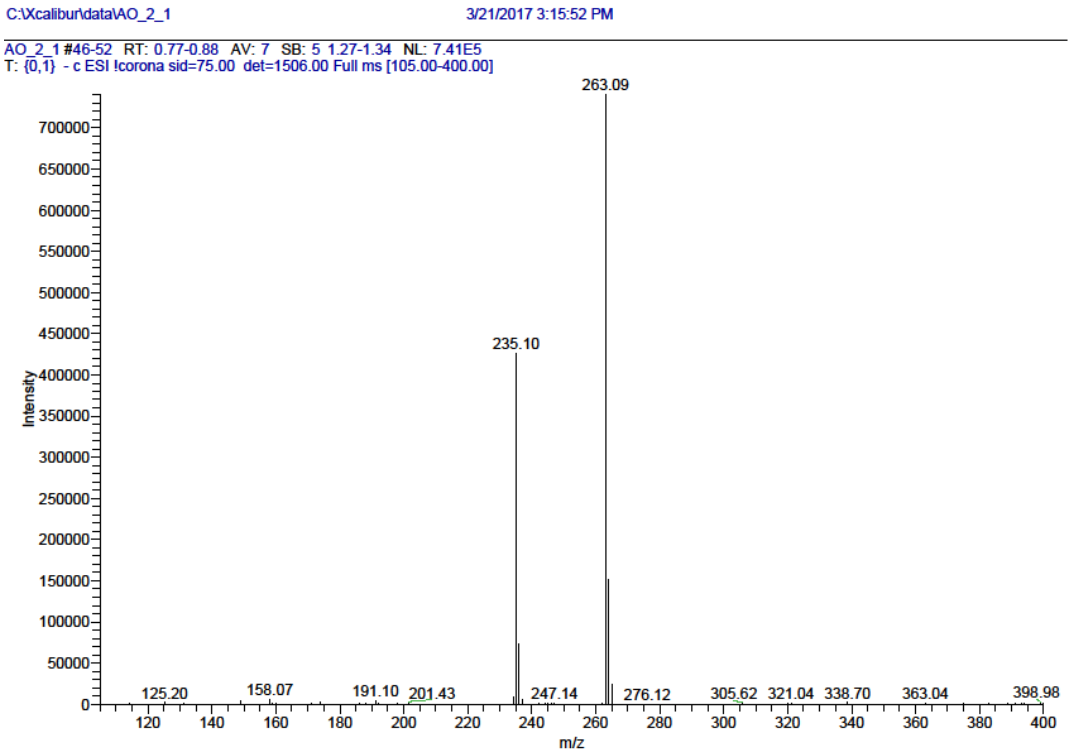

Figure S7. Mass (ESI^+) of 2-Hydroxy-3 -methylen-(benzyl)-1,4-naphtoquinone (HNQ-2).

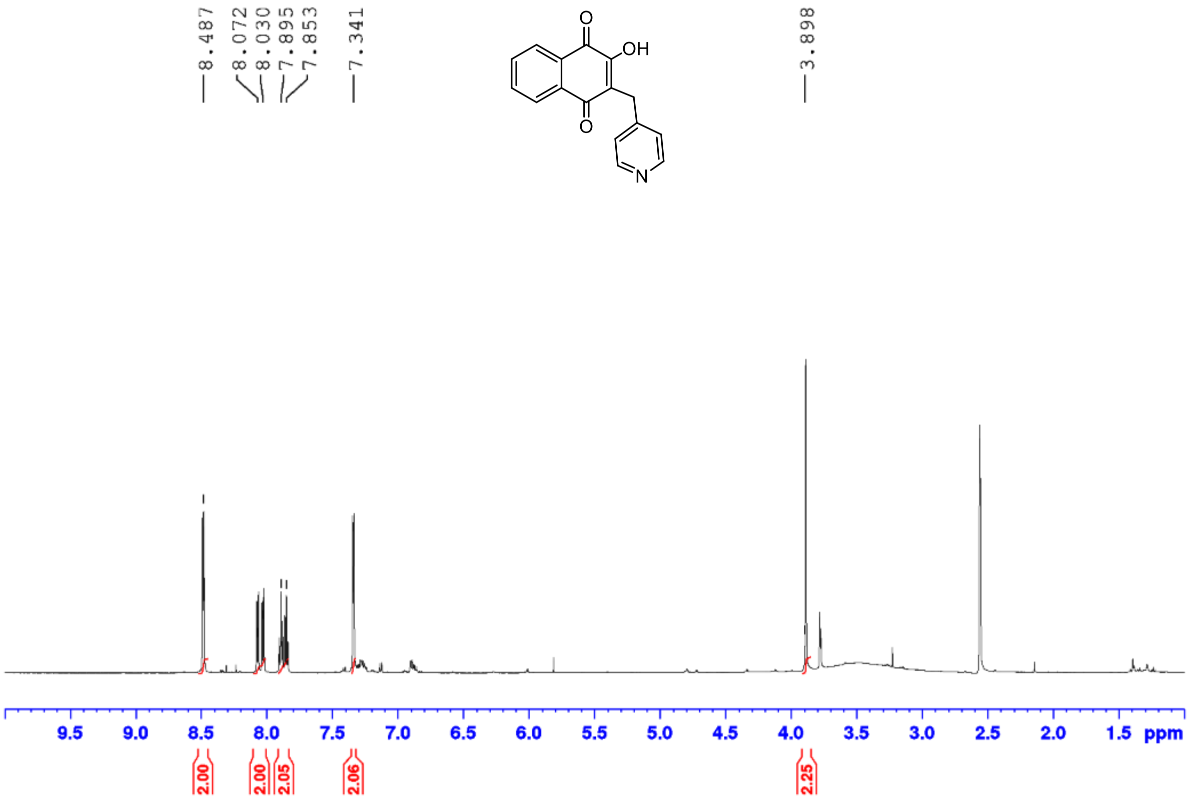

Figure S8. ^{1}H-NMR (600 MHz, $CDCl_3$) of 2-Hydroxy-3-methylen-(4-N-pyridyl)-1,4-naphtochinon (HNQ-3).

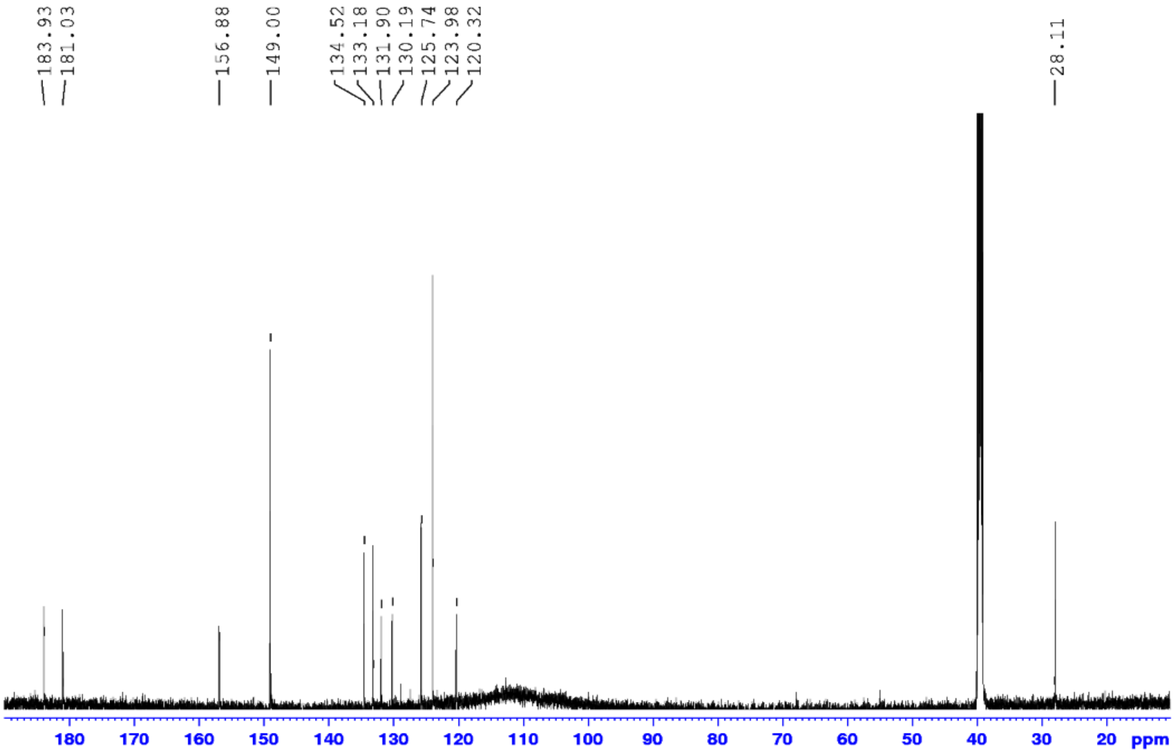

Figure S9. ^{13}C-NMR (150 MHz, $CDCl_3$) of 2-Hydroxy-3-methylen-(4-N-pyridyl)-1,4-naphtochinon (HNQ-3).

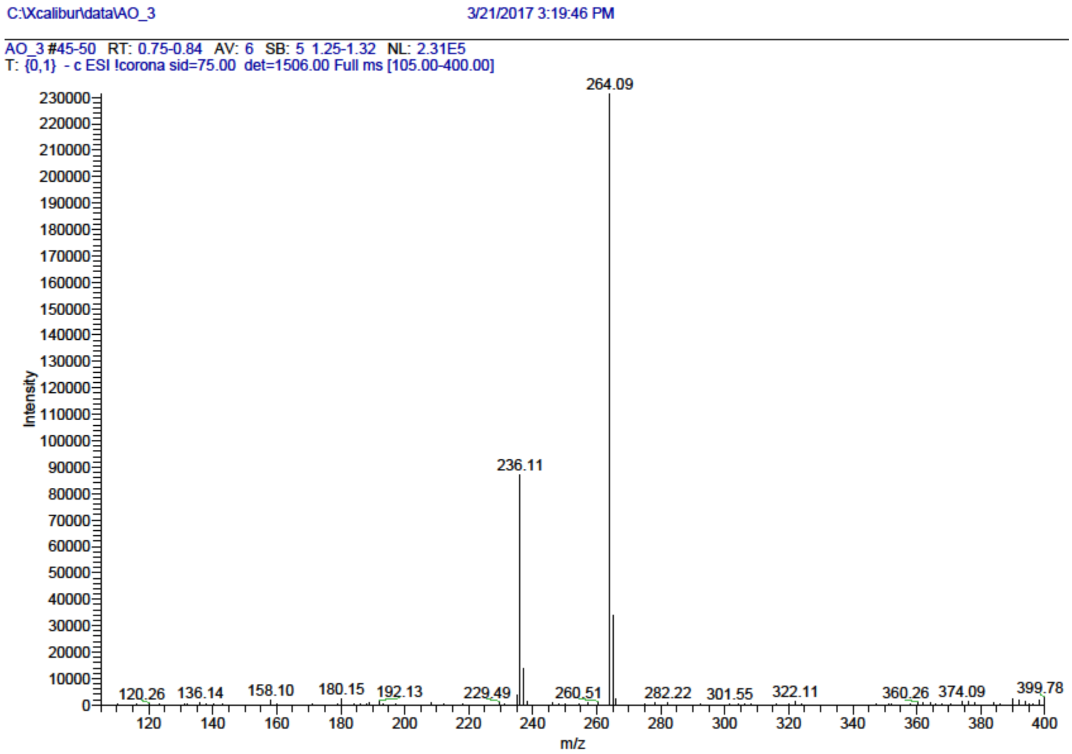

Figure S10. Mass (ESI$^-$) of 2-Hydroxy-3-methylen-(4-N-pyridyl)-1,4-naphtochinon (HNQ-3).

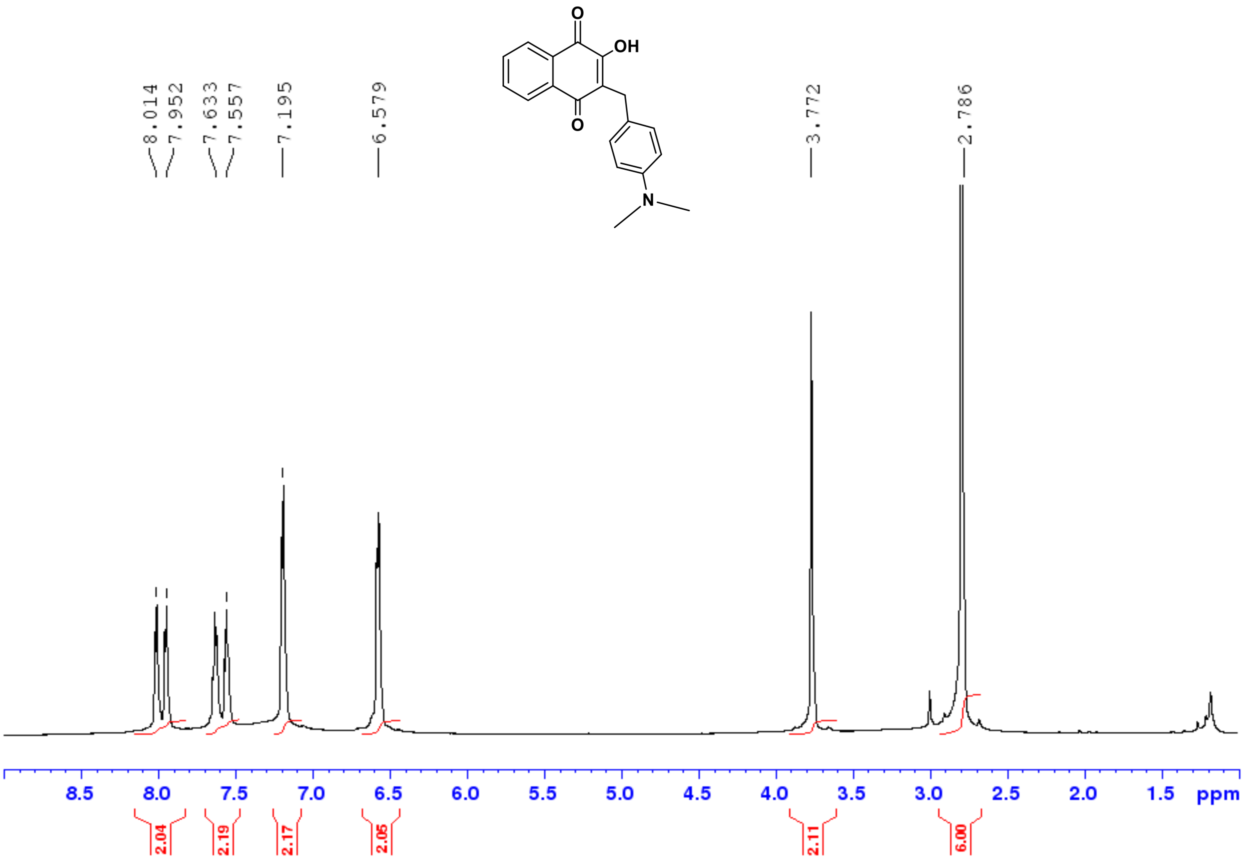

Figure S11. ^{1}H-NMR (600 MHz, $CDCl_3$) of 2-Hydroxy-3-methylen-(4-N,N-dimethylanilid)-1,4-naphtochinon (HNQ-4).

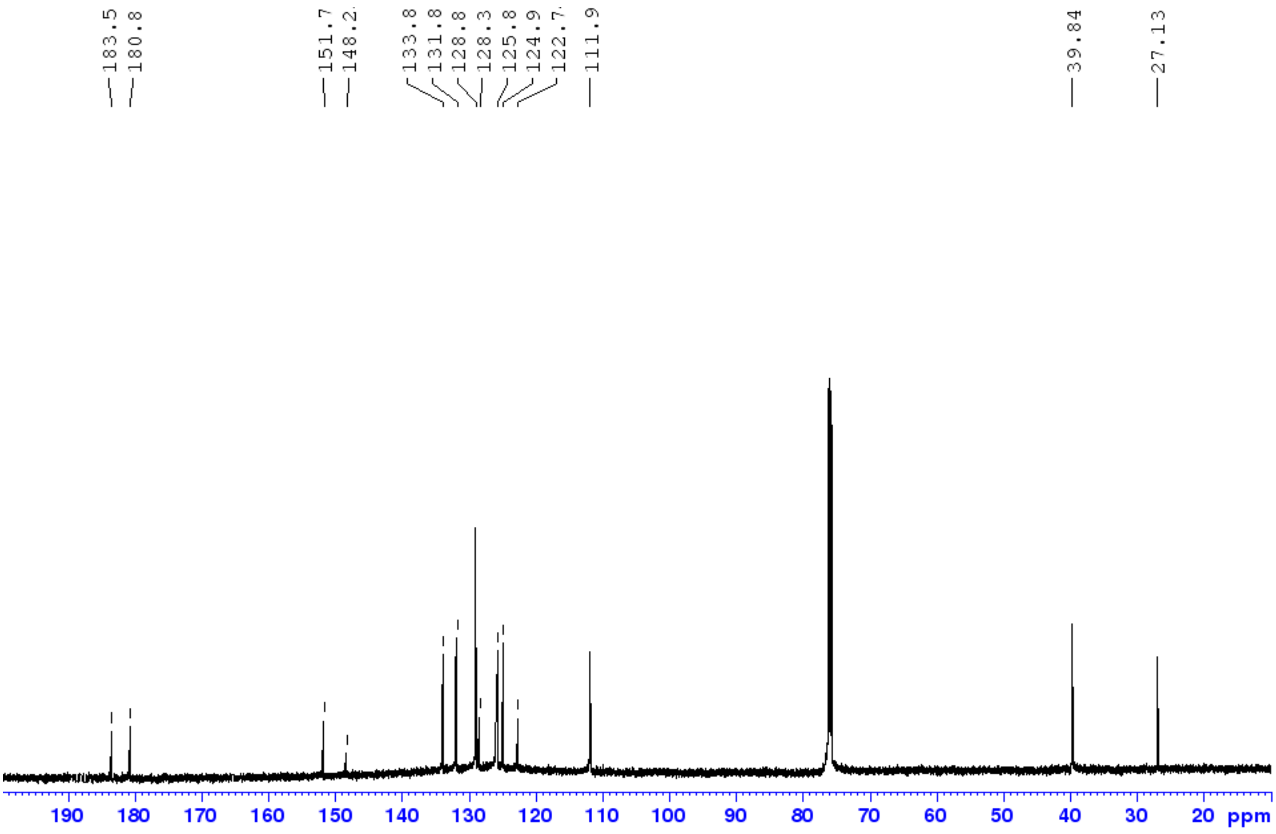

Figure S12. ^{13}C-NMR (150 MHz, $CDCl_3$) of 2-Hydroxy-3-methylen-(4-N,N-dimethylanilid)-1,4-naphtochinon (HNQ-4).

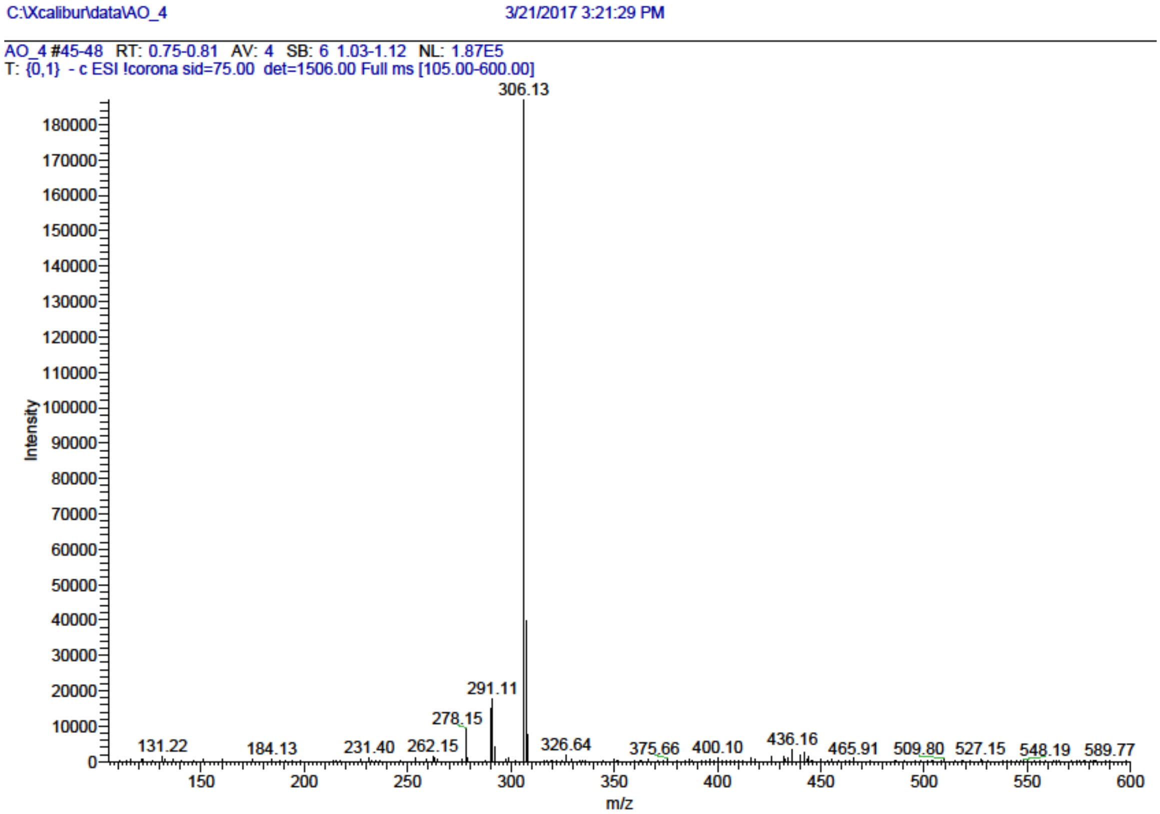

Figure S13. Mass (ESI$^-$) of 2-Hydroxy-3-methylen-(4-N,N-dimethylanilid)-1,4-naphtochinon (HNQ-4).

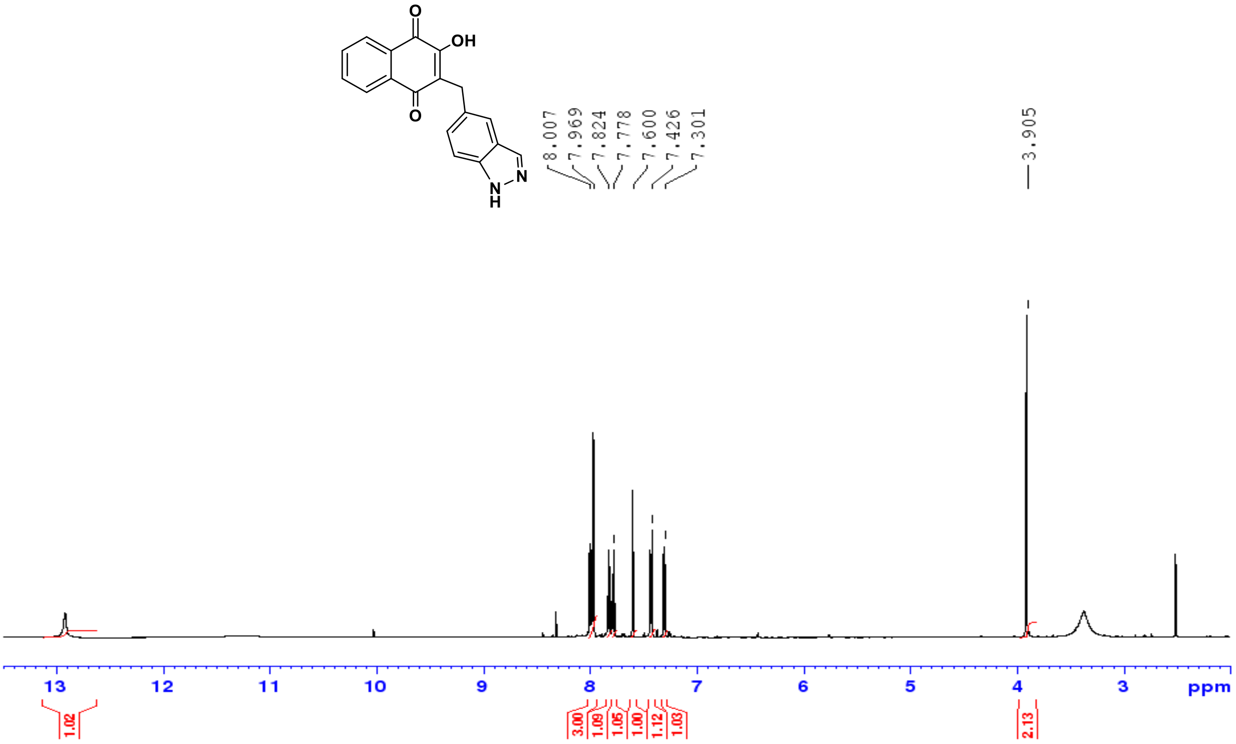

Figure S14. ^{1}H-NMR (600 MHz, DMSO-d$_6$) of 2-Hydroxy-3-[(indazol-5-yl)methyl]-1,4-naphthoquinone (HNQ-5).

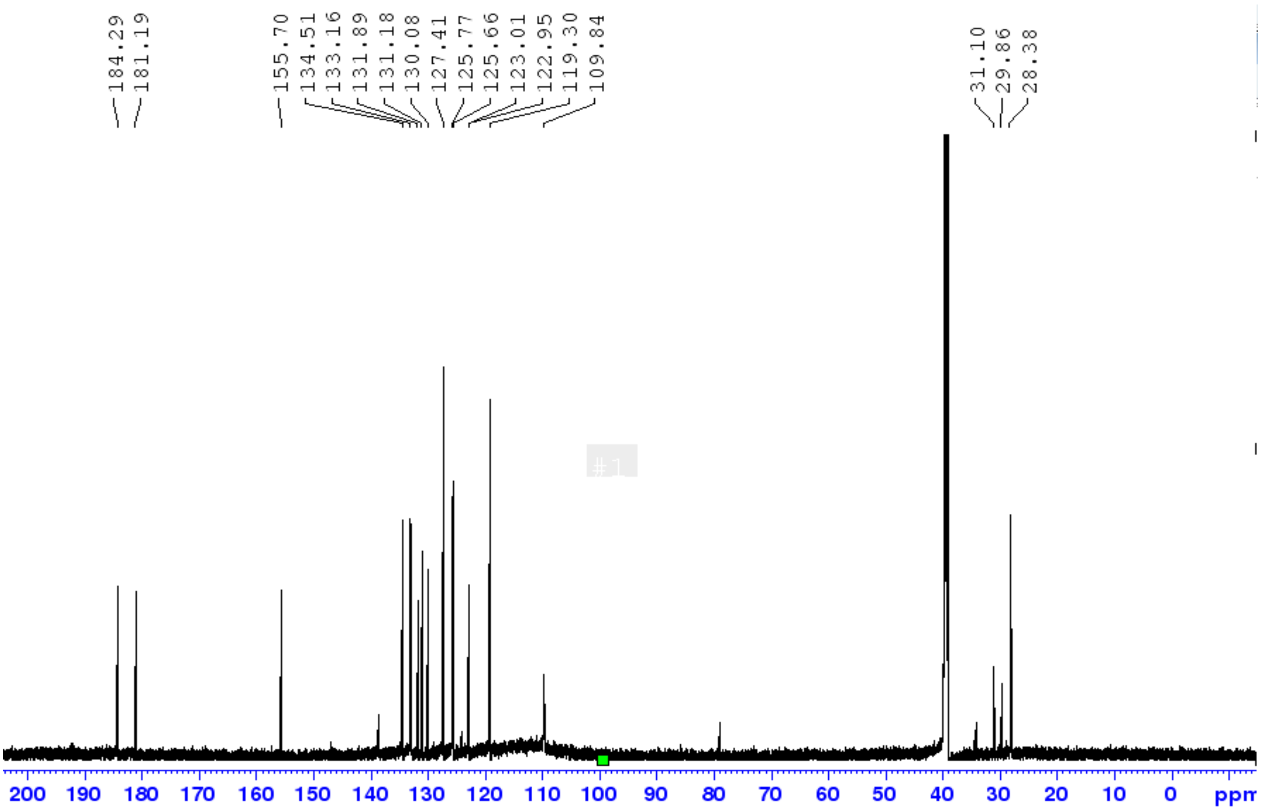

Figure S15. ^{13}C-NMR (150 MHz, DMSO-d_6) of 2-Hydroxy-3-[(indazol-5-yl)methyl]-1,4-naphthoquinone (HNQ-5).

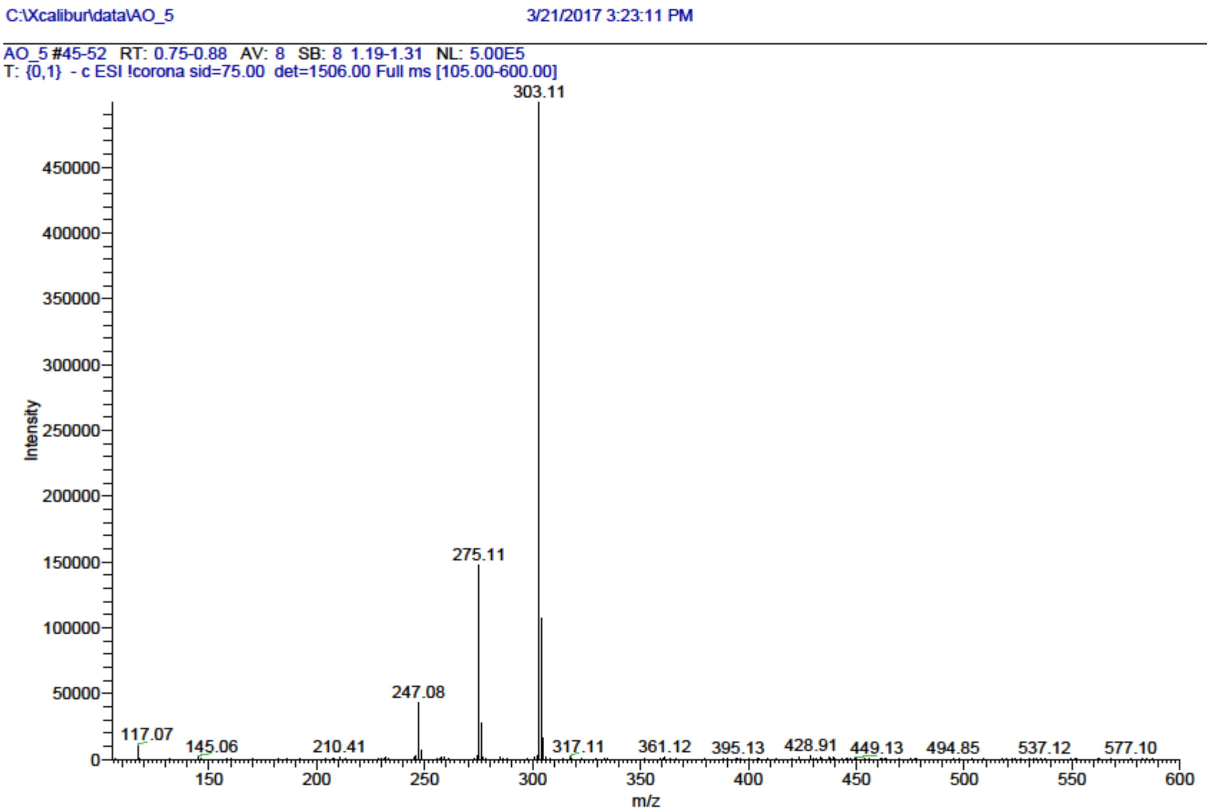

Figure S16. Mass (ESI$^-$) of 2-Hydroxy-3-[(indazol-5-yl)methyl]-1,4-naphthoquinone (HNQ-5).

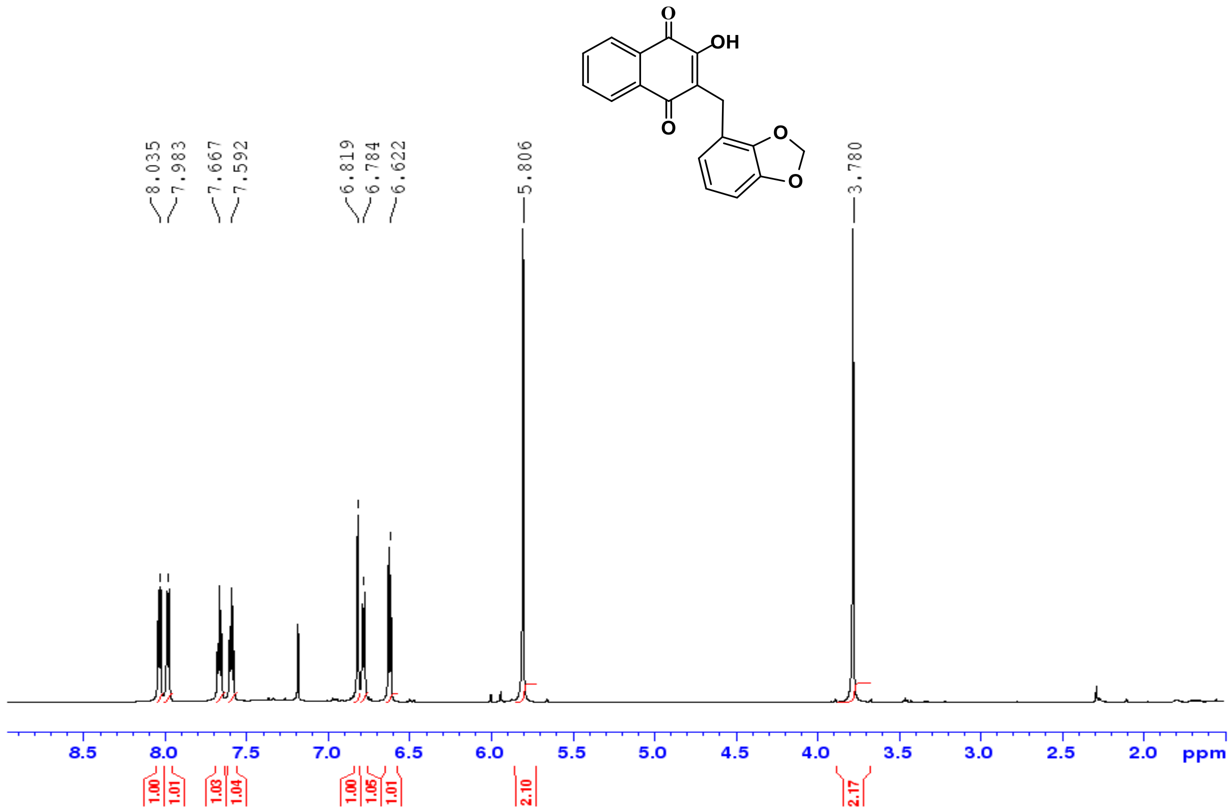

Figure S17. ^{1}H-NMR (600 MHz, $CDCl_3$) of 2-Hydroxy-3-[(1,2-methylenedioxybenz-5yl)methyl]-1,4-naphtoquinone (HNQ-6).

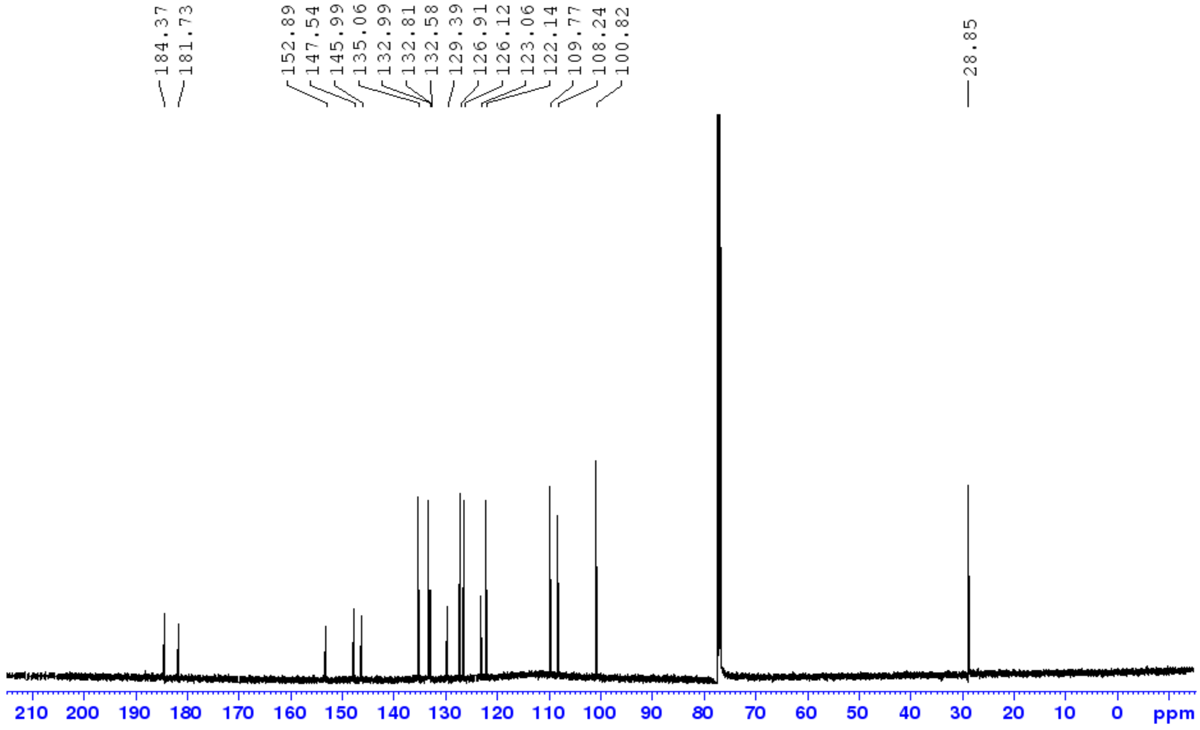

Figure S18. ^{13}C-NMR (150 MHz, $CDCl_3$) of 2-Hydroxy-3-[(1,2-methylenedioxybenz-5yl)methyl]-1,4-naphtoquinone (HNQ-6).

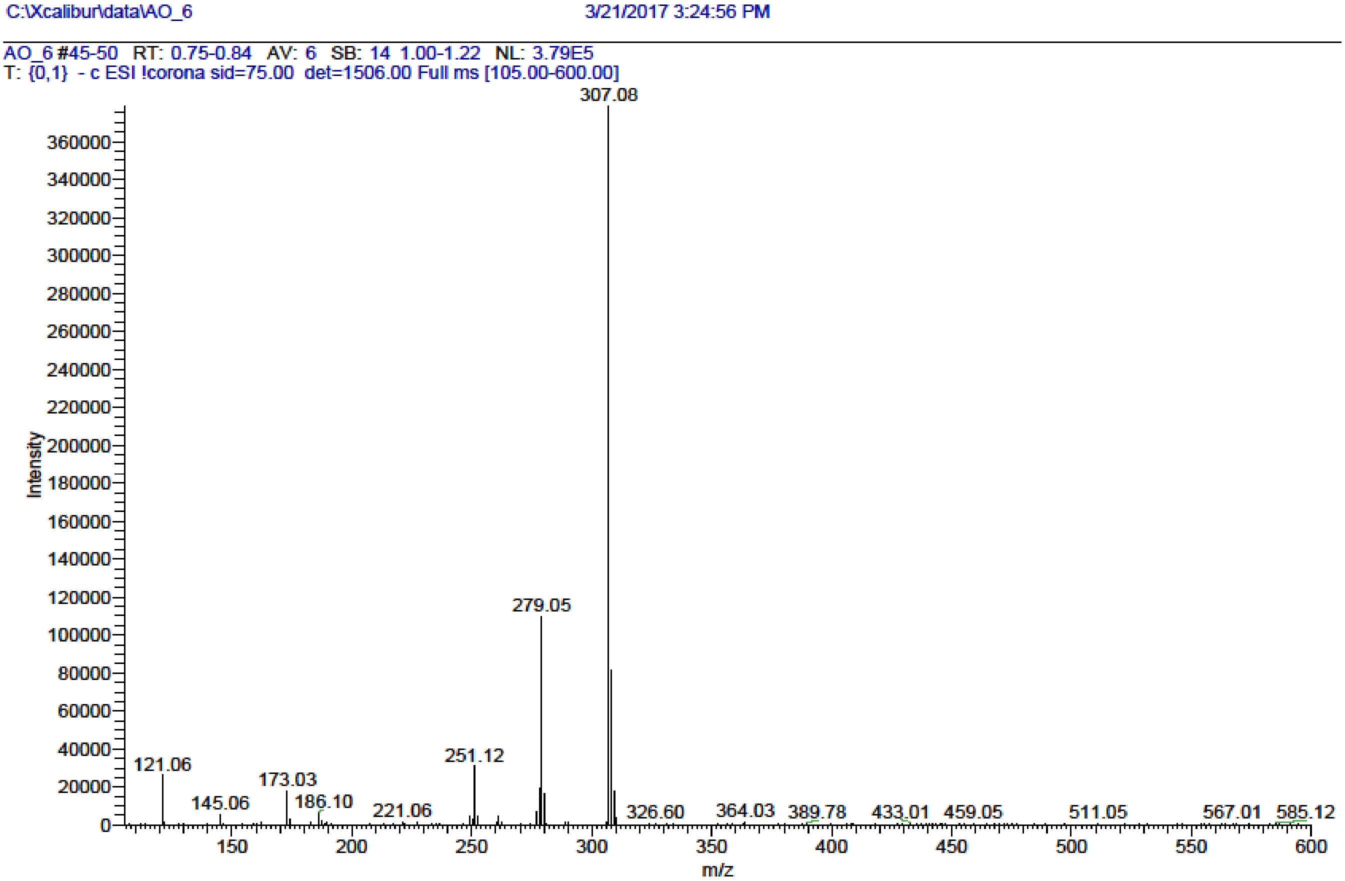

Figure S19. Mass (ESI$^-$) of 2-Hydroxy-3-[(1,2-methylenedioxybenz-5yl)methyl]-1,4-naphtoquinone (HNQ-6).

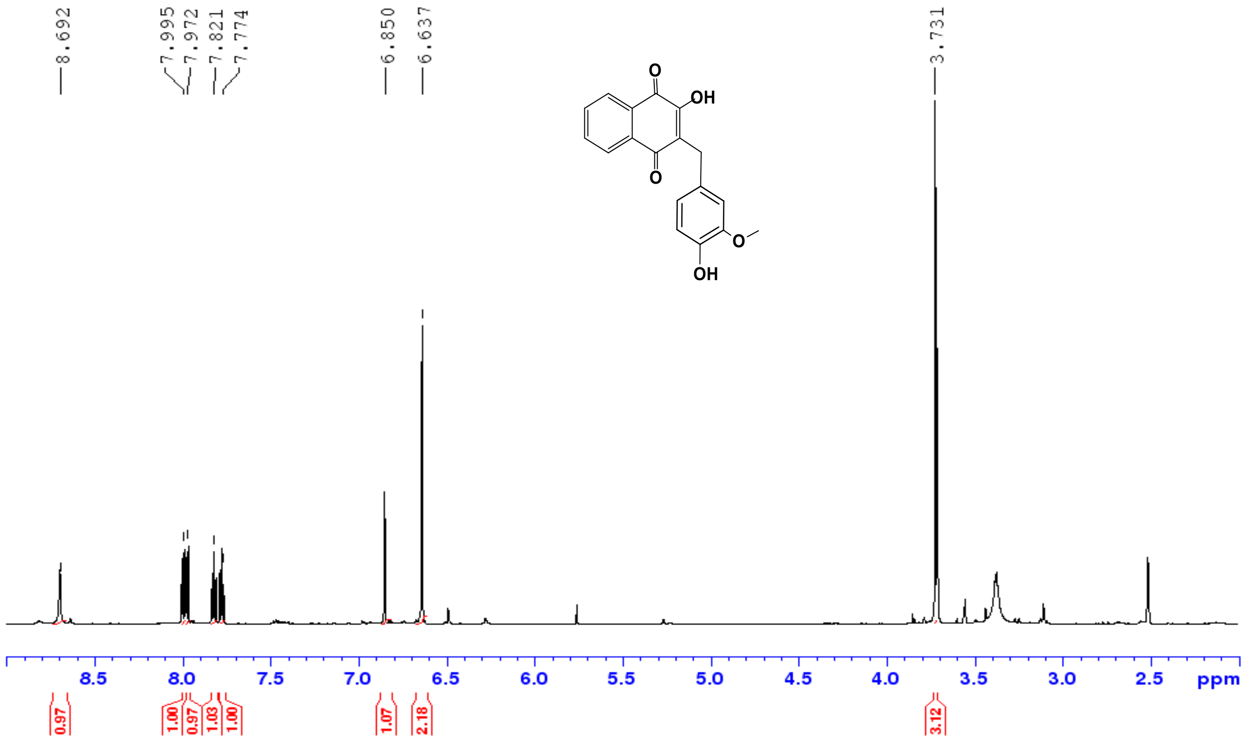

Figure S20. ^{1}H-NMR (600 MHz, DMSO-d_6) of 2-Hydroxy-3-[(4-hydroxy-3-methoxyphenyl)methyl]-1,4-naphtoquinone (HNQ-7).

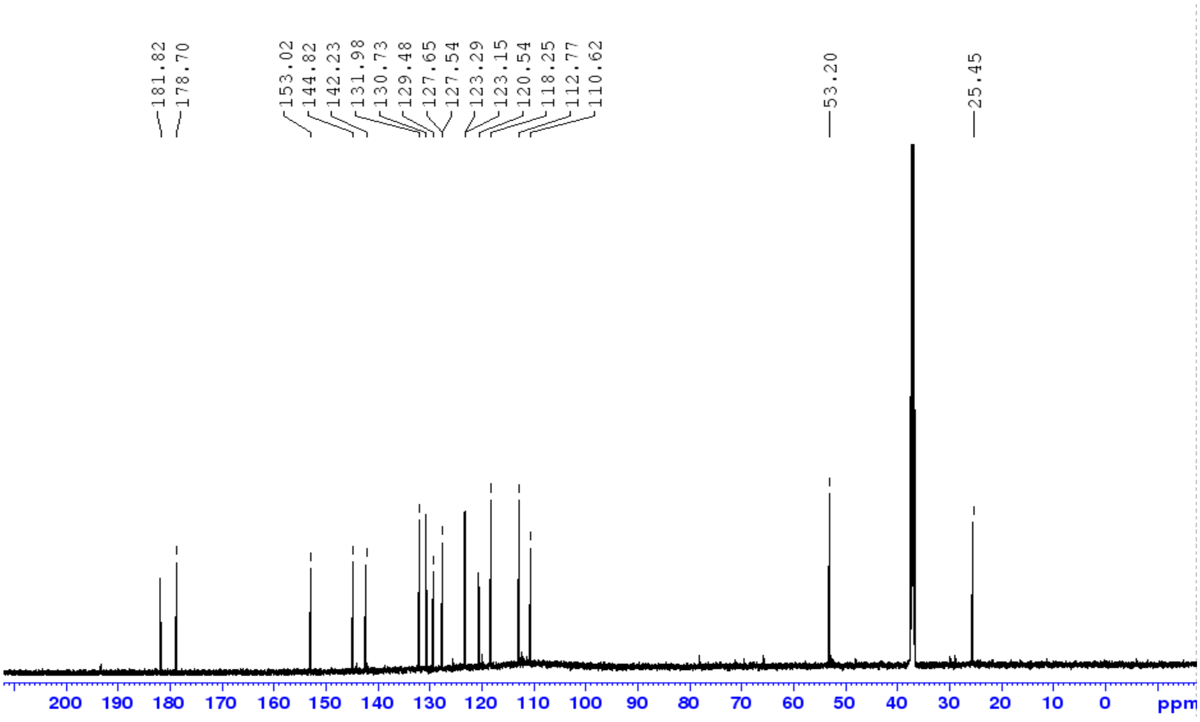

Figure S21. ^{13}C-NMR (150 MHz, DMSO-d$_6$) of 2-Hydroxy-3-[(4-hydroxy-3-methoxyphenyl)methyl]-1,4-naphtoquinone (HNQ-7).

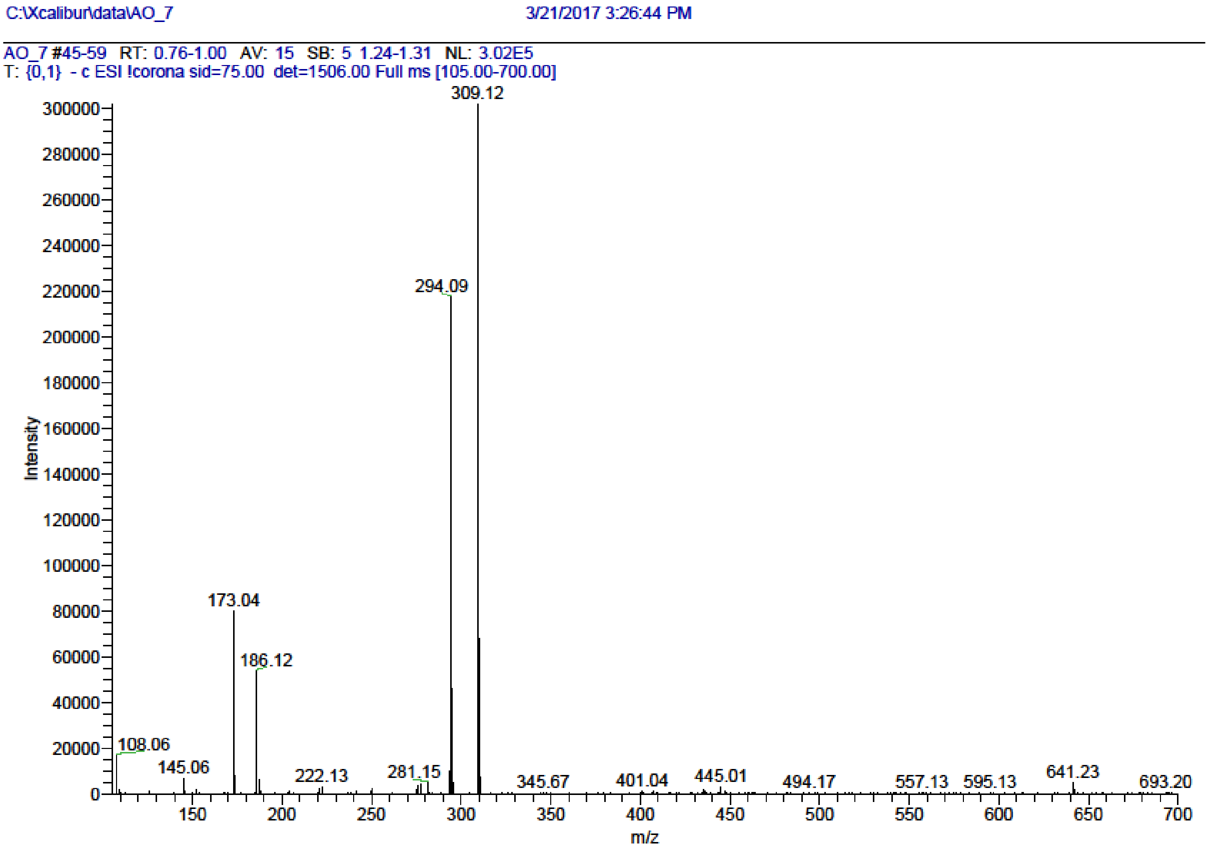

Figure S22. Mass (ESI$^-$) of 2-Hydroxy-3-[(4-hydroxy-3-methoxyphenyl)methyl]-1,4-naphtoquinone (HNQ-7).

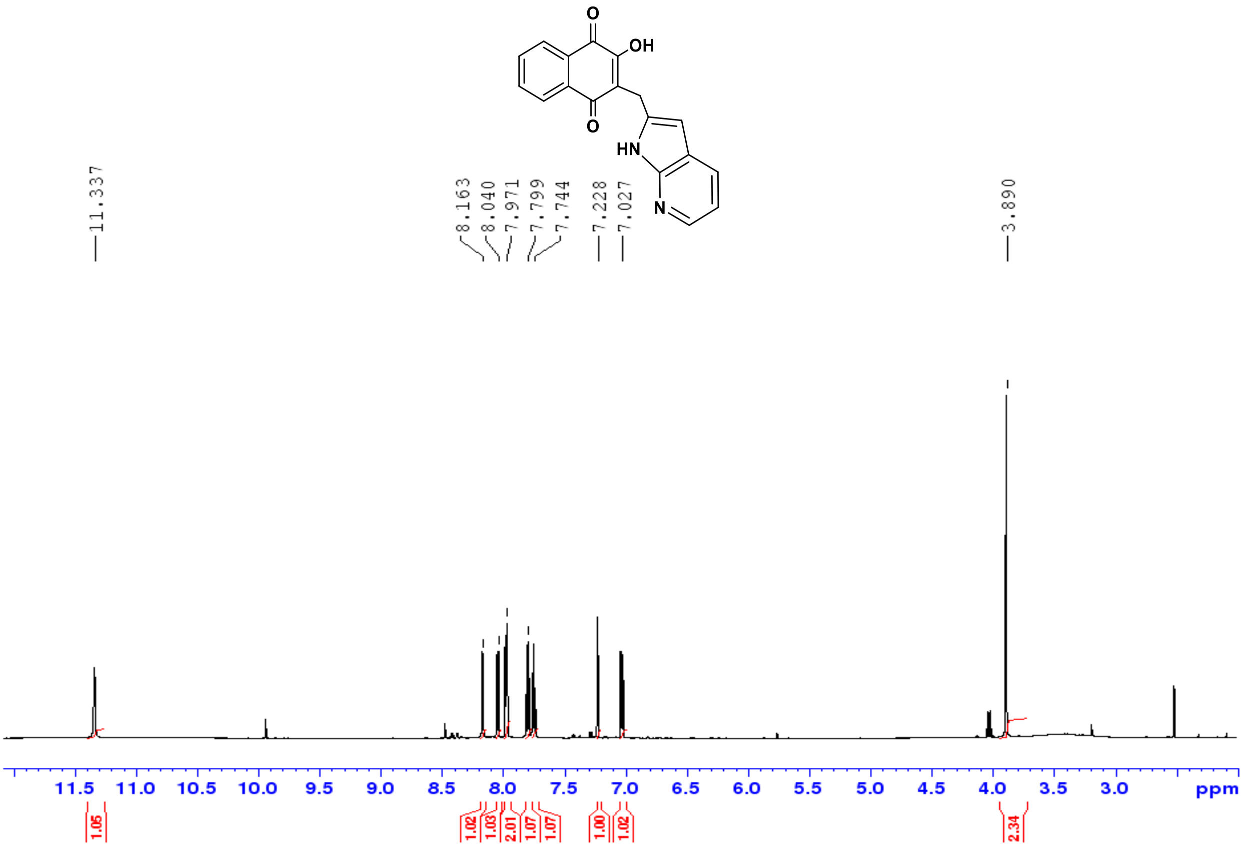

Figure S23. ^{1}H-NMR (600 MHz, DMSO-d_6) of 2-Hydroxy-3-[(1H-pyrrolo(2,3-b)pyridine-3-yl)methyl]-1,4-naphtoquinone (HNQ-8).

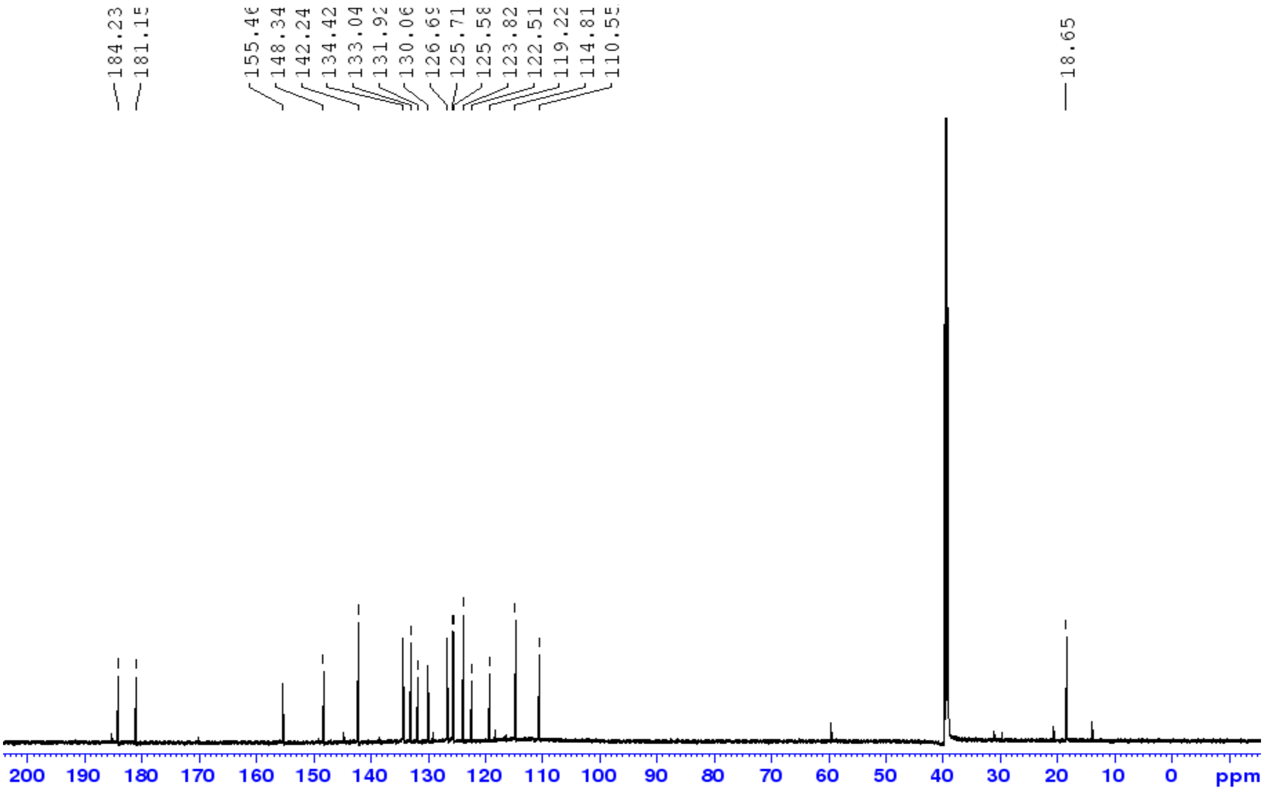

Figure S24. ^{13}C-NMR (150 MHz, DMSO-d_6) of 2-Hydroxy-3-[(1H-pyrrolo(2,3-b)pyridine-3-yl)methyl]-1,4-naphtoquinone (HNQ-8).

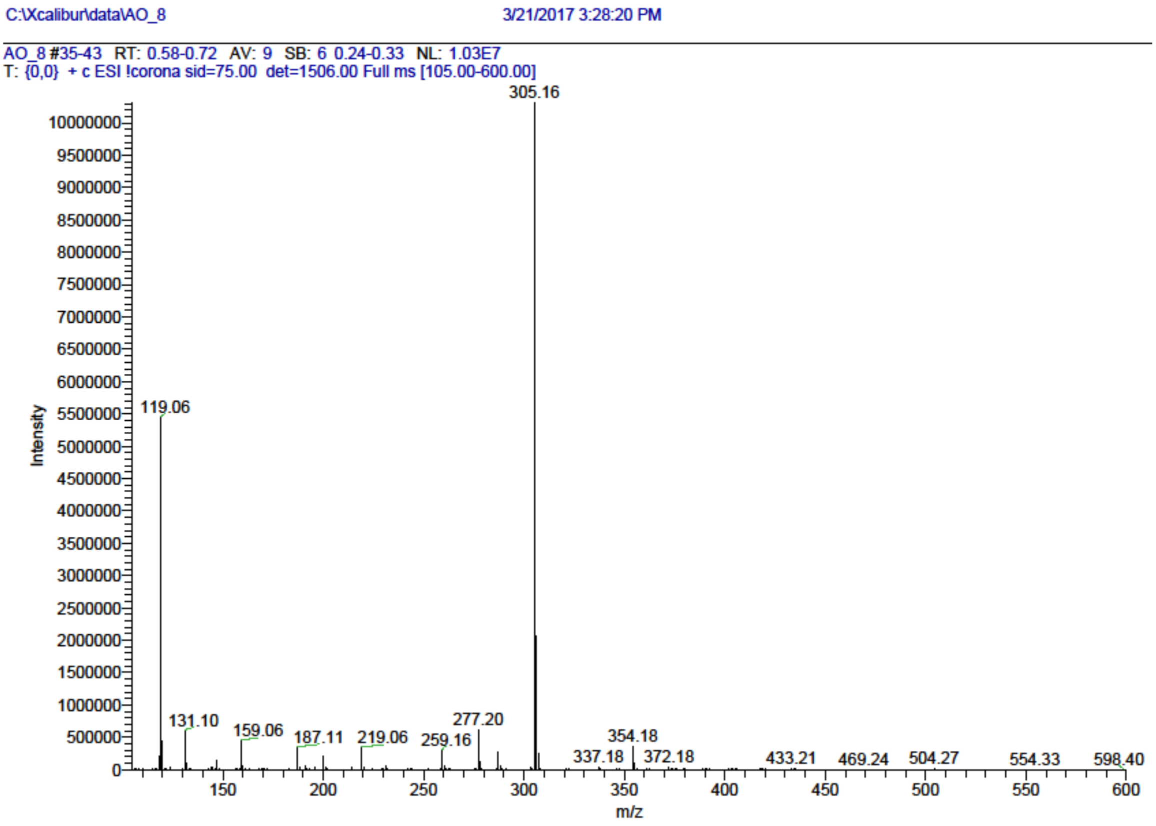

Figure S25. Mass (ESI+) of 2-Hydroxy-3-[(1H-pyrrolo(2,3-b)pyridine-3-yl)methyl]-1,4-naphtoquinone (HNQ-8).

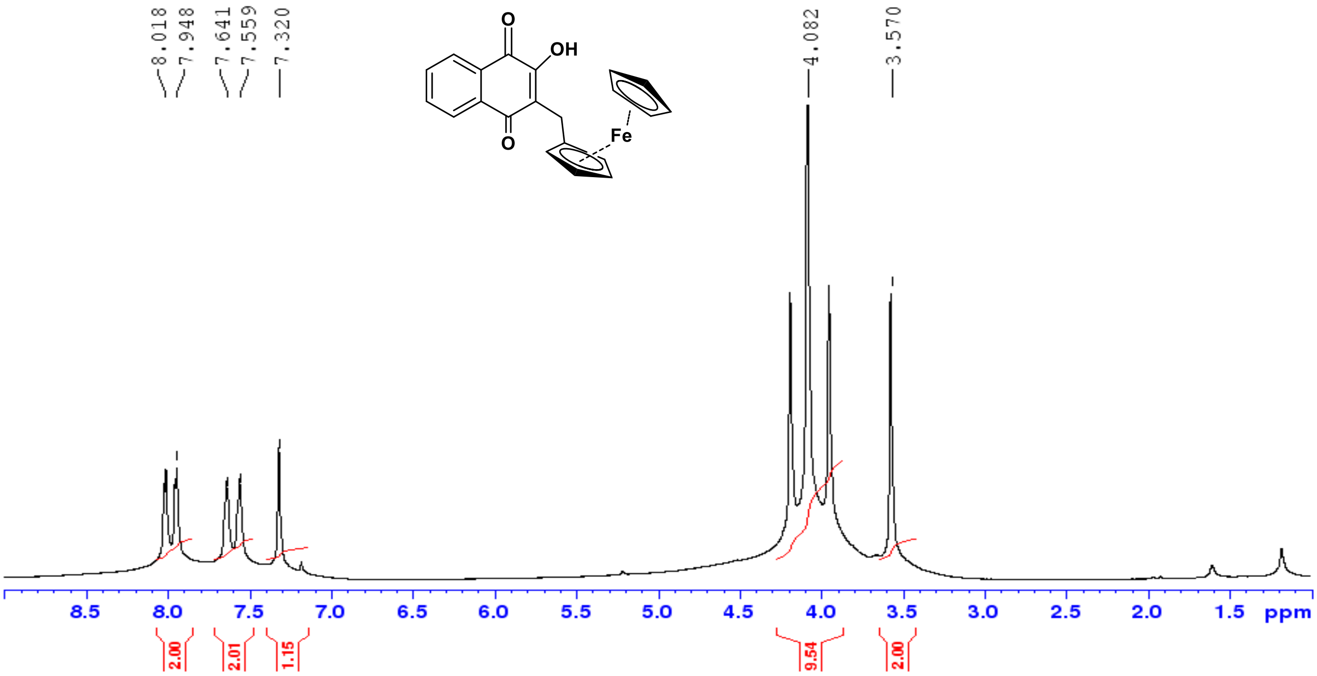

Figure S26. ^{1}H-NMR (600 MHz, $CDCl_3$) of 2-Hydroxy-3-[(ferreocenyl)methyl]-1,4-naphthoquinone (HNQ-9).

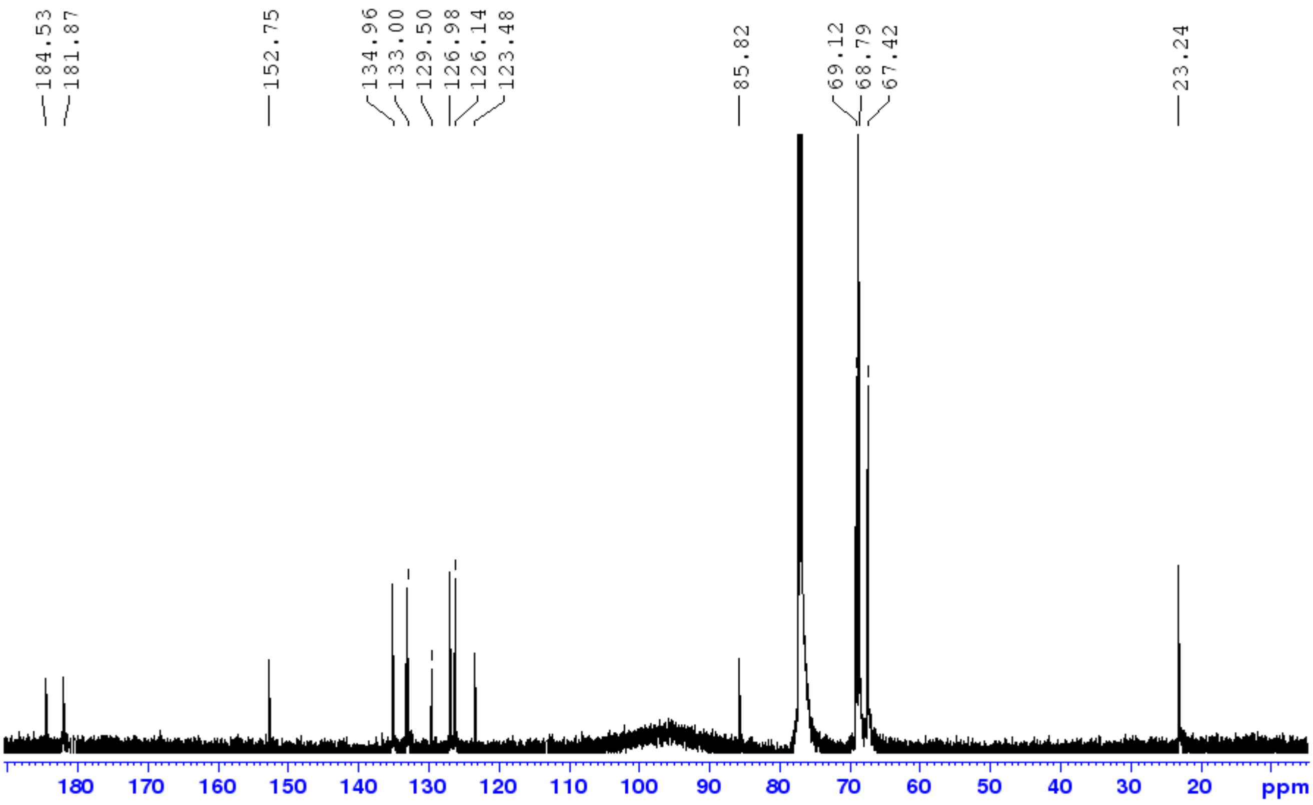

Figure S27. ^{13}C-NMR (600 MHz, $CDCl_3$) of 2-Hydroxy-3-[(ferreocenyl)methyl]-1,4-naphthoquinone (HNQ-9).

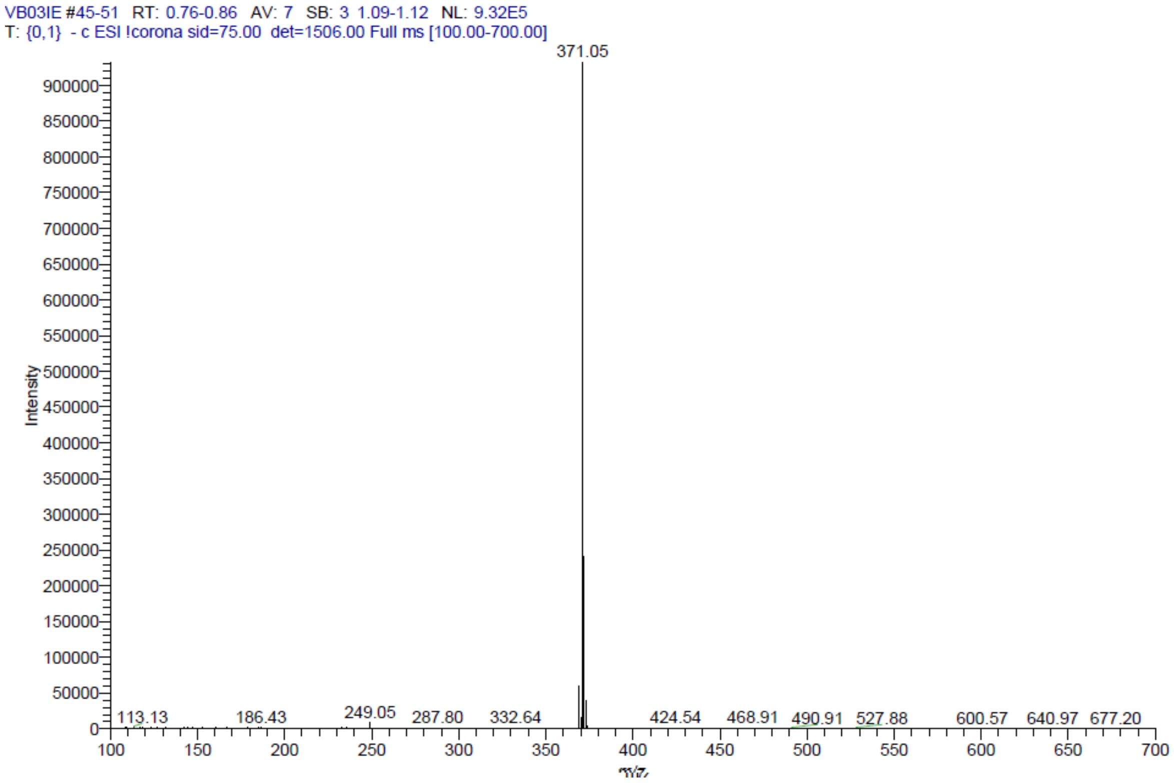

Figure S28. Mass (ESI$^-$) of 2-Hydroxy-3-[(ferreocenyl)methyl]-1,4-naphthoquinone (HNQ-9).

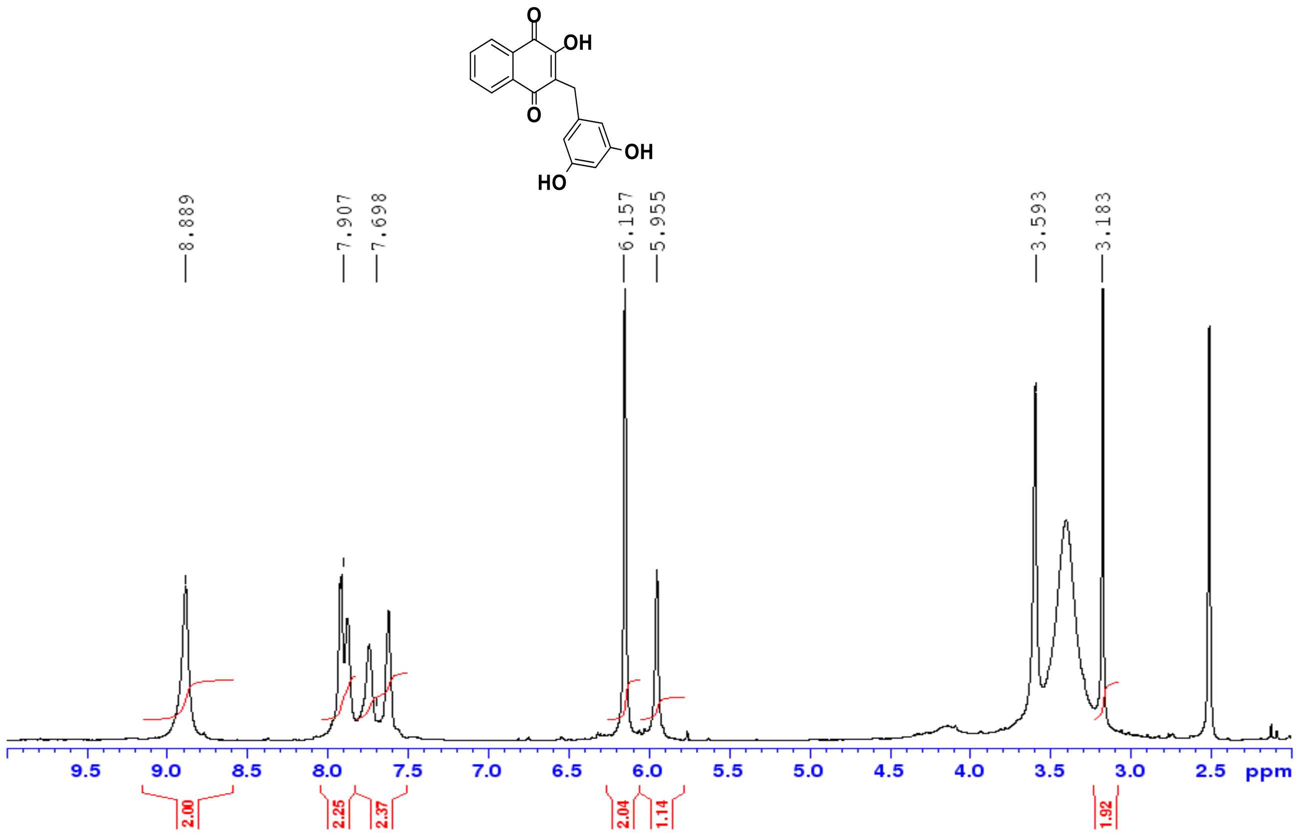

Figure S29. ^{1}H-NMR (600 MHz, MeOD) of 2-Hydroxy-3-[(3,5-dihydroxybenzyl)methyl]-1,4-naphthoquinone (HNQ-10).

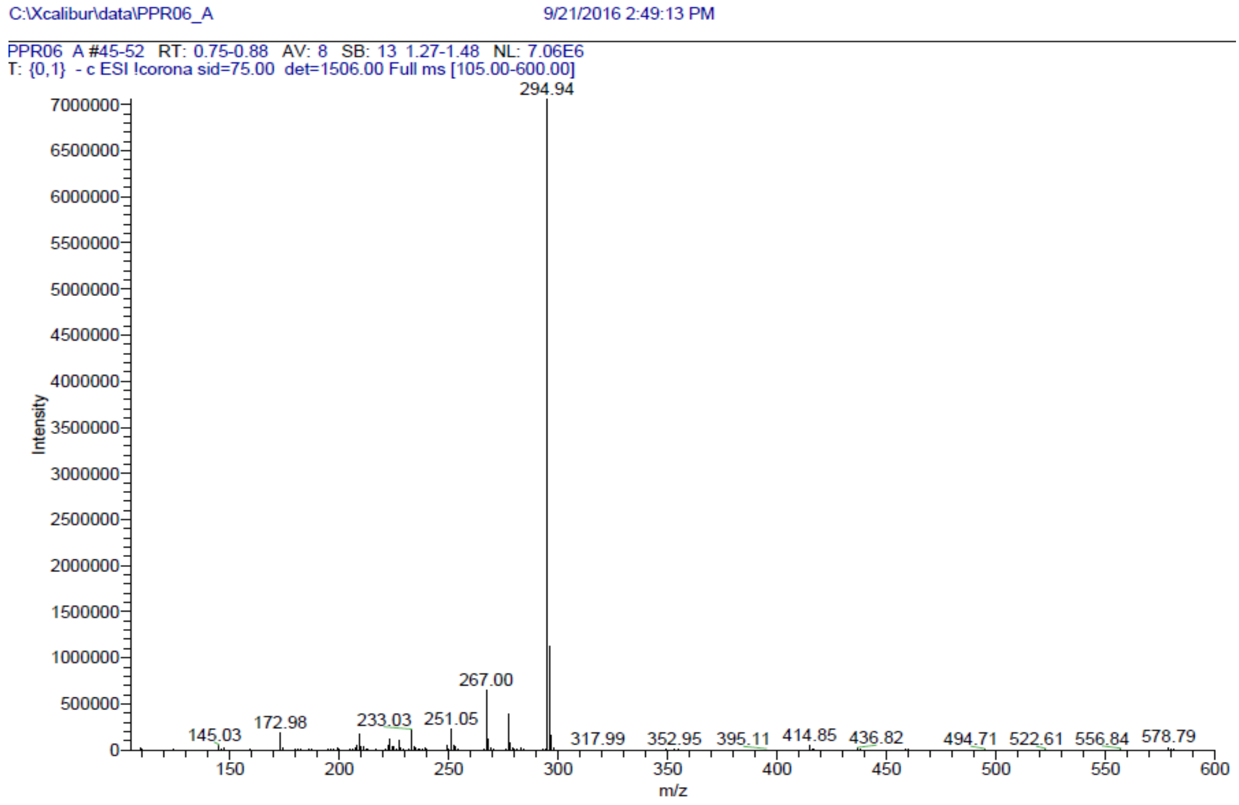

Figure S30. ^{13}C-NMR (600 MHz, MeOD) of 2-Hydroxy-3-[(3,5-dihydroxybenzyl)methyl]-1,4-naphthoquinone (HNQ-10)

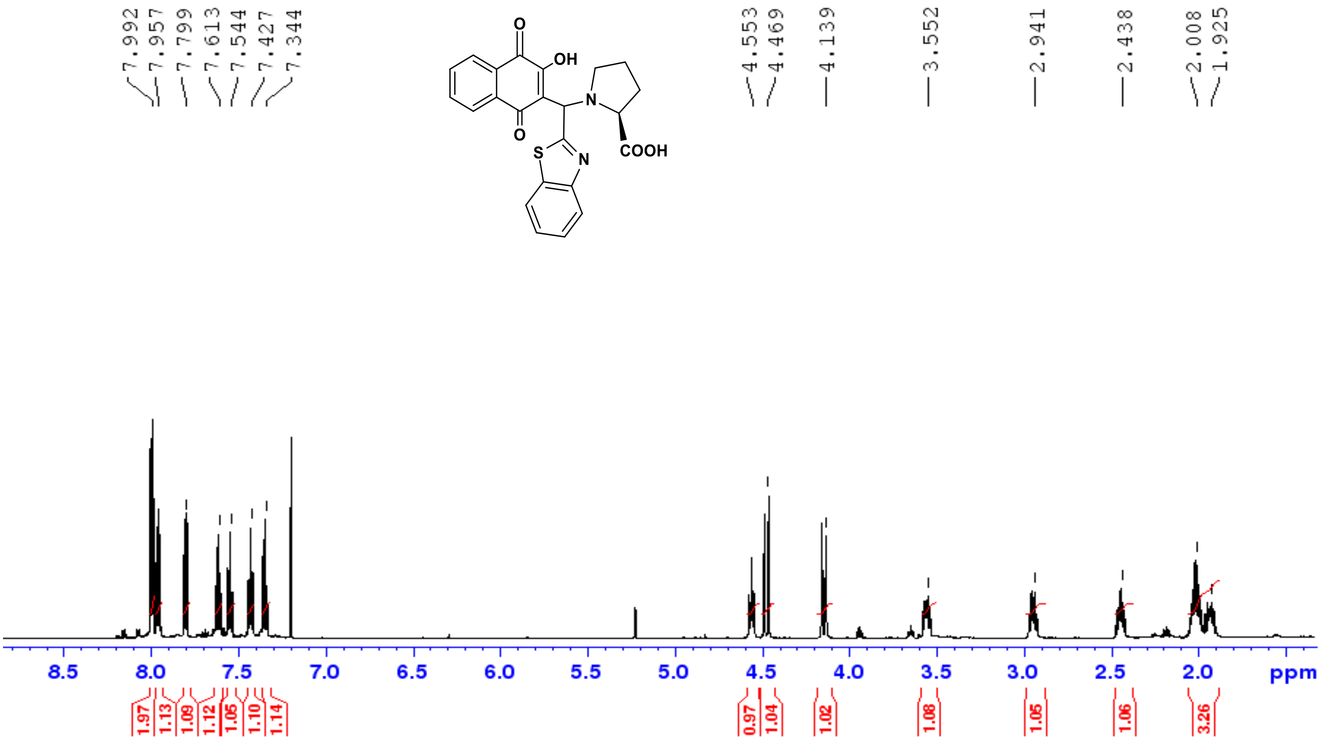

Figure S31. ^{1}H-NMR (600 MHz, $CDCl_3$) of 2-Hydroxy-3-[(2-thiazol(2-3)benzyl)methyl-pyrroidine-2-carboxylic acid]-1,4-naphthoquinone (HNQ-11).

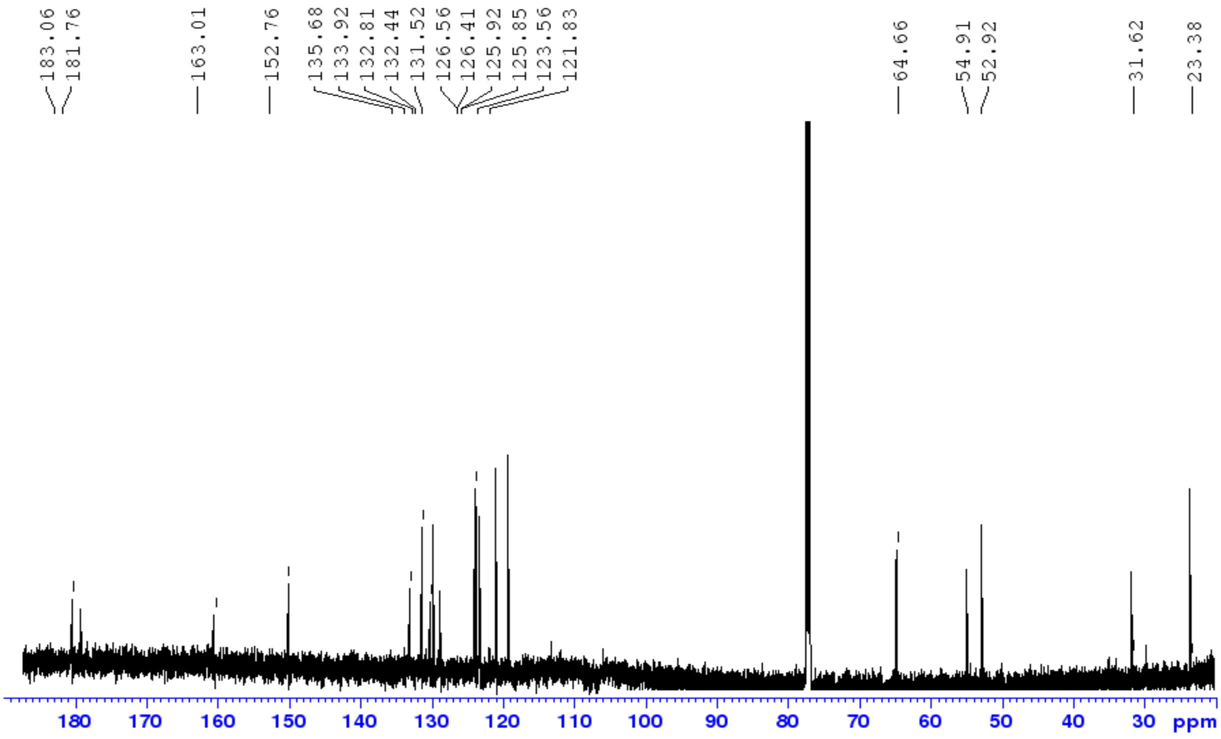

Figure S32. ^{1}H-NMR (600 MHz, $CDCl_3$) of 2-Hydroxy-3-[(2-thiazol(2-3)benzyl)methyl-pyrroidine-2-carboxylic acid]-1,4-naphthoquinone (HNQ-11).

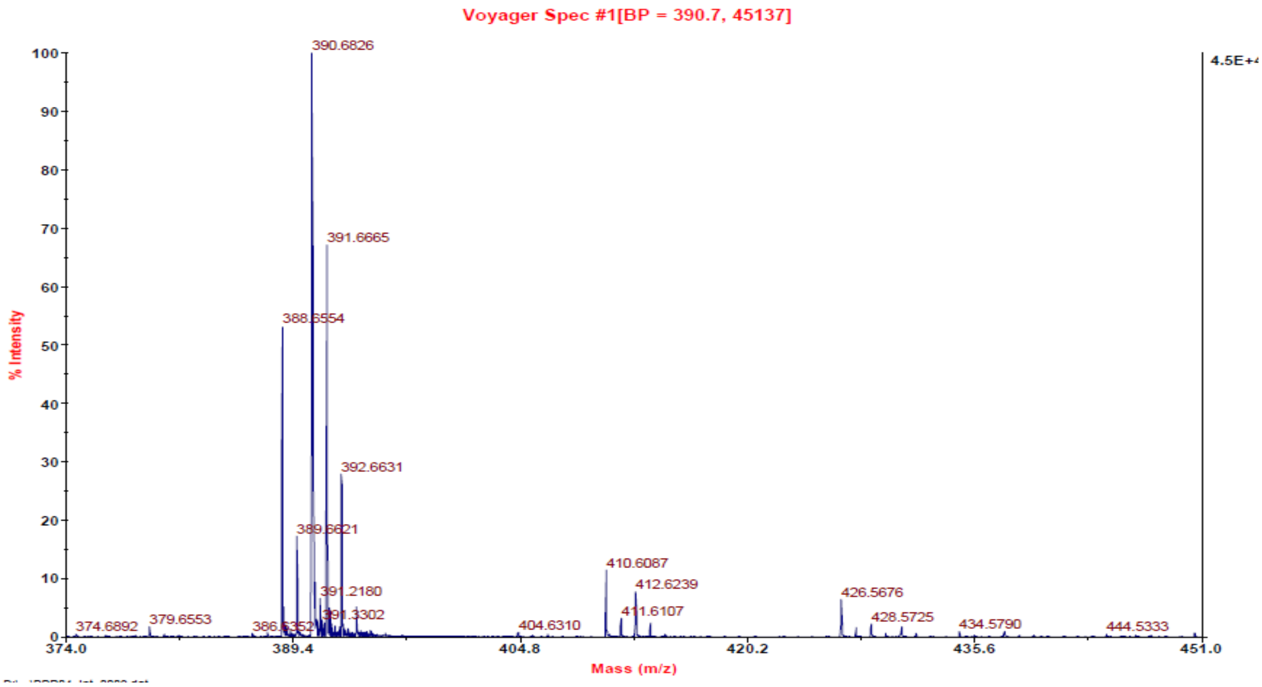

Figure S33. Mass (ESI⁻) of 2-Hydroxy-3-[(2-thiazol(2-3)benzyl)methyl-pyrroidine-2-carboxylic acid]-1,4-naphthoquinone (HNQ-11).

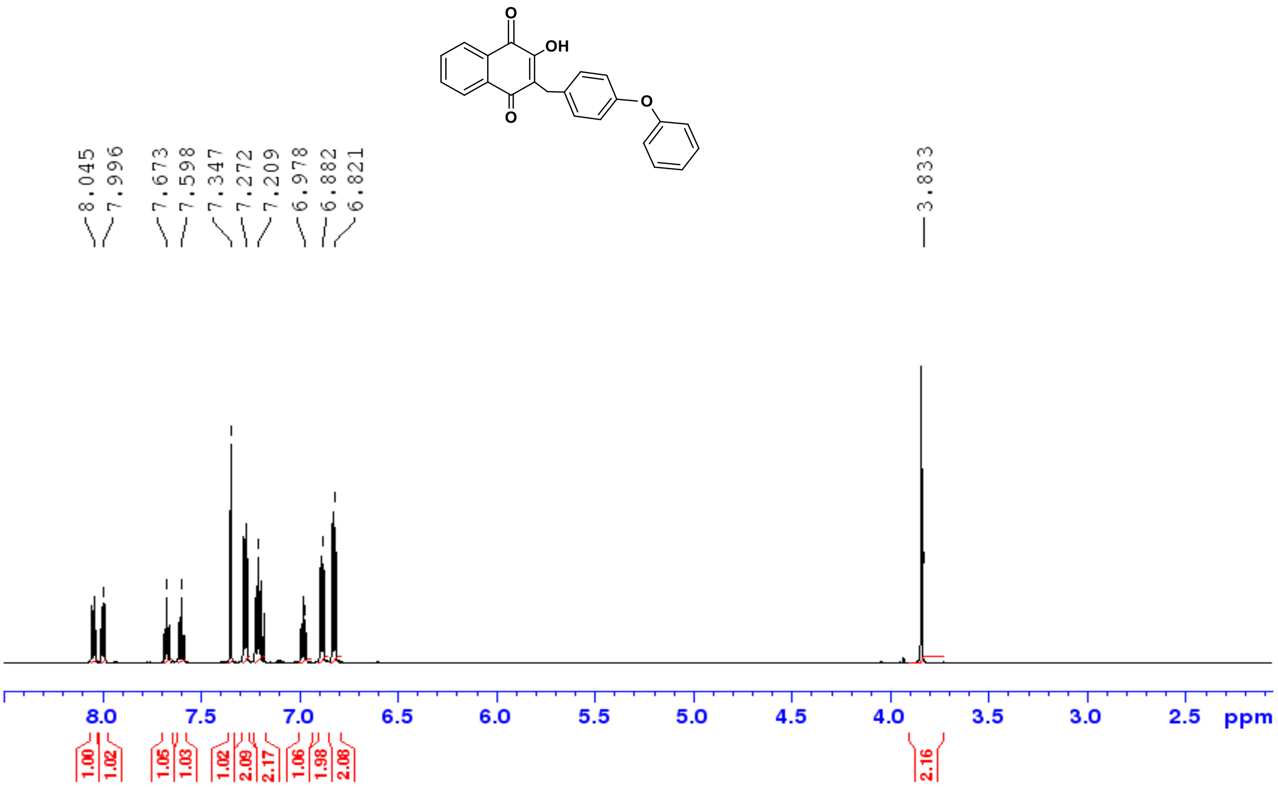

Figure S34. ^{1}H-NMR (600 MHz, DMSO-d_6) of 2-Hydroxy-3-[(4-phenoxybenzyl)methyl]-1,4-naphthoquinone (HNQ-12).

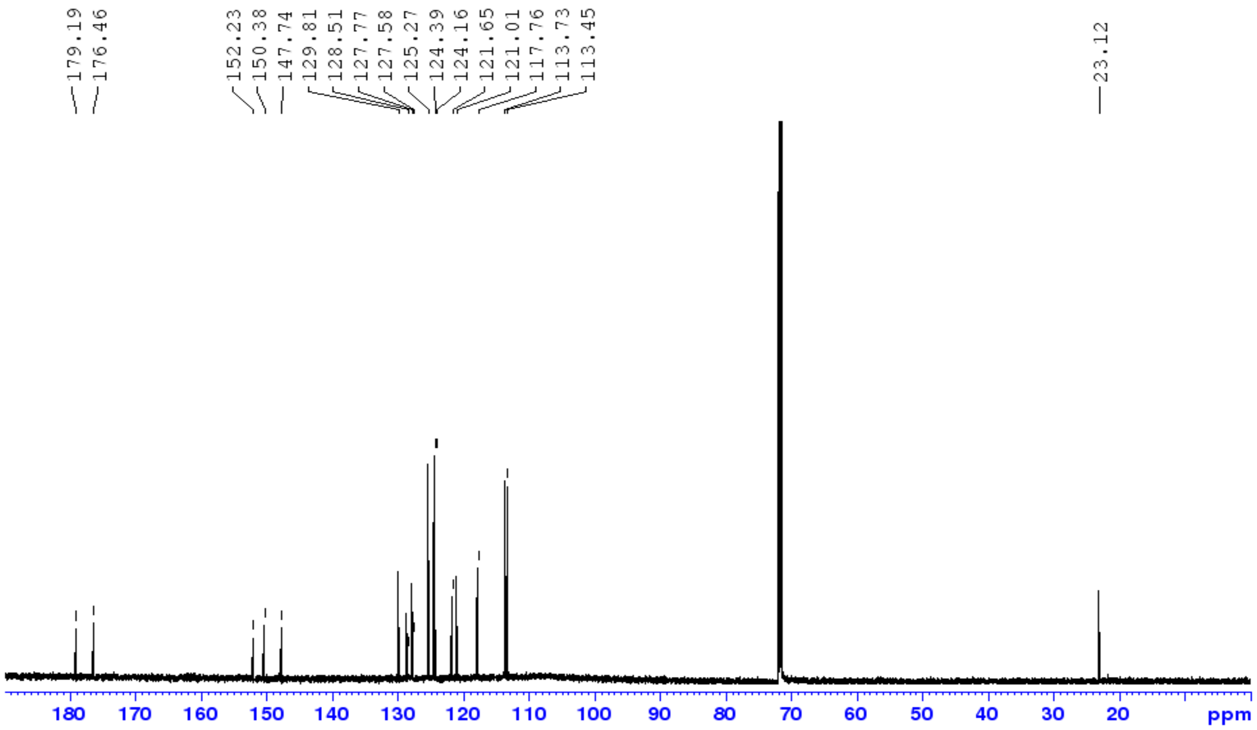

Figure S35. ^{13}C-NMR (600 MHz, DMSO-d_6) of 2-Hydroxy-3-[(4-phenoxybenzyl)methyl]-1,4-naphthoquinone (HNQ-12).

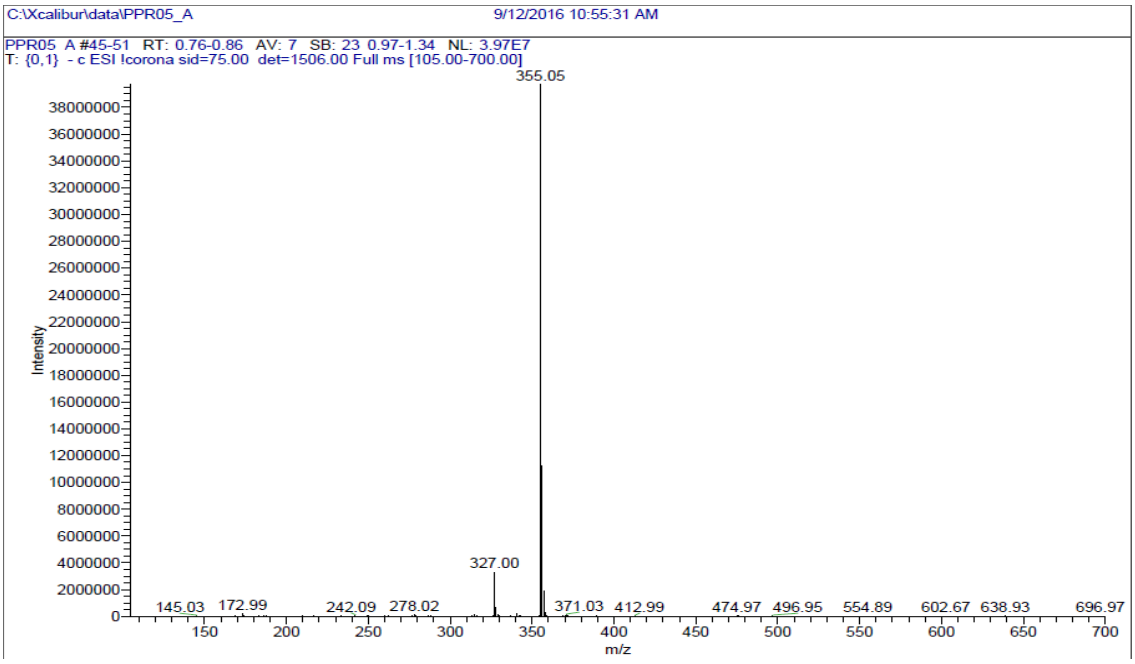

Figure S36. Mass (ESI$^-$) of 2-Hydroxy-3-[(4-phenoxybenzyl)methyl]-1,4-naphthoquinone (HNQ-12).

3. Solubility profiles of HNQs

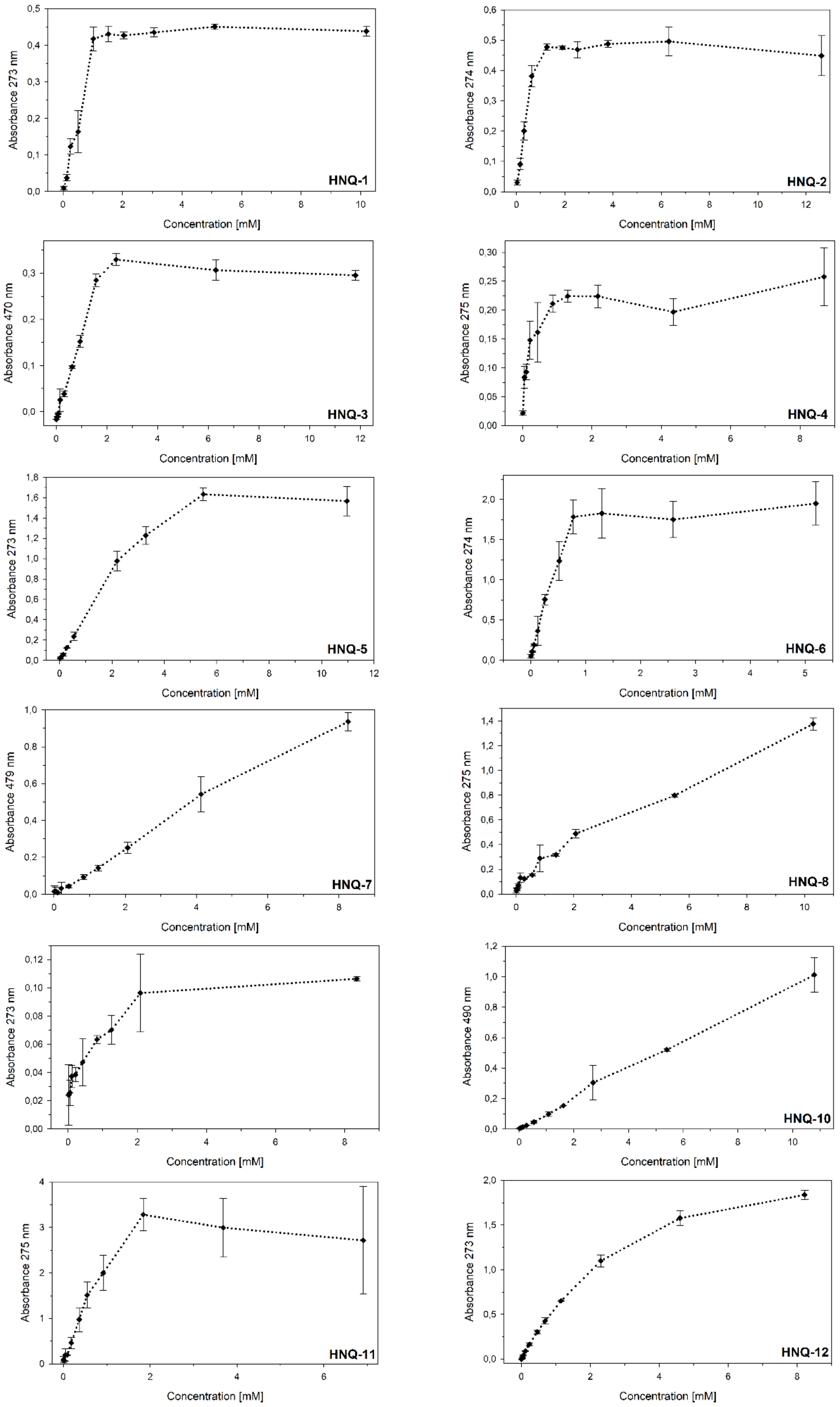

Figure S37: Solubility profiles of HNQ compounds in buffer (20 mM NaPi, 50 mM NaCl, 0,02% DDM, pH 8.0) at room temperature and different detection wavelengths.

4. Stock and assayed concentrations of HNQs

Table S1: Stock concentrations of HNQ-compounds in methanol and DCM, concentrations for particular measurements.
* Solvent of HNQ-9 was DCM, because of extremely low solubility in Methanol.

Compound	stock concentration [mg/mL]	mass concentration range [µg/mL]	molar concentration range [mM]
**HNQ-1**	1.673	8.37 – 3346	0.03 – 10.20
**HNQ-2**	1.669	8.35 – 3338	0.03 – 12.64
**HNQ-3**	0.523	2.09 – 3135	0.008 – 11.81
**HNQ-4**	1.668	3.37 – 2669	0.01 – 8.69
**HNQ-5**	1.669	8.35 – 3338	0.03 – 11.00
**HNQ-6**	2	2 – 3000	0.007 – 5.19
**HNQ-7**	1.673	3.35 – 2677	0.01 – 8.26
**HNQ-8**	0.523	2.09 – 3135	0.007 – 10.30
**HNQ-9***	2.1	4.2 – 3360	0.01 – 8.35
**HNQ-10**	2	4 – 3200	0.01 – 10.80
**HNQ-11**	2	2 – 3000	0.005 – 6.90
**HNQ-12**	0.293	2.05 – 2929	0.006 – 8.22

5. Simulated physicochemical properties of tested compounds

Table S2: Simulated physicochemical properties of tested compounds. *HNQ-9 no data available

Compound	MW	Log P from file	Log P Optibrium	Log S Optibrium	Log D Optibrium @ pH 7,4
**HNQ-1**	328.45	5.80	5.49	0.34	1.82
**HNQ-2**	264.28	3.12	3.22	0.98	0.55
**HNQ-3**	265.27	2.52	2.50	1.28	-0.16
**HNQ-4**	307.35	3.19	3.40	1.36	0.86
**HNQ-5**	304.30	3.00	2.70	0.61	0.47
**HNQ-6**	308.29	2.85	2.86	0.49	0.79
**HNQ-7**	310.30	2.83	3.04	0.84	0.13
**HNQ-8**	304.30	3.00	3.08	0.78	0.45
**HNQ-10**	296.28	2.53	2.70	0.79	-0.49
**HNQ-11**	434.47	3.78	3.68	0.75	-0.13
**HNQ-12**	356.38	4.91	4.62	-0.85	1.64
**Q1 red**	865.37	18.47	5.88	1.31	5.88
**Q1 ox**	863.36	17.85	5.25	1.51	5.25

Optibrium data in Table S2 were generated with SeeSAR version 9.2; BioSolveIT GmbH, Sankt Augustin, Germany, 2019, www.biosolveit.de/SeeSAR

6. Purification of *E. coli* respiratory cytochrome *bo₃* and *bd-I* oxidases

Cytochrome *bo₃*

A) SDS PAGE *cyt bo₃* **B) Native PAGE cyt *bo₃***

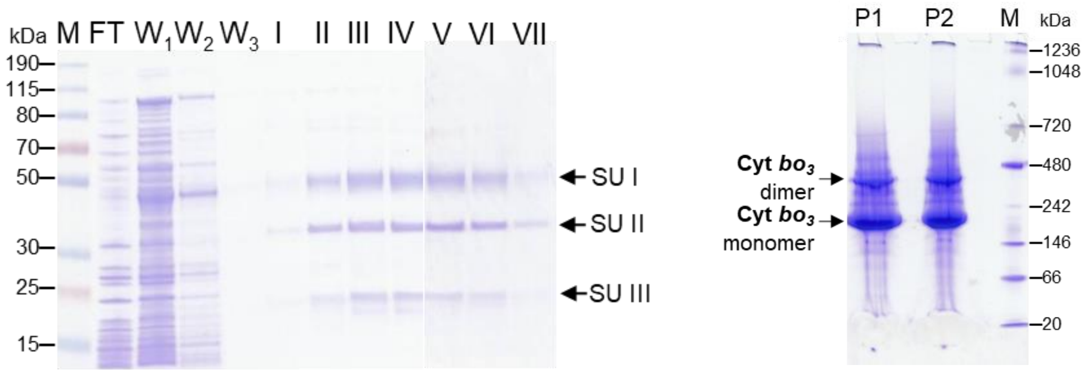

Figure S38: A) SDS PAGE in MES buffer; gradient polyacrylamid gel 4-12 %. M: marker; FT: Flow through; WF_{1-3}: wash fraction 1-3; I-VII: Elution fractions 1-7 with Subunits I-III (SU I-III); concentration was 4,2 mg/ml with a mass of 150 kDa B) Clear Native PAGE 4-16 % Bis-Tris gel, stained with Coomassie blue: M: marker; P1: 60 µg cyt *bo₃* before dialysis; P2: 60 µg cyt *bo₃* after dialysis (4,2 mg/ml; 28 µM; 150 kDa).

Cytochrome *bd-I* type oxidase

A) SDS PAGE *bd-I* type oxidase **B) Native PAGE *bd-I* type oxidase**

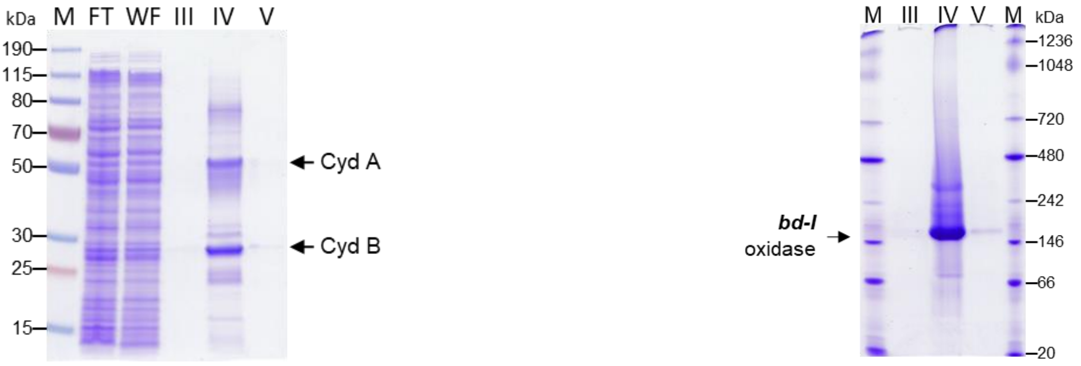

Figure S39: A) SDS PAGE in MES buffer; gradient polyacrylamid gel 4-12 %. M: marker; FT: Flow through; WF: wash fraction; III-V: Elution fractions 3-5; concentration was 8,7 mg/ml with a mass of 100 kDa. B) Clear Native PAGE 4-16 % Bis-Tris gel, stained with Coomassie blue: M: marker; III-V: *bd-I* type oxidase elution fractions III-V before dialysis; (8,7 mg/ml; 87 µM; 100 kDa).

7. Inhibitory assay profiles of HNQ-2 and HNQ-12

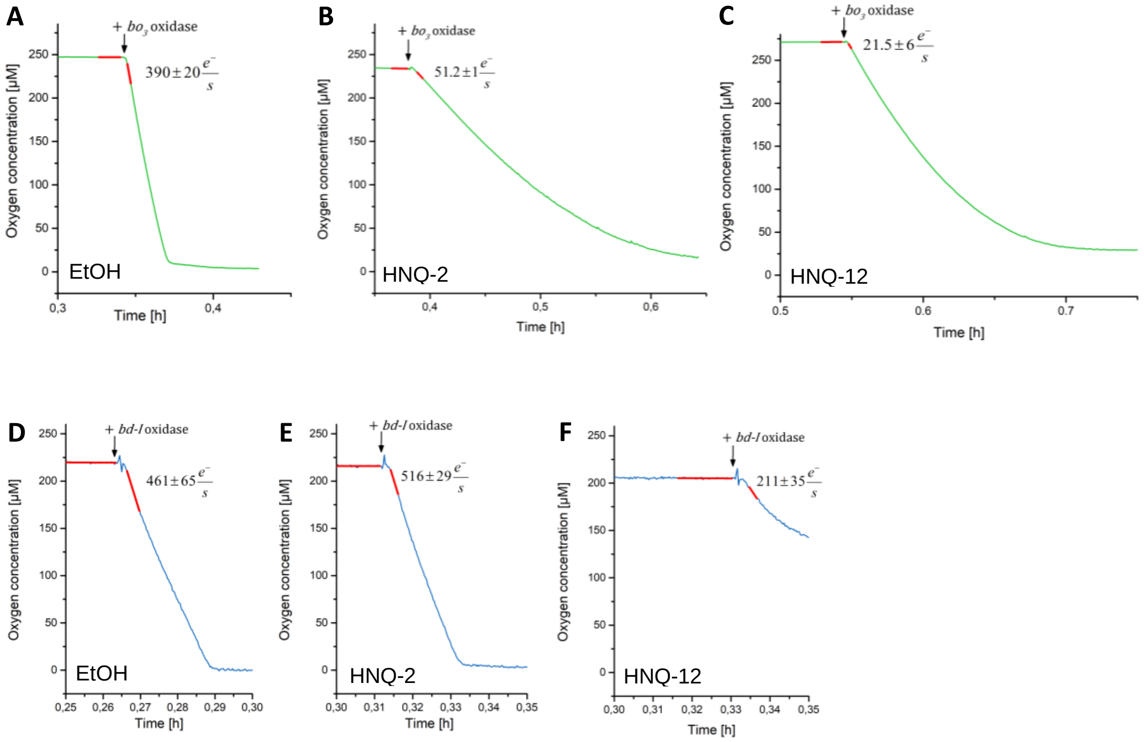

Figure S40: (A-F) Oxygen reductase activity of *cyt bo₃* (green) or *bd-I* type (blue) oxidase samples as indicated. Effects of 250 µM HNQ-2 dissolved in EtOH were determined polarographically using a Clark-type oxygen electrode. Red traces before and after enzyme addition represent slopes, used for turnover calculations. Turnover in electrons per second, mean of three independent measurements ± standard deviation.

Table S3: Inhibition of oxygen reduction activity in purified *E. coli bo*$_3$ and *bd-I* oxidase by HNQ analogues at 250 µM.

Compound	Residual activity for oxygen reduction [%]	
	Cytochrome ***bo***$_3$	Cytochrome ***bd-I***
**EtOH**	100 ± 2	100 ± 2
**KCN**	37.8 ± 5	101 ± 5
**HQNO**	4.42 ± 6	9.05 ± 1
**HNQ-1**	2.64 ± 12	70.2 ± 12
**HNQ-2**	21.8 ± 6	107 ± 6
**HNQ-3**	99.5 ± 12	110 ± 12
**HNQ-4**	84.8 ± 11	84.2 ± 11
**HNQ-5**	59.9 ± 7	117 ± 7
**HNQ-6**	27.3 ± 13	109 ± 13
**HNQ-7**	49.5 ± 7	106 ± 7
**HNQ-8**	101 ± 5	106 ± 5
**HNQ-9**	84.4 ± 17	110 ± 17
**HNQ-10**	69.1 ± 8	107 ± 8
**HNQ-11**	115 ± 13	111 ± 13
**HNQ-12**	13.1 ± 7	43.8 ± 7

8. Determination of apparent Ki of HNQ-6, HNQ-12 and HQNO

Method to determine k_i^{app}

Oxygen reduction activity of cyt. *bo₃* was determined by a Clark-type oxygen electrode for a series of inhibitor concentrations from 1 mM to 20 nM. Substrate concentration was kept constant at 200 µM ubiquinone-1 with the same buffer conditions as described earlier. The residual activity data were normalized to get the normalized-residual-activity values. In the next step the normalized-inhibitory-effect was calculated by subtraction of the normalized-residual-activity values from 1. The normalized-inhibitory-effect represents the y-ordinate as a function of the inhibitor concentration in a half logarithmic graph, see Fig. 5-7. The sigmoidal fit to the data points results in a curve where the turning point represents the concentration at which the normalized-inhibitory-effect is 0,5 or EC50. This concentration represents k_i^{app} for the tested inhibitor.

The sigmoidal fit uses the DoseResp – model or Hill-equation with the following form: $y = A1 + \frac{(A2-A1)}{(1+10^{(Logx0-x)p})}$

Where A1 (bottom) and A2 (top) represent the baseline- and maximum-response of the inhibitory effect, as marked in Fig. 5 and p represents the Hill-slope.

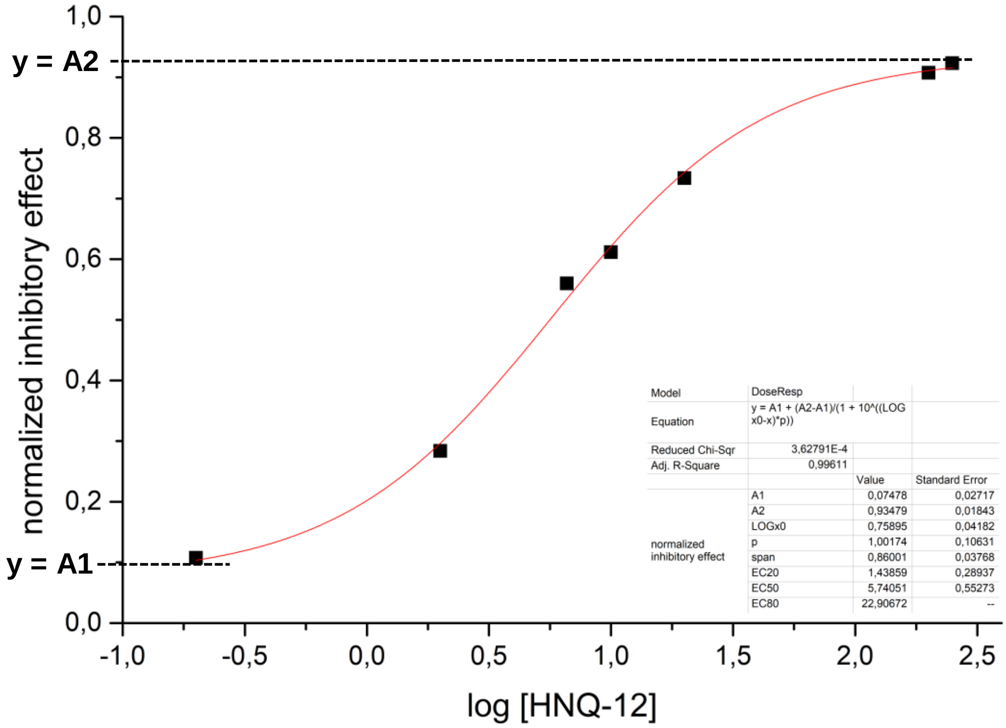

Figure S41: Determination of k_i^{app} by half-logarithmic plotting of the normalized-inhibitory-effect of HNQ-12 on cyt. *bo₃* over the inhibitor concentration (200 nM – 250 µM) and sigmoidal fit with DoseResp fit (red line). k_i^{app} for HNQ-12 is 5,7 ± 0,6 µM.

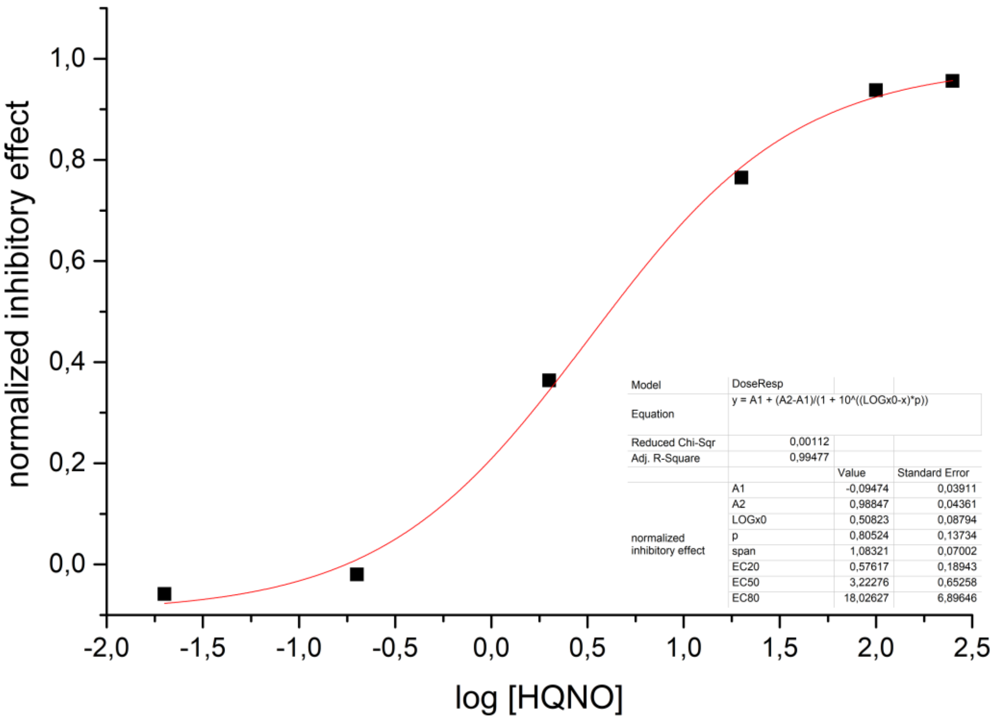

Figure S42: Determination of k_i^{app} by half-logarithmic plotting of the normalized-inhibitory-effect of HQNO on cyt. *bo₃* over the inhibitor concentration (20 nM – 250 µM) and sigmoidal fit with DoseResp Fit (red line). k_i^{app} for HQNO is 3,2 ± 0,7µM.

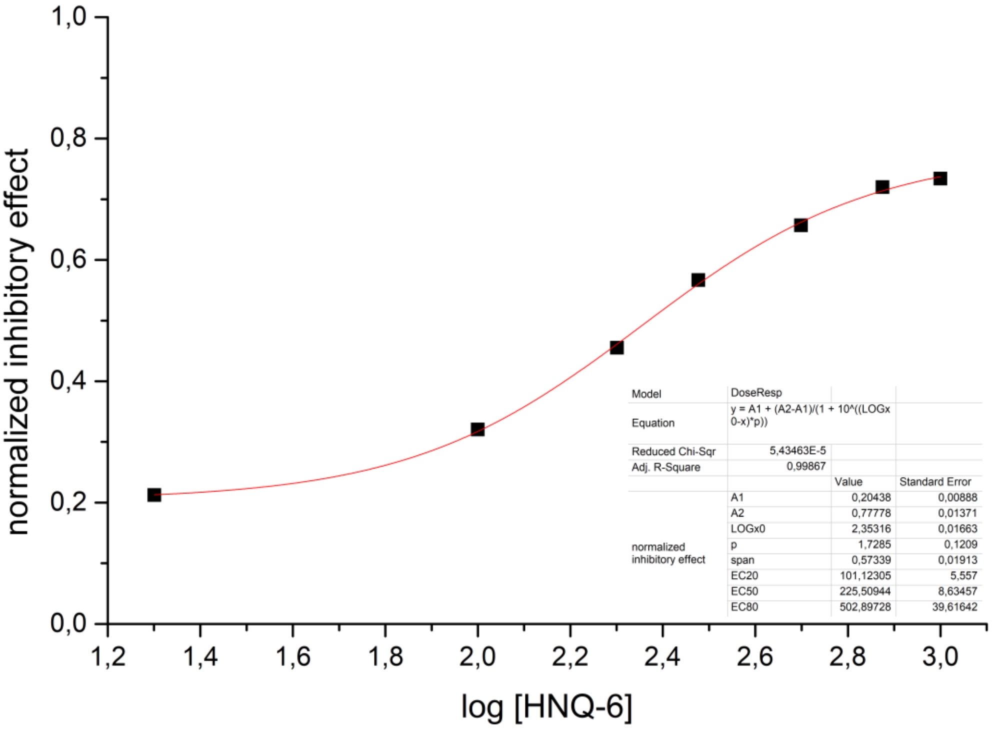

Figure S43: Determination of k_i^{app} by half-logarithmic plotting of the normalized-inhibitory-effect of HNQ-6 on cyt. *bo₃* over the inhibitor concentration (20 µM – 1 mM) and sigmoidal fit with DoseResp Fit (red line). k_i^{app} for HQNO is 225 ± 9 µM

9. 2D-interactions of HNQ-2,-6,-7 and HQNO in binding site of cyt *bo₃*

HNQ-2

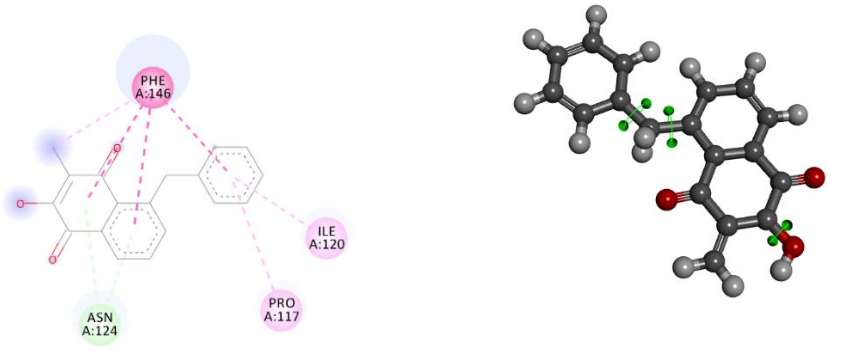

HNQ-6

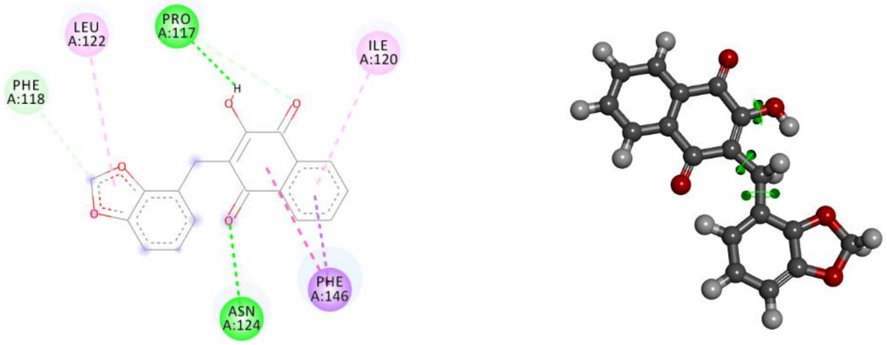

HNQ-7

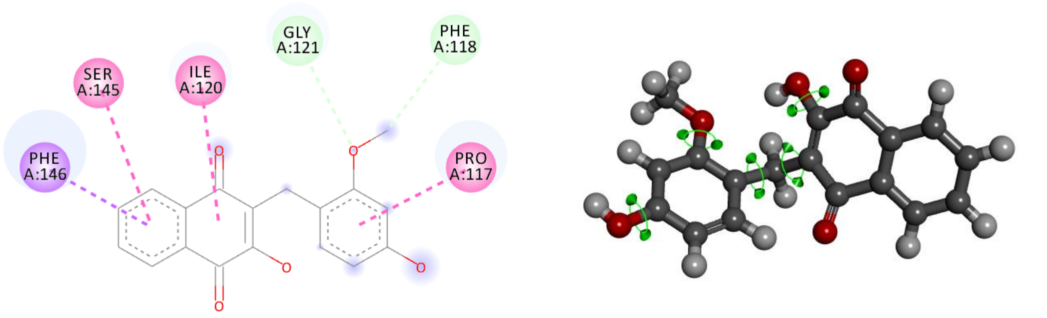

HQNO

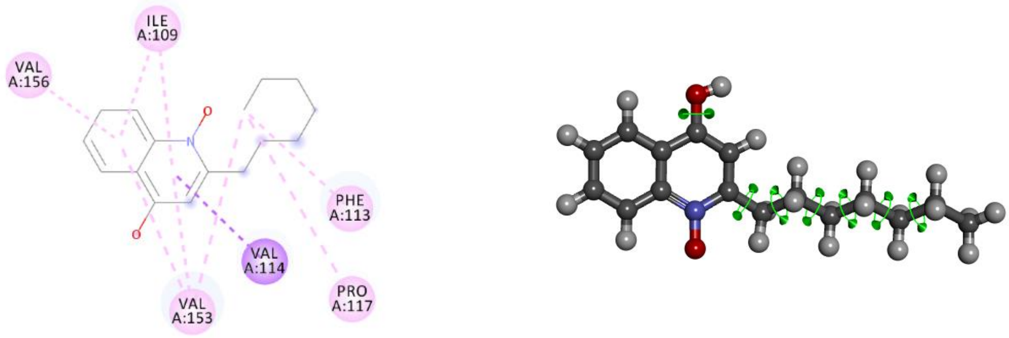

Figure S44: 2D- interactions of HNQ-2,6-7 and HQNO in higher affinity Q_H-binding site of cyt *bo*$_3$.

10. 2-Dimensional structures simulation by Gaussian semi- empirical PM3

HQNO

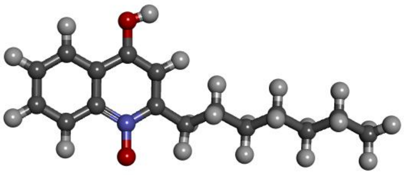

HNQ-1

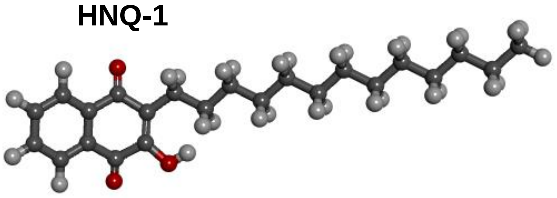

HNQ-2

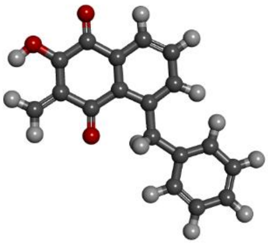

HNQ-3

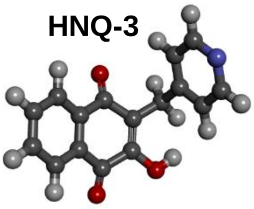

HNQ-4

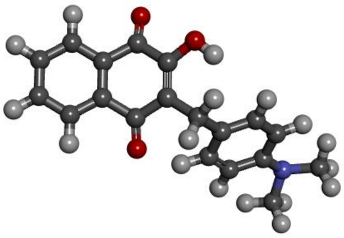

HNQ-5

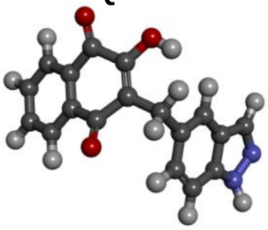

HNQ-6

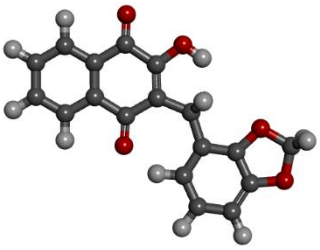

HNQ-7

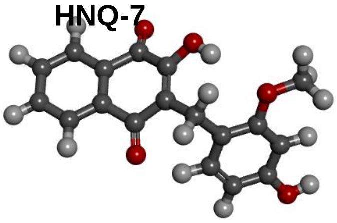

HNQ-8

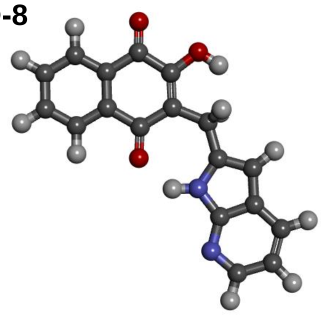

HNQ-10

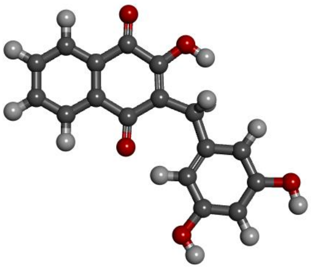

HNQ-11

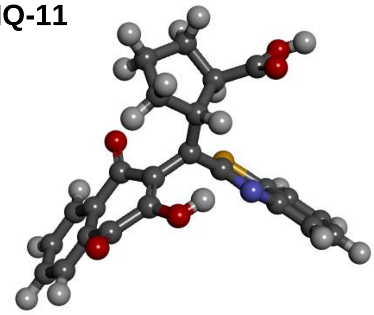

HNQ-12

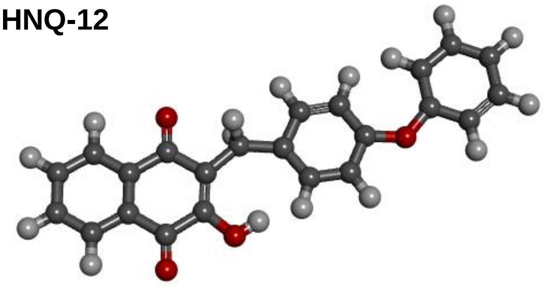

Figure S45: 2-Dimensional Structures: Generated for docking simulation by Gaussian Semi- empirical PM3.

11. Binding energies of tested HNQ compounds with *cyt bo$_3$* oxidase

Table S4: Binding energies of all tested HNQ-compounds and HQNO

**Compound**	**Binding energies [kcal/mol]**				
**HQNO**	-5.72	-5.41	-5.32	-5.24	-5.19
**HNQ-2**	-6.69	-6.68	-6.58	-6.63	-6.18
**HNQ-6**	-6.95	-6.81	-6.65	-6.49	-6.22
**HNQ-7**	-6.44	-6.22	-6.35	-6.28	-6.05
**HNQ-1**	-6.29	-3.21	-5.88	-5.51	-3.48
**HNQ-3**	-6.69	-6.19	-5.97	-5.81	-6.21
**HNQ-4**	-5.06	-5.89	-5.78	-5.43	-5.18
**HNQ-5**	-5.94	-5.23	-5.07	-4.92	-4.85
**HNQ-8**	-5.95	-5.67	-5.94	-5.38	-5.01
**HNQ-10**	-5.36	-5.06	-4.57	-4.46	-4.06
**HNQ-11**	-5.31	-5.16	-5.24	-5.17	-5.16
**HNQ-12**	-5.65	-5.52	-5.02	-4.08	-4.25

12. Substrate binding site for ubiquinol-8 in cyt *bo$_3$*

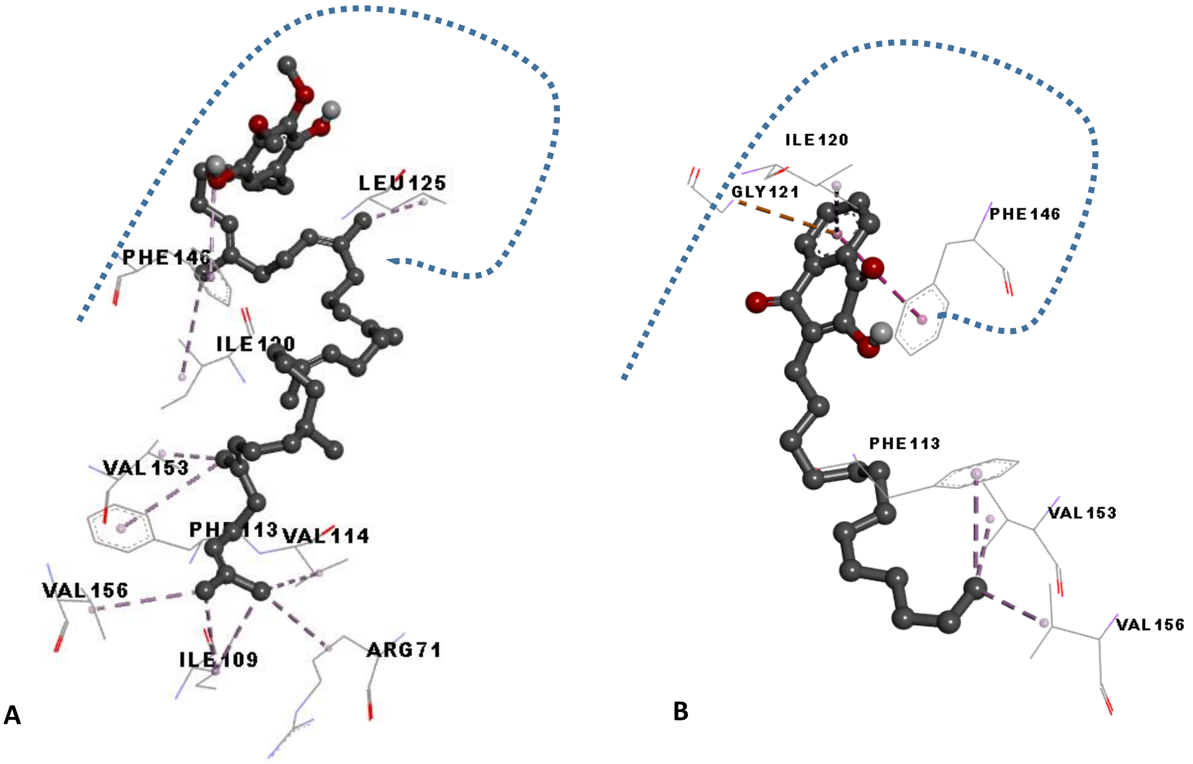

Figure S46: Substrate binding site for ubiquinol-8 (A) and HNQ-1 (B) in cyt *bo$_3$*; Hydrophobic binding site for HNQ derivatives where basic scaffolds are interacting (highlighted in blue).

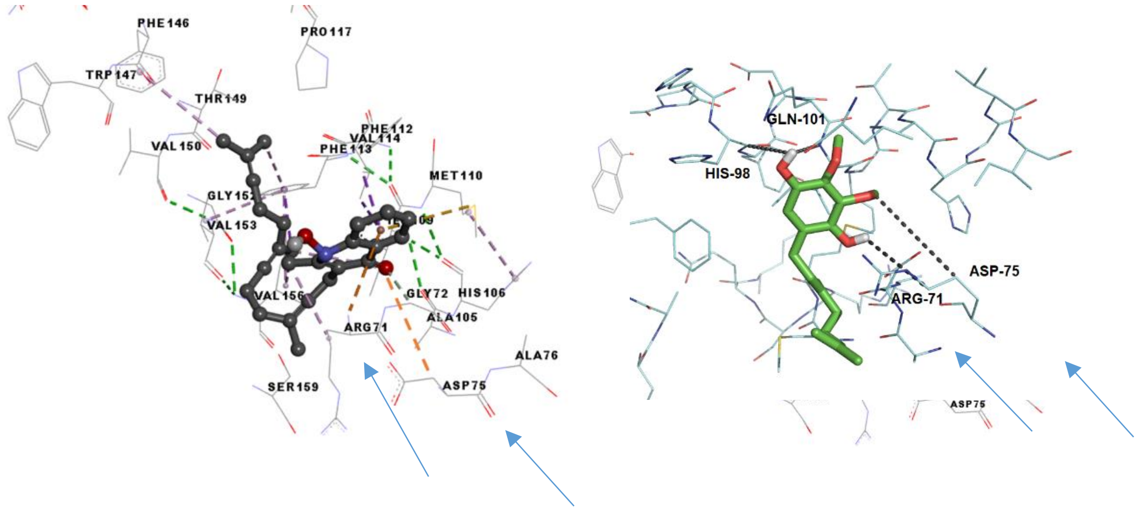

Figure S47: Proposed binding site of soluble analogue substrate ubiquinol-2 UQ2 within the subunit-I of cytochrome *bo*$_3$ ubiquinol oxidase (PDB: **1FFT**) (right). Docking of Aurachin C for comparison (left).

13. Binding energies of tested HNQ compounds with cyt. *bd* oxidase

Table S5 : Binding energies for best fives conformations of HNQ-derivatives, HQNO and Aurachin C with cytochrome *bd-I* oxidase PDB: **5DOQ**.

**Binding energies kcal/mol**					
**HQNO**	**-8.91**	**-8.87**	**-8.60**	**-8.61**	**-8.49**
**Aurachin C**	**-9.59**	**-8.16**	**-7.14**	**-5.71**	**-5.24**
HNQ-2	-3.19	-3.68	-3.12	-3.23	-3.18
HNQ-6	-4.83	-4.87	-4.19	-4.12	-3.22
HNQ-7	-3.19	-3.68	-3.12	-3.23	-3.18

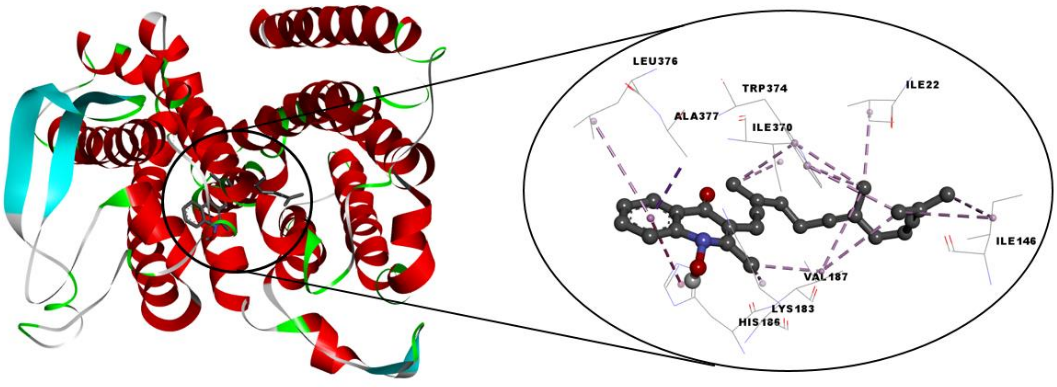

.

Figure S48 : Proposed binding site of Aurachin C within the subunit-I of cytochrome *bd* oxidase (PDB: **5DOQ**).

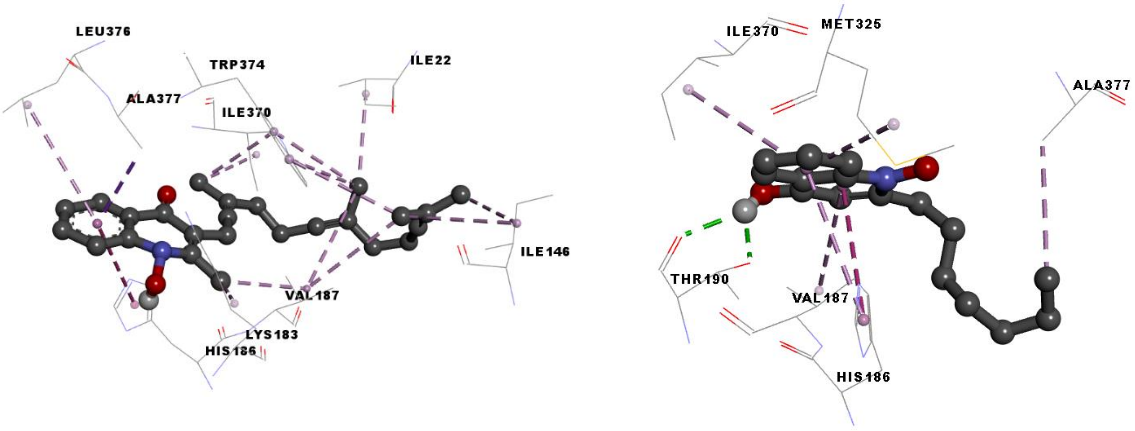

Figure S49 : Proposed 2D-docking interaction of Aurachin C1-10 (left) and HQNO (right) on the menaquinol binding loop of cytochrome *bd* oxidase (PDB: **5DOQ**).

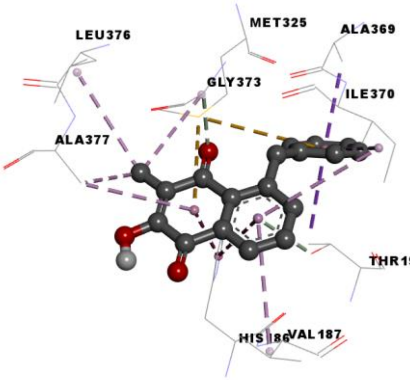

Figure S50 : Proposed 2D-docking interaction of HNQ-2 on the menaquinol binding loop of cytochrome *bd* oxidase (PDB: **5DOQ**).

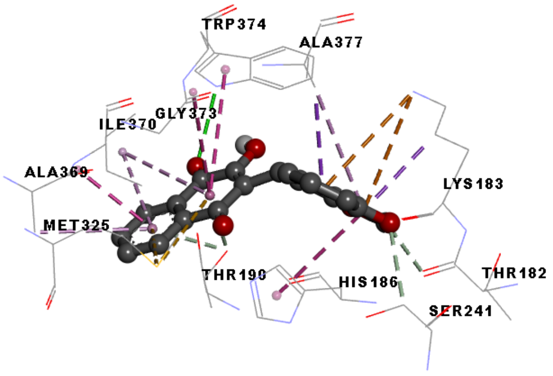

Figure S51 : Proposed 2D-docking interaction of HNQ-6 on the menaquinol binding loop of cytochrome *bd* oxidase (PDB: **5DOQ**).

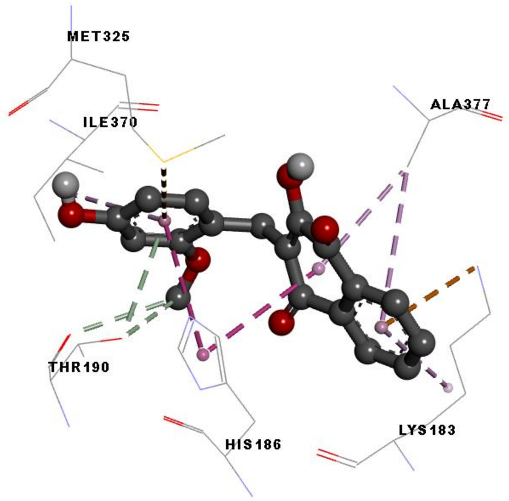

Figure S52 : Proposed 2D-docking interaction of HNQ-7 on the menaquinol binding loop of cytochrome *bd* oxidase (PDB: **5DOQ**).

14. Interactions of HQNO and HNQ-derivatives on the menaquinol binding loop of cytochrome *bd* oxidase (PDB: 5DOQ)

Interactions of HQNO: We found that for HQNO, central scaffold is involved in the carbon hydrogen interactions with THR-A-190 and Pi-Pi T shaped interactions (pink color) were observed with HIS-A-186. The aliphatic side chain of HQNO is involved in the alkyl and Pi-alkyl type of interactions with MET-A-325, VAL-A-187 and ILE-A370 respectively.

Interactions of HNQ-2: We found that for HNQ-2, the 3-hydroxynaphthalene-1,4-dione central scaffold is involved in the carbon hydrogen interactions with GLY-A-373 and THR-A-190 is involved in Pi-donor hydrogen interaction (cyan color). In addition to above, Pi-Pi T-shaped interactions (pink color) were observed with HIS-A-186, and MET-A-325 is involved in the Pi-sulfur interactions (yellow color). Some alkyl and Pi-alkyl (light pink) and Pi-sigma (violet color) type of interactions were observed with ALA-A-377, LEU-A-376, VAL-A-187, ALA-A-369 and THR-A-190 respectively.

Interactions of HNQ-6: Molecular docking insight of HNQ-6 suggests that the central naphthalene ring is involved in Pi-sigma interactions (violet color), Pi-donor (pink color) and Pi-cation (light pink color) with ALA-A-377, THR-A-190, SER-A-241, and THR-A-182, LYS-A-183 respectively. Some interesting amid-pi stacking and Pi-Pi T shaped interactions were observed with ALA-A-369, GLY-A-373 and HIS-A-186 respectively. In addition to this conventional hydrogen interactions (green color) and unconventional Pi-donor hydrogen binding interactions (cyan color) were observed in 1,4-dione carbonyl and hydroxyl moiety of HNQ-6 with TRP-A-374.

Interactions of HNQ-7: Hydroxynaphthoquinone HNQ-7 is involved in the Pi-Pi T-type stacking (pink color) with HIS-A-186 and some Pi-alkyl interactions (pink color) were observed with ALA-A377, ILE-A-370. In addition to above interacting Pi-sulfur type of interactions (yellow color) were observed with MET-A-325. The carbon-hydrogen and Pi-cation type of interactions were observed with the amino acid THR-A-190, LYS-A183 and MET-A-325 respectively.
